# Breaking the Selectivity Barrier of Single‐Atom Nanozymes Through Out‐of‐Plane Ligand Coordination

**DOI:** 10.1002/adma.202506480

**Published:** 2025-07-06

**Authors:** Seonhye Park, Kyu In Shim, Phuong Thy Nguyen, Daeeun Choi, Seongbeen Kim, Seung Yeop Yi, Moon Il Kim, Jeong Woo Han, Jinwoo Lee

**Affiliations:** ^1^ Department of Chemical and Biomolecular Engineering Korea Advanced Institute of Science and Technology (KAIST) 291 Daehak–ro, Yuseong–gu Daejeon 34141 Republic of Korea; ^2^ Department of Materials Science and Engineering Seoul National University Seoul 08826 Republic of Korea; ^3^ Department of BioNano Technology Gachon University 1342 Seongnamdae‐ro, Sujeong‐gu Seongnam Gyeonggi 13120 Republic of Korea

**Keywords:** biosensing, peroxidase‐like activity, point‐of‐care testing, single‐atom nanozyme, out‐of‐plane ligand coordination

## Abstract

Peroxidase (POD)‐like nanozymes have emerged as effective alternatives to natural enzymes owing to their stability and cost‐effectiveness in biosensors. In particular, single‐atom nanozymes (SAzymes) featuring Fe–N_4_ active sites have attracted significant attention for their high catalytic performance. However, their 2D exposed active sites result in limited reaction selectivity and strong pH dependence, restricting their functionality under neutral conditions. This study introduces Ru‐centered SAzymes coordinated out‐of‐plane with chlorine ligands (RuNC_Cl), achieving monofunctional POD‐like activity. RuNC_Cl exhibited remarkable POD‐like activity, which is 38‐fold greater than its catalase (CAT)‐like activity, indicating strong suppression of the competing CAT‐like reaction. Density functional theory calculations and Bader charge analysis of RuNC_Cl reveal that repulsive forces preventing secondary H_2_O_2_ adsorption contribute to an increased energy barrier for the CAT‐like reaction. This selective POD‐like activity enables the precise detection of multiple biomarkers through a one‐pot cascade reaction under near‐neutral conditions. This advancement paves the way for the precise regulation of reaction pathways, enhancing the practicality of nanozymes for biosensing and related applications.

## Graphical abstract

Here, Ru SAzyme with out‐of‐plane chlorine ligands is reported as a monofunctional, high‐performance peroxidase‐like nanozyme. Incorporating out‐of‐plane ligand coordination emulates enzyme selectivity and regulates reaction pathways for an identical substrate. The optimized Ru SAzyme exhibits exceptional peroxidase‐selective activity (38‐fold higher than its catalase‐like activity) and demonstrates practical applicability for simultaneous biomarker quantification under near‐neutral conditions.

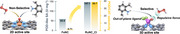

## 1 Introduction

Point‐of‐care (POC) testing, performed at or near the site of patient care, necessitates rapid and precise diagnostics.^[^ ^1^, ^2^ ^]^ In this context, colorimetric biosensors based on natural enzymes have garnered substantial attention owing to their rapid response and broad applications, including medical diagnosis, virus detection, food analysis, and environmental monitoring.^[^ ^3^, ^4^, ^5^, ^6^ ^]^ In particular, horseradish peroxidase (HRP) is widely used in biosensors for its high sensitivity in detecting H_2_O_2_ by catalyzing the oxidation of colorimetric substrates.^[^ ^7^ ^]^ Furthermore, cascade reactions based on HRP coupled with various oxidase enzymes have expanded its capabilities for detecting the corresponding biomarkers with high sensitivity.^[^ ^8^ ^]^ However, the inherent vulnerability of natural enzymes to denaturation imposes substantial expenses on production and storage, limiting the competitiveness of POC biosensors.^[^ ^9^, ^10^ ^]^ To address these limitations, peroxidase (POD)‐like nanozymes, which are nanomaterials with POD‐like activities, have emerged as promising alternatives owing to their enhanced stability and cost‐effectiveness in biosensing applications.^[^ ^11^ ^]^ To achieve high catalytic performance, iron‐centered single‐atom nanozymes (Fe SAzymes), have attracted significant interest.^[^ ^12^, ^13^ ^]^ Fe SAzymes feature Fe–N_4_ active sites that mimic the heme prosthetic group of HRP, enable an enhanced catalytic turnover rate (*k*_cat_) comparable to that of HRP.^[^ ^14^, ^15^, ^16^ ^]^

Nonetheless, the practical application of Fe SAzymes is limited by their pH limitation and low reaction selectivity. Most natural oxidases function optimally under neutral conditions, whereas the POD‐like activity of Fe SAzymes is restricted to acidic conditions.^[^ ^17^, ^18^ ^]^ In order to maximize their catalytic efficiency, pH adjustments with buffer transfers are required for subsequent reactions. Additionally, when assays are conducted under near‐neutral conditions for one‐pot biomarker detection, Fe SAzymes exhibit competing catalase (CAT)‐like activity.^[^ ^19^ ^]^ This competition between POD‐like and CAT‐like pathways reduces the available H_2_O_2_ generated by natural oxidases, thereby deteriorating the biomarker detection accuracy.^[^ ^20^, ^21^ ^]^ Notably, heme also functions as a prosthetic group for various oxidoreductases, including CAT.^[^ ^22^, ^23^, ^24^ ^]^ The catalytic selectivity of these natural enzymes is attributable to their protein frame, in which the heme group is enclosed. In contrast to natural POD, conventional Fe SAzymes possess exposed Fe–N_4_ sites, lacking ligand coordination essential for fine‐tuning reaction pathways. Therefore, these SAzymes inherently exhibit limitations in regulating reaction selectivity for the identical substrate. Although researchers have attempted to enhance selectivity by mimicking amino acid residues, these approaches often involve laborious post‐synthetic processes to affix ligands after the formation of exposed active sites.^[^ ^25^, ^26^, ^27^, ^28^ ^]^ Furthermore, while these strategies can enhance the affinity with the target substrate, it remains still insufficient to control the reaction pathways for the same substrate. In this vein, a novel approach is required for developing a POD‐selective nanozyme with suppressed CAT‐like activity for biosensing applications.

**Scheme 1:**
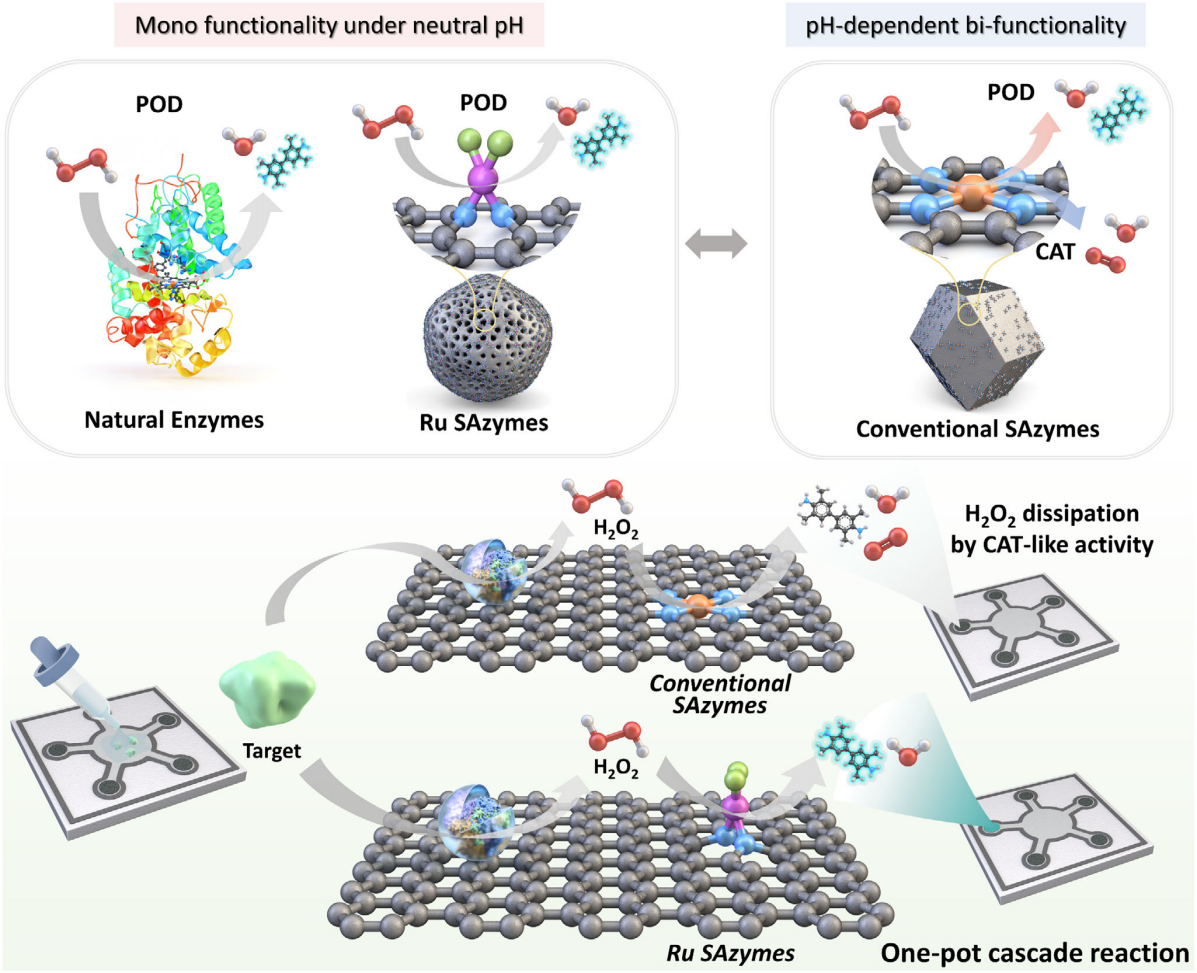
Schematic illustration of POD‐selective activity of Ru SAzymes and its application for the one‐pot detection of biomarkers under neutral pH.

Herein, we developed Ru SAzymes featuring 3D active sites coordinated out‐of‐plane ligands (referred to as 3D active sites), overcoming the limitations of conventional Fe SAzymes. To maximize the POD‐like performance of Ru SAzymes while minimizing Ru consumption, we synthesized SAzymes using three different Ru precursors to identify the optimal coordinating ligand and systematically compare their structures and catalytic behaviors. Ru SAzymes incorporating three different ligands were synthesized, and the Ru SAzyme with Cl ligands (RuNC_Cl) was noted to exhibit the highest POD‐selective activity. The incorporation of out‐of‐plane ligand coordination substantially inhibits CAT‐like activity, enabling high selectivity toward POD‐like reaction. Theoretical evaluations demonstrated that the CAT‐like reaction on RuNC_Cl had a significantly higher energy barrier than that of the POD‐like reaction, leading to a preferential shift toward the POD‐like pathway. Leveraging its high POD‐selective activity, RuNC_Cl was incorporated into paper‐based microfluidic devices (RuNC_Cl@µPAD), enabling precise quantification of multiple biomarkers. These findings highlight that adjusting the out‐of‐plane ligand coordination in nanozymes enables mimicking monofunctional POD‐like activity. Furthermore, the proposed strategy for regulating reaction pathways can expand the potential applications of nanozymes, specifically in fields requiring precision, such as disease diagnosis and treatment (**Scheme** **1**).

Ru SAzymes pose 3D active sites with out‐of‐plane ligand coordination, playing a crucial role in POD‐selective activity. The detailed description of the POD‐selective activity of Ru SAzymes can be found in Figure S1 and Note S1 (Supporting Information). Mirroring the mono functionality of natural enzymes, the absence of competitive CAT‐like activity in Ru SAzymes ensures that selective POD‐like reactions proceed under neutral conditions. In contrast, conventional SAzymes with 2D exposed active sites (referred to as 2D active sites) exhibit pH‐dependent bifunctionality. Under neutral conditions, their CAT‐like activity leads to H_2_O_2_ dissipation, hindering accurate detection. The elimination of this interference in Ru SAzymes enables the direct detection of biomarkers via cascade reactions with natural oxidases, obviating the cumbersome step such as media change. The simultaneous detection of multiple biomarkers under neutral conditions demonstrates the practicality of Ru SAzymes for POC testing.

## 2 Results

### 2.1 Synthesis and Characterization of Ru SAzymes

**Figure 1:**
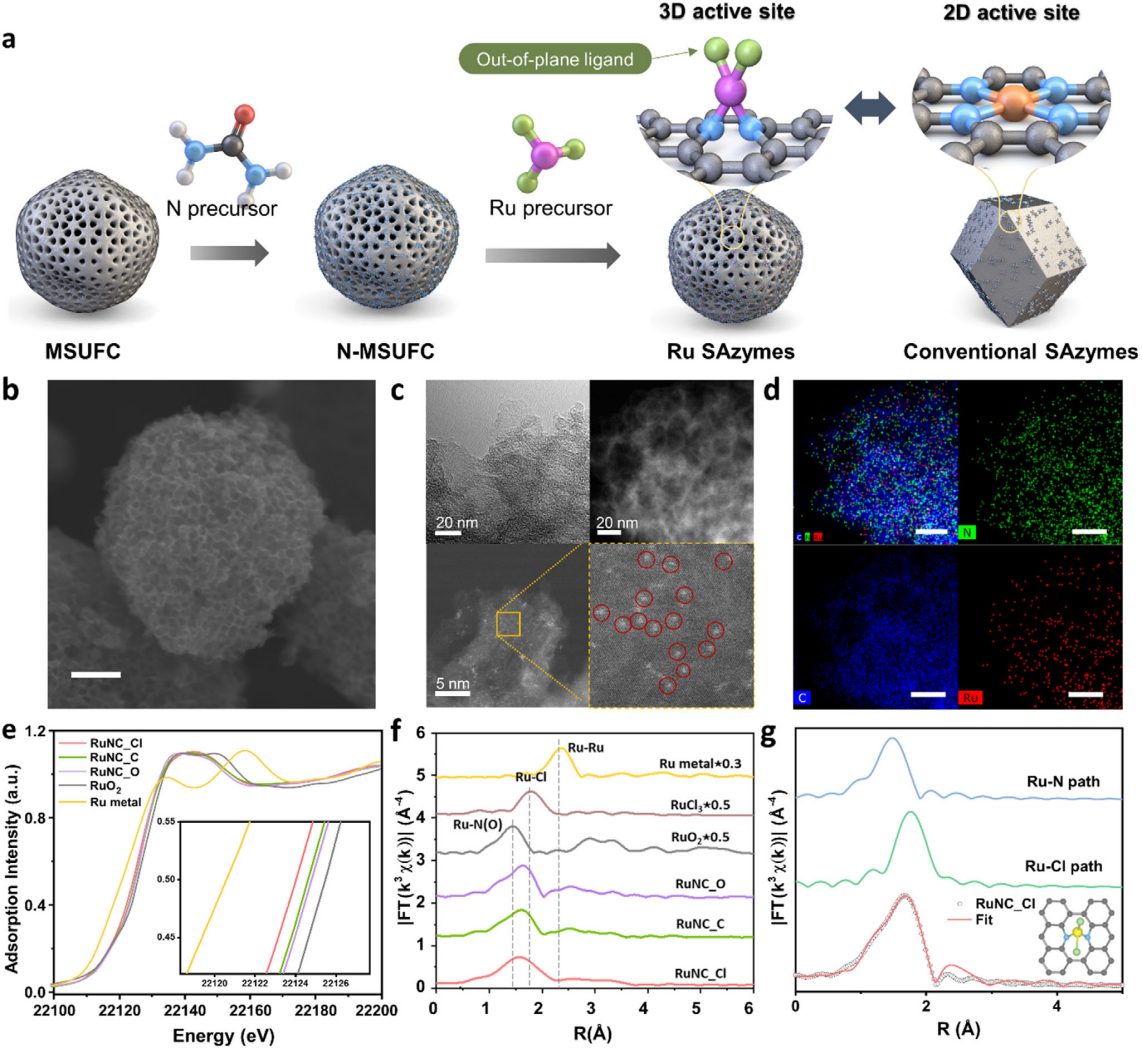
Synthesis and structure characterization of the Ru SAzymes.a) Schematic images of synthesizing RuNC_Cl. b) SEM image of RuNC_Cl (scale bar = 100 nm). c) TEM images of RuNC_Cl (the upper two images) and Aberration‐corrected atomic resolution HAADF‐STEM images of RuNC_Cl (the lower two images). d) EDX mapping of the RuNC_Cl (Ru: red, C: blue, N: green). e) The normalized XANES spectra at Ru K‐edge for Ru SAzymes and reference samples. f) The corresponding Fourier‐transformed EXAFS spectra of Ru K‐edge signals. g) Fourier‐transformed EXAFS fitting result of RuNC_Cl.

Ru SAzymes with out‐of‐plane ligand coordination were synthesized to investigate how 3D active sites and their surrounding ligands regulate reaction pathways and reactivity. Atomically dispersed Ru active sites were incorporated into mesoporous carbon by manipulating the type of surrounding ligands through different Ru precursors. Specifically, nitriding was performed through the thermal decomposition of urea on mesocellular foam carbon (MSUFC) to enhance interactions between the Ru precursor and carbon support.^[^ ^29^ ^]^ Transmission electron microscopy (TEM) images of both MSUFC and nitrided MSUFC (N‐MSUFC) clearly revealed well‐developed mesoporous architectures (Figure S2, Supporting Information). Ruthenium chloride (RuCl_3_) was introduced onto N‐MSUFC via wet impregnation to minimize the usage of the precursors and production cost of SAzyme. Subsequently, Ru loaded N‐MSUFC was annealed at 200 °C to obtain RuNC_Cl. During synthesis, single atomic Ru sites were well‐dispersed while preserving the ligands of the Ru precursor,^[^ ^30^ ^]^ as shown in **Figure** **1**a. RuNC_O and RuNC_C were prepared using Ru precursors containing Ru–O and Ru–C bonds, respectively. Ru nanozymes treated with high‐temperature annealing were also prepared, and the corresponding analyses and explanations are provided in Figure S3 and Note S2 (Supporting Information).

Scanning electron microscopy (SEM) was performed to characterize the structure of the synthesized nanoparticles (Figure 1b; Figure S4, Supporting Information). The SEM images confirmed that all Ru SAzymes exhibited mesoporous structures with particle sizes ranging between 300 nm and 400 nm. A Brunauer–Emmett–Teller (BET) analysis demonstrated that Ru SAzymes exhibited similar surface areas and pore sizes (Figure S5 and Table S1, Supporting Information). Dynamic light scattering measurements confirmed that Ru SAzymes were homogeneously dispersed, with a hydrodynamic diameter of approximately 350 nm (Figure S6, Supporting Information), consistent with the SEM observations. Additionally, zeta potential measurements consistently indicated negative values for Ru SAzymes (Figure S7, Supporting Information).

TEM images confirmed that the three Ru SAzymes showed mesoporous structure, without any discernible formation of Ru nanoparticles (Figure 1c and Figure S4, Supporting Information). Moreover, X‐ray diffraction (XRD) patterns of Ru SAzymes displayed only amorphous carbon peaks, with no signals for Ru nanoparticles detected (Figure S8, Supporting Information). High‐angle annular dark‐field scanning transmission electron microscopy (HAADF‐STEM) was further confirmed the atomically distributed state of Ru, marked by red circles in Figure 1c. The Ru loading content in RuNC_Cl was 1.95 wt%, measured using inductively coupled plasma mass spectrometry (ICP‐MS) (Table S2, Supporting Information). Energy‐dispersive X‐ray spectroscopy (EDX) mapping was also measured to verify the homogeneous distribution of Ru and N (Figure 1d). Due to the spectral overlap between the Cl Kα (2.62 keV) and Ru Lα (2.56 keV) emission lines,^[^ ^31^ ^]^ elemental mapping of Ru was conducted using the Ru Kα line (19.23 keV).^[^ ^32^ ^]^ The HAADF‐STEM images also revealed atomically dispersed Ru active sites in both RuNC_O and RuNC_C (Figure S9, Supporting Information).

The surface chemical state of RuNC_Cl was examined using X‐ray photoelectron spectroscopy (XPS). The N 1s spectrum of N‐MSUFC exhibited a distinct peak at ∼400.0 eV (Figure S10, Supporting Information) corresponding to amine group, which was retained in the Ru SAzymes prepared under low‐temperature annealing (Figures S11 and S12, Supporting Information). The presence of Cl was clarified through Cl 2p XPS analysis, which demonstrated the retention of Cl ligands (Figure S11c, Supporting Information). The Ru 3p XPS spectra exhibited doublet peaks corresponding to Ru 3p_1/2_ and Ru 3p_3/2_. A consistent shift toward higher binding energy was observed for Ru SAzymes relative to metallic Ru. Specifically, while the Ru 3p_3/2_ peak for Ru metal appeared at 461.2 eV,^[^ ^33^ ^]^ those for RuNC_Cl, RuNC_C, and RuNC_O were located at 462.71, 463.26, and 463.39 eV, respectively (Figure S13, Supporting Information). This gradual shift toward higher binding energies indicated the presence of Ru atoms in positively charged Ru species (Ru^δ+^).^[^ ^34^, ^35^ ^]^

X‐ray absorption near‐edge structure (XANES) and extended X‐ray absorption fine structure (EXAFS) analyses were performed to clarify the structure of Ru active sites. The XANES spectra for Ru SAzymes were calibrated using Ru foil as a reference. Since Ru is known to exhibit CAT‐like activity when it forms structural clusters and exists in a metallic state,^[^ ^36^ ^]^ it is crucial to confirm that Ru remains atomically dispersed and retains an oxidized state. Ru K‐edge XANES spectra showed that the half‐edge positions of the Ru SAzymes were between those of Ru metal and RuO_2_, corresponding to oxidation states from Ru(0) to Ru(+4) (Figure 1e). Specifically, the half‐edge positions for RuNC_Cl, RuNC_C, and RuNC_O were located at 22124.52, 22125.12, and 22125.32 eV, respectively. Notably, RuNC_Cl exhibited slightly lower half‐edge energy, suggesting a comparatively lower oxidation state, which is consistent with the Ru 3p_3/2_ peak shifts observed in the XPS results. In the EXAFS spectra, the Fourier‐transformed spectra (*k* ^3^‐weighted) in R‐space of Ru foil displayed a distinct peak at approximately 2.39 Å, which corresponds to Ru–Ru scattering.^[^ ^37^ ^]^ The absence of a peak at this position for Ru SAzymes indicated that Ru clusters were not formed. Furthermore, RuNC_Cl exhibited a primary peak at 1.60 Å, attributable to Ru–N scattering. These peaks shifted slightly toward a larger R‐value compared with that of RuO₂, suggesting the presence of ligands bonded to Ru (Figure 1f). Wavelet transforming (WT) of EXAFS was further conducted on Ru foil, RuO_2_, the three Ru precursors (Ru_Cl, Ru_O, Ru_C), and the three Ru SAzymes. As shown in Figure S14 (Supporting Information), Ru foil exhibited a distinct single‐scattering peak at *r* ≈ 2.4 Å, *k* ≈ 9.3 Å^−1^, corresponding to Ru–Ru coordination. In contrast, RuO_2_ displayed a Ru–O peak at *r* ≈ 1.5 Å, *k* ≈ 5.1 Å^−1^ and a prominent multiple‐scattering peak at *r* ≈ 3.3 Å, *k* ≈ 10.6 Å^−1^, attributed to Ru–O–Ru paths. These characteristic features were absent in Ru SAzymes, indicating that Ru atoms are atomically dispersed.^[^ ^37^ ^]^ RuNC_Cl exhibited a peak at *r* ≈ 1.6 Å, *k* ≈ 4.5 Å^−1^, shifted relative to the corresponding features in RuNC_C and RuNC_O. This shift suggested that Ru in RuNC_Cl is coordinated to a heavier ligand (Cl).^[^ ^38^ ^]^ While Ru_Cl exhibited a strong Ru–Cl–Ru double‐scattering signal at high r and k values, RuNC_Cl showed only a mid‐range signal likely arising from Ru–N–C paths, indicating the absence of Ru–Cl–Ru coordination.^[^ ^39^ ^]^ In addition, RuNC_C and RuNC_O retained double‐scattering signals attributable to residual organic ligands, indicating partial ligand retention after heat treatment.^[^ ^40^ ^]^ The best‐fitting results revealed that the central Ru sites in RuNC_Cl predominantly adopted a RuN_2_Cl_2_ configuration, with two Cl atoms in an out‐of‐plane coordination geometry (Figure 1g; Table S3, Supporting Information). The preservation of precursor‐derived ligands was also observed in RuNC_O and RuNC_C (Figure S15, Supporting Information). Specifically, Ru sites in RuNC_C were coordinated with one N atom and four C atoms in the first shell, while those in RuNC_O were bonded to one N atom and four O atoms in the first shell. Overall, these results confirmed that Ru SAzymes prepared through low‐temperature heat treatment featured single atomic Ru sites, as they preserved precursor‐derived ligands while forming additional bonds with nitrogen.

### 2.2 Evaluation of POD‐Like Performances of Ru SAzymes

**Figure 2:**
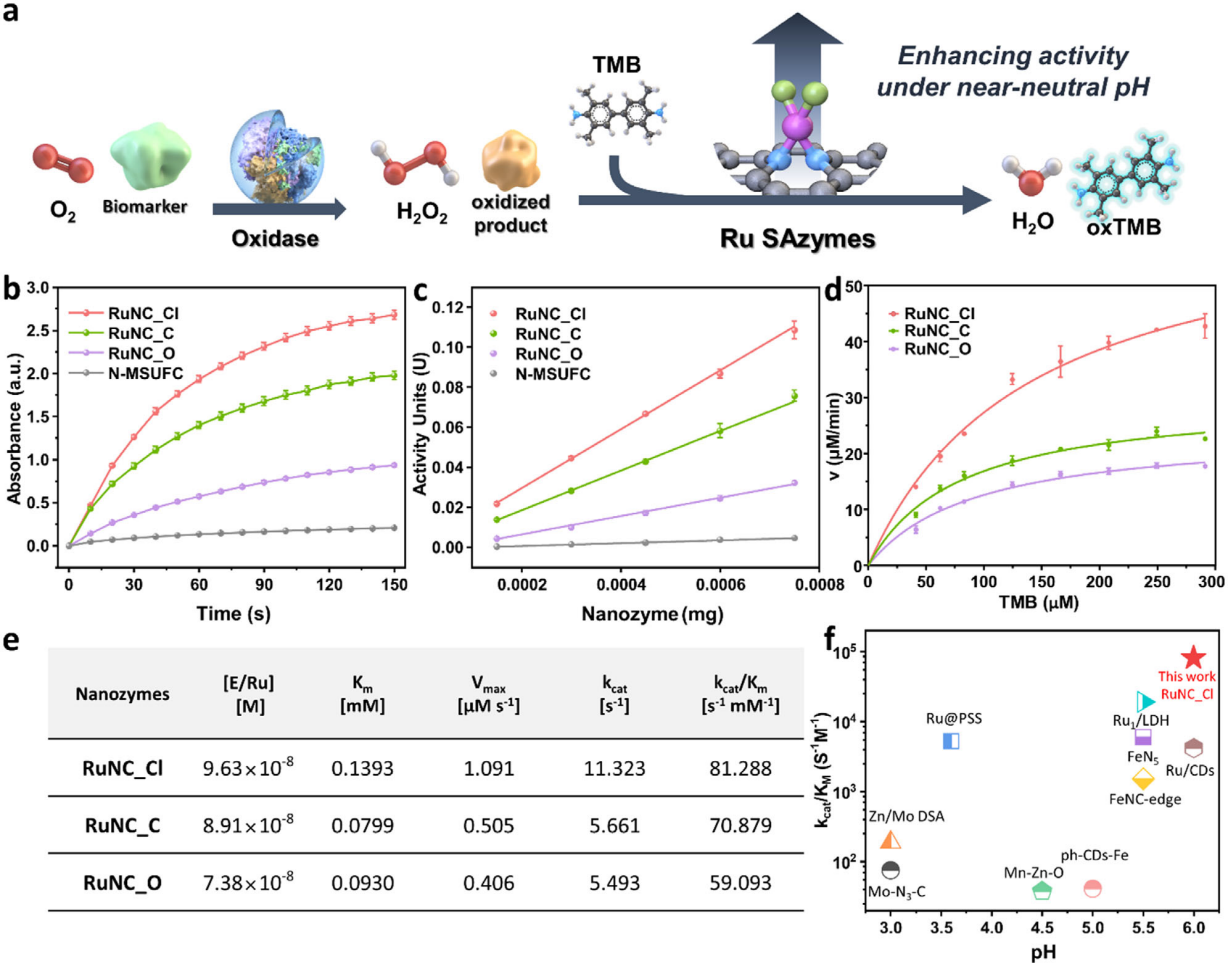
POD‐like performance of Ru SAzymes. a) Schematic illustration of POD‐like reaction of Ru SAzymes b) Absorbance at 652 nm versus time curves. c) Plot of the POD‐like specific activity of Ru SAzymes. d) Steady‐state kinetic assay plots using TMB as substrate. e) Comparison of catalytic efficiency (*k*_cat_/*K*_M_) versus pH with reported POD‐like nanozymes. f) Kinetic performance results of the Ru‐SAzymes.[E/Ru]: Molar concentration of ruthenium, *K*_M_: Michaelis‐Menten constant, *v_max_*: Maximum reaction velocity, *k*_cat_: catalytic rate constant, *k*_cat_/*K*_M_: catalytic efficiency.

As depicted in **Figure** **2**a, oxidases first oxidize the corresponding biomarkers, producing H_2_O_2_. The generated H_2_O_2_ is subsequently reduced in a reaction catalyzed by POD‐like nanozymes, while oxidizing the substrate.^[^ ^41^ ^]^ Conventional POD‐like nanozymes are restricted to acidic pH conditions, leading to diminished tandem reaction rates under neutral conditions. Considering that most oxidases function optimally at neutral pH, enhancing POD‐like activity under near‐neutral conditions is crucial for the effective detection of target biomarkers in tandem reactions. POD‐like activities were evaluated using a standardized assay based on the oxidation of 3,3′,5,5′‐tetramethylbenzidine (TMB).^[^ ^42^ ^]^ In this reaction, H_2_O_2_ was reduced into H_2_O while TMB was converted into oxidized TMB (oxTMB), which displayed an absorption peak at 652 nm.^[^ ^43^, ^44^ ^]^ The optimal pH for the POD‐like reaction was first determined using RuNC_Cl, which showed maximal absorbance under near‐neutral conditions (pH 6.0) (Figure S16, Supporting Information). Based on this result, subsequent evaluations of the POD‐like activities were conducted at pH 6.0. As the POD reaction proceeded, the absorbance at 652 nm increased progressively (Figure 2b). RuNC_Cl exhibited the highest activity, followed by RuNC_C and RuNC_O, while N‐MSUFC showed the lowest activity. The specific activity (SpA), representing the enzymatic activity per unit mass, was calculated to quantitatively assess the enzymatic performance. Among the Ru SAzymes, RuNC_Cl demonstrated the highest specific activity of 147.1 U·mg^−1^, which was 20 times greater than that of N‐MSUFC. This was followed by RuNC_C, which demonstrated a specific activity of 98.8 U·mg⁻^1^, and RuNC_O, with a value of 45.8 U·mg⁻^1^ (Figure 2c).

A steady‐state kinetic analysis was conducted using phosphate buffer (pH 6.0) to determine the kinetic parameters and catalytic performance of the active sites (Figure 2d). The Michaelis–Menten kinetics toward TMB oxidation were observed for Ru SAzymes. Figure 2e shows the calculated Ru SAzyme concentrations and kinetic parameters for TMB oxidation. Among the Ru SAzymes, RuNC_Cl showed the highest *k*_cat_ value of 11.323 s^−1^, confirming its superior catalytic turnover rate. Notably, the *k*_cat_ value of RuNC_Cl was approximately two‐fold higher than those of RuNC_C (*k*_cat_ = 5.661 s^−1^) and RuNC_O (*k*_cat_ = 5.493 s^−1^), indicating faster reaction rate of TMB at the active sites of RuNC_Cl. The *k*_cat_/*K*_M_ value, which accounts for both substrate affinity and reaction rate, was used to evaluate the catalytic efficiency. RuNC_Cl exhibited the highest *k*_cat_/*K*_M_ value of 81.288 s^−1^·mm ^−1^, followed by RuNC_C (*k*_cat_/*K*_M_ = 70.879 s^−1^·mm ^−1^) and RuNC_O (*k*_cat_/*K*_M_ = 59.093 s^−1^·mm ^−1^). In addition, RuNC_Cl exhibited the highest kinetic parameters for the H_2_O_2_ substrate (Figure S17 and Table S4, Supporting Information). The *k*_cat_/*K*_M_ value of RuNC_Cl was 0.0116 s^−1^ mm ^−1^, which was 1.5‐fold higher than that of RuNC_C (*k*_cat_/*K*_M_ = 0.0082 s^−1^·mm ^−1^) and three‐fold higher than that of RuNC_O (*k*_cat_/*K*_M_ = 0.0043 s^−1^·mm ^−1^). These results demonstrated that tuning the Ru active site enhanced catalytic activity, and the Ru active sites coordinated with Cl ligands exhibited superior POD‐like performance. To compare the enzymatic performance of the proposed SAzymes with existing nanozymes, the *k*_cat_/*K*_M_ values were compared in relation to the measured pH (Figure 2f). The results indicated that RuNC_Cl exhibited the highest *k*_cat_/*K*_M_ value among nanozymes evaluated under near‐neutral pH conditions.^[^ ^45^, ^46^, ^47^, ^48^, ^49^, ^50^, ^51^, ^52^, ^53^ ^]^ Consequently, the introduction of Cl as a ligand to the Ru active center resulted in the highest POD‐like SpA and *k*_cat_/*K*_M_, thereby maximizing the utilization of Ru.

In addition, the Ru 3p XPS profiles of the Ru SAzymes were measured after three consecutive reaction cycles to further evaluate the stability of their Ru active sites during the POD‐like reactions (Figure S18, Supporting Information). The Ru 3p_3/2_ peak position of RuNC_Cl exhibited a negligible shift from 462.71 to 462.70 eV after the reaction cycles. Comparable observations were made for RuNC_C and RuNC_O, which exhibited slight peak shifts of 0.07 and 0.05 eV, respectively. These results indicated that the valence state of Ru is generally well preserved over multiple reaction cycles. Inductively coupled plasma optical emission spectroscopy (ICP‐OES) analysis of the supernatants after each reaction cycle revealed no detectable Ru ions (0.00 ppm), providing additional evidence that the Ru active sites in the Ru SAzymes remained stable over multiple catalytic cycles (Table S5, Supporting Information). These findings highlight the exceptional POD‐like activity and stability of RuNC_Cl under near‐neutral conditions, indicating its potential for application in cascade‐reaction‐based biosensing systems.

### 2.3 Comparison of Reaction Selectivity of RuNC_Cl and FeNC

**Figure 3:**
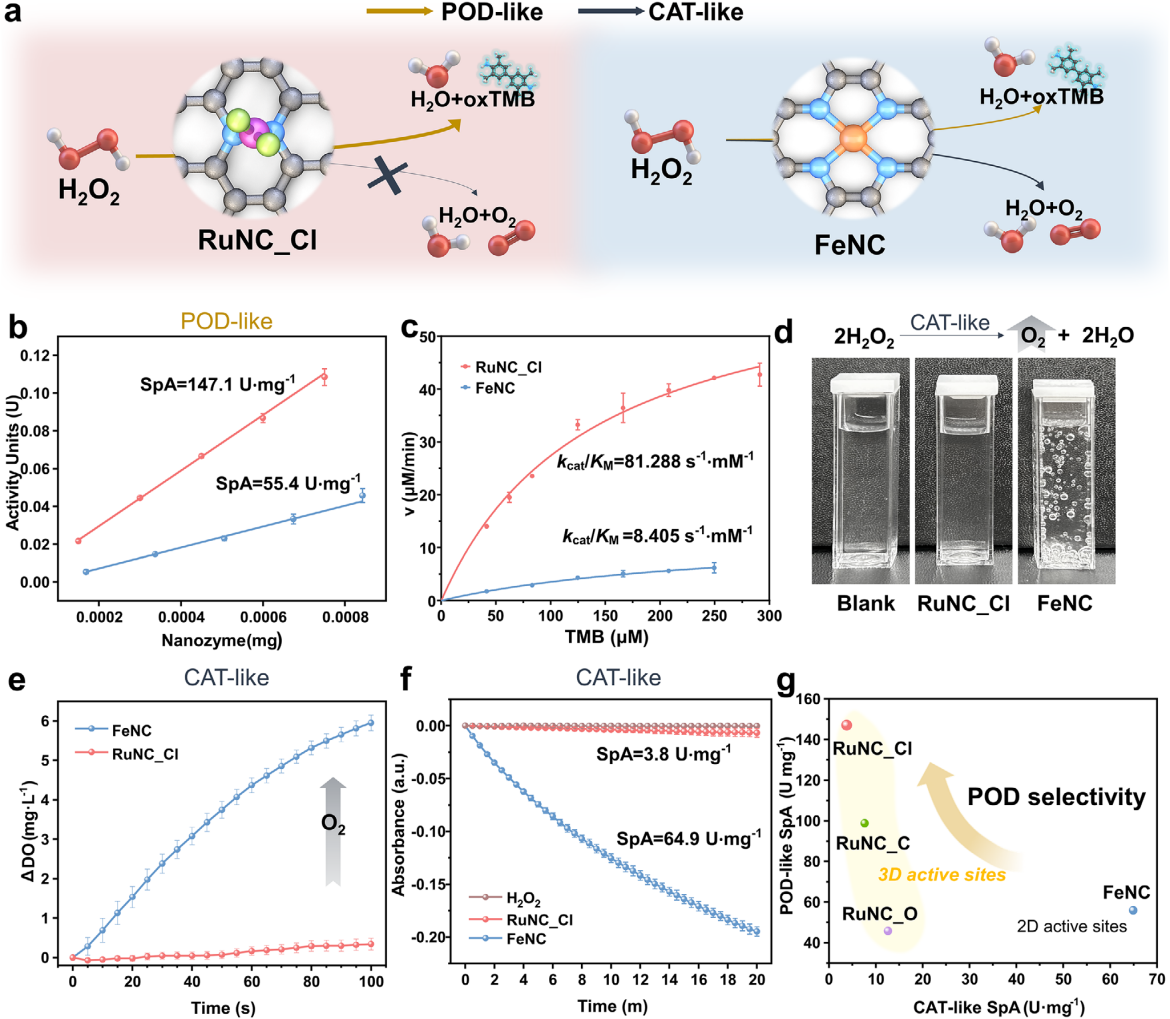
Comparison of Reaction Selectivity of RuNC_Cl and FeNC. a) Schematic illustration regarding the reaction selectivity of RuNC_Cl and FeNC. b) Plot of the POD‐like specific activity of RuNC_Cl and FeNC. c) Steady‐state kinetic assay plots for POD‐like performance using TMB as substrate. d) Images of O_2_ bubbles generated by H_2_O_2_ decomposition under pH 6.0. e) O_2_ generation rate from decomposition of H_2_O_2_ versus time curves. f) H_2_O_2_ decomposition rate versus time curves (relative to *t* = 0). g) Comparison of specific activities for POD‐like and CAT‐like reactions for Ru SAzymes and FeNC.

The CAT‐like reaction, which decomposes H_2_O_2_ under near‐neutral conditions, presents a primary obstacle in POC biosensing. Typical Fe SAzymes with 2D active sites are widely recognized as representative POD‐like nanozymes.^[^ ^49^, ^54^ ^]^ However, these nanozymes have been reported to exhibit high CAT‐like activity owing to the absence of components that regulate reaction pathways.^[^ ^55^ ^]^ Therefore, Fe SAzyme with 2D active sites (FeNC) was selected as the control in this study to assess the role of the surrounding ligands. Unlike FeNC, RuNC_Cl with 3D active sites was expected to exhibit POD‐selective performance (**Figure** **3**a). FeNC was synthesized using ZIF‐8 derived nitrogen‐doped carbon (NC) as a precursor,^[^ ^56^ ^]^ and its morphology and structural characteristics were examined by BET analysis and TEM imaging (Figures S19 and S20, Supporting Information). The atomic dispersion of Fe sites was verified through XRD, HAADF‐STEM, XPS, and XANES analyses (Figures S21—S24, Supporting Information). Furthermore, the formation of 2D‐exposed Fe–N_4_ coordination sites was confirmed by EXAFS fitting (Figure S25, Supporting Information). Detailed description of the synthesized FeNC is provided in Note S3 (Supporting Information).

FeNC demonstrated a POD‐like specific activity of 55.4 U·mg⁻^1^, significantly lower than that of RuNC_Cl (Figure 3b). Furthermore, steady‐state kinetic assay confirmed that RuNC_Cl displayed superior POD‐like performance relative to FeNC. The *k*_cat_/*K*_M_ value of FeNC was 8.405 s^−1^·mm ^−1^, approximately 10 times lower than that of RuNC_Cl for the TMB substrate (Figure 3; Table S6, Supporting Information). Notably, the *k*_cat_ value of FeNC was only 2.22 s^−1^, indicating that the POD‐like reaction proceeded more efficiently at the active sites of RuNC_Cl compared with those of FeNC. Furthermore, the absorbance at 652 nm was evaluated across a range of nanozyme concentrations in the absence of H_2_O_2_ (Figure S26, Supporting Information). FeNC exhibited a concentration‐dependent increase in absorbance, indicating apparent oxidase (OXD)‐like activity. In contrast, RuNC_Cl showed negligible absorbance regardless of its concentration, suggesting minimal intrinsic OXD‐like catalytic behavior. This result underscores the absence of OXD‐like activity for RuNC_Cl, which is crucial for its application as a viable alternative to HRP in biosensors.

The CAT‐like activity of the nanozymes was evaluated by measuring the dissolved oxygen level in the solution and monitoring the decrease in H_2_O_2_ absorption intensity at 240 nm. FeNC generated a large amount of oxygen bubbles, demonstrating strong CAT‐like activity, whereas no visible oxygen bubbles formed within the same timeframe for RuNC_Cl (Figure 3d). Notably, FeNC displayed enhanced CAT‐like activity at higher pH, highlighting the importance of suppressing CAT‐like activity under near‐neutral conditions. In contrast, RuNC_Cl exhibited negligible H_2_O_2_ decomposition regardless of pH (Figure S27, Supporting Information). As shown in Figure 3e, FeNC generated over 4 mg·L⁻^1^ of O_2_ per minute through H_2_O_2_ decomposition, whereas RuNC_Cl exhibited a significantly lower oxygen generation rate. The CAT‐like specific activity, calculated based on H_2_O_2_ decomposition, was significantly higher for FeNC (64.9 U·mg⁻^1^) than that for RuNC_Cl (3.8 U·mg⁻^1^), as shown in Figure 3f. Additionally, the CAT‐like performance was evaluated using a steady‐state kinetic assay. Unlike FeNC, RuNC_Cl deviated from the typical Michaelis–Menten kinetics model, attributable to its inherently low CAT‐like activity (Figure S28 and Table S7, Supporting Information). Furthermore, the H_2_O_2_ decomposition rates for RuNC_C and RuNC_O were below 5% within 20 min, which is significantly lower than that of FeNC (Figure S29, Supporting Information). This indicates that Ru SAzymes exhibited lower CAT‐like performance in contrast to conventional SAzymes. Consequently, compared with FeNC with 2D active sites, Ru SAzymes with 3D active sites exhibited markedly enhanced POD selectivity. In particular, RuNC_Cl demonstrated significantly reduced CAT‐like activity, approximately 38‐fold lower than its POD‐like activity (Figure 3g). These findings confirm that RuNC_Cl could successfully suppress CAT‐like activity, overcoming the bifunctional limitation of FeNC. This selective performance establishes RuNC_Cl as a highly promising candidate for biosensing applications, particularly in near‐neutral pH environments.

### 2.4 Theoretical Evaluation of Reaction Selectivity

Density functional theory (DFT) calculations were conducted to investigate the effects of Ru metal and Cl ligands on the catalytic behavior of RuNC_Cl, with the aim of evaluating its potential for enhanced performance and activity compared with FeNC. Computational structures were constructed based on XANES and EXAFS spectra (Figure 1c and Figure 1e). The DFT‐optimized surface structures of RuNC_Cl and FeNC are provided in the Supporting Information (Figures S30 and S31, Supporting Information). As shown in Figure S1 (Supporting Information), Fe single atoms serve as active sites for both POD‐like and CAT‐like reactions in natural enzymes. Both reaction mechanisms begin with the dissociation of H_2_O_2_, leading to the formation of *O and H_2_O intermediates on the catalyst surface. The *O intermediate plays a pivotal role in determining the catalytic direction. Depending on the reactivity of this intermediate, the catalytic pathways diverge into POD‐like and CAT‐like mechanisms. In the POD‐like reaction, H_2_O_2_ dissociates into *O by releasing H_2_O. The *O intermediate then transitions to *OH through protonation, followed by accepting another proton to form H_2_O. The final step involves the release of this H_2_O molecule, completing the catalytic cycle and regenerating the active site. Conversely, in the CAT‐like reaction progresses differently. After H_2_O_2_ dissociation, the *O intermediate interacts with an additional H_2_O_2_ molecule, resulting in the generation of H_2_O and O_2_.^[^ ^57^, ^58^ ^]^ These reaction pathways were adopted as the basis for the DFT calculations.

**Figure 4:**
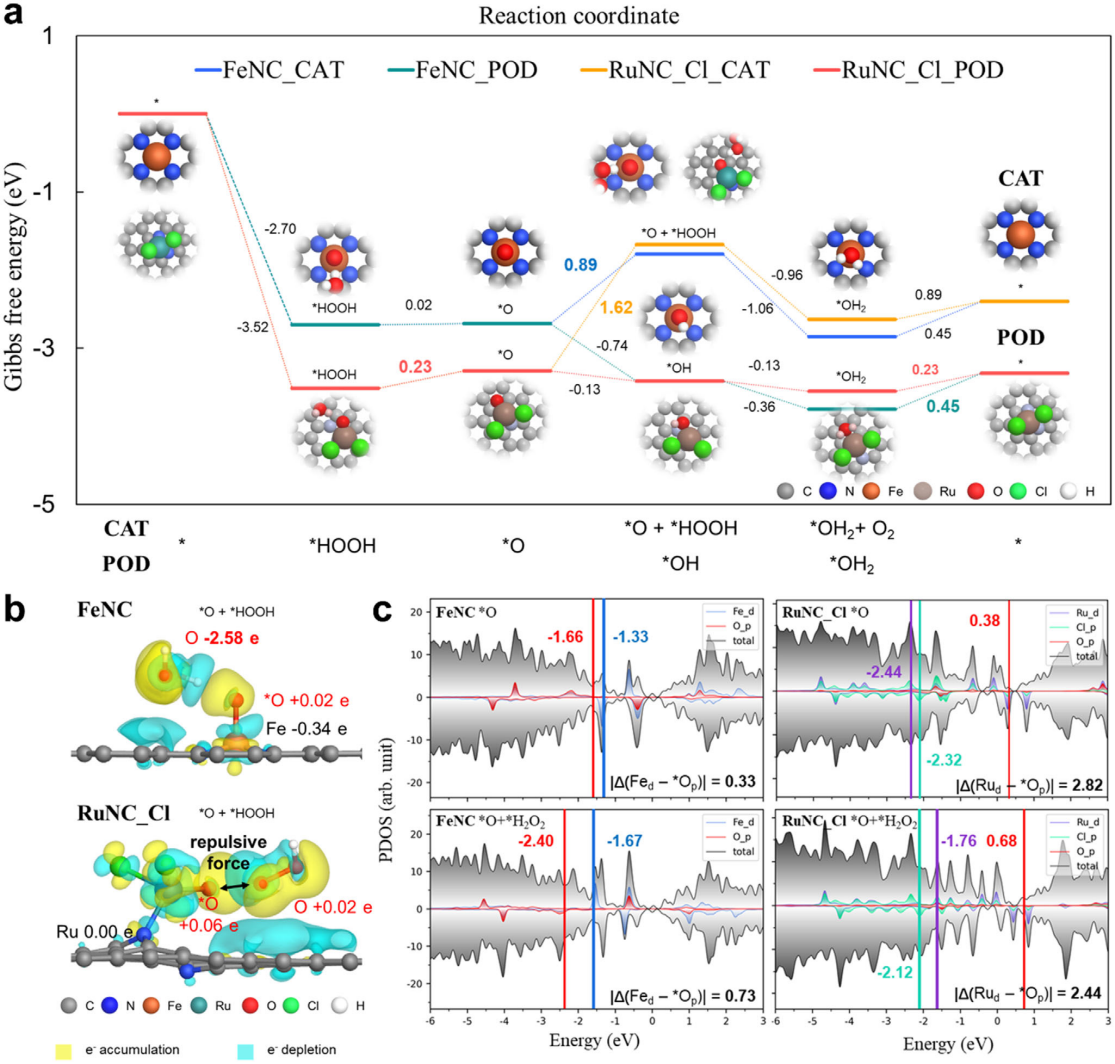
Theoretical evaluation of the POD‐like and CAT‐like activity of the FeNC and RuNC_Cl. a) DFT‐calculated Gibbs free energy diagram of the POD‐like and CAT‐like reaction for the comparison between FeNC and RuNC_Cl. b) Bader charge analysis of the *O + *HOOH intermediate of FeNC and RuNC_Cl. c) Partial density of states for *O and *O + *HOOH intermediates of FeNC and RuNC_Cl with distance between metal d‐band center and oxygen p‐band center.

To evaluate the activity and selectivity of RuNC_Cl, FeNC was selected as the reference. In the case of FeNC, the dissociation of H_2_O_2_ produces an adsorbed *O intermediate at the Fe site, while the remaining H_2_O molecules interact with *O via hydrogen bonding (**Figure** **4**a; Figure S30, Supporting Information). The potential‐determining step (PDS) for the CAT‐like reaction of FeNC (*O → *O + *HOOH) involves the introduction of an additional H_2_O_2_ molecule, which requires an energy barrier of 0.89 eV. In comparison, the PDS for the POD‐like reaction on FeNC, which involves the release of water, *OH_2_ → *, has a lower energy barrier of 0.45 eV. This thermodynamic favorability enables the efficient conversion of H_2_O_2_ to water and protons on FeNC. RuNC_Cl exhibits a distinct catalytic behavior compared with FeNC. In the CAT‐like reaction on RuNC_Cl, the PDS, which involves the introduction of an additional H₂O₂ molecule (*O → *O + *HOOH), requires an energy barrier of 1.62 eV. For the POD‐like reaction, the PDS, associated with water release, has a significantly lower energy barrier of 0.23 eV, comparable with that of FeNC. However, the PDS energy difference between the POD‐like and CAT‐like reactions is considerably larger for RuNC_Cl (1.62 eV – 0.23 eV = 1.39 eV) than that for FeNC (0.89 eV – 0.45 eV = 0.44 eV). This indicates that RuNC_Cl exhibits greater selectivity toward the POD‐like reaction than FeNC. Although the energy barriers for H_2_O release are similar between the two systems, the higher energy requirement for the CAT‐like reaction on RuNC_Cl compared with FeNC (by 0.73 eV) renders it less favorable, thereby promoting the POD‐like reaction pathway. These results demonstrate that the synthesized RuNC_Cl also followed the POD‐like pathway observed in natural enzymes, and preferentially proceeded through the POD‐like mechanism at the *O intermediate.

Ru SAzymes with exposed active sites (RuN_4_ and RuN_2_ models) were examined (Figure S32, Supporting Information) to elucidate the influence of 3D active sites and out‐of‐plane Cl ligands. The Gibbs free energy barriers for the PDS of the CAT‐like reaction (*O → *O + *HOOH) were calculated for these Ru SAzyme models. Interestingly, the energy barrier for RuN_4_ is 0.10 eV, lower than that of FeNC with an FeN_4_ structure. Additionally, the RuN_2_ model exhibits a significantly lower barrier (1.26 eV) compared with that of RuNC_Cl, which contains Cl ligands (1.62 eV). These findings indicate that the presence of Cl ligands greatly impedes H_2_O_2_ approach in CAT‐like reactions, underscoring the critical role of out‐of‐plane ligand coordination when developing POD‐selective nanozymes.

The electronic structure changes upon H_2_O_2_ adsorption provide further insight into enhanced selectivity of RuNC_Cl for the POD‐like reaction. Bader charge analysis revealed that H_2_O_2_ binds more strongly to FeNC compared with RuNC_Cl (Figure 4b; Figure S33, Supporting Information) In the case of FeNC, the oxygen atoms of H_2_O_2_ interacting with the FeNC–*O surface lose approximately 2.58 e, indicating substantial electron sharing. In contrast, for RuNC_Cl, the oxygen atoms lose only 0.02 e, suggesting minimal electron transfer and the presence of repulsive forces. These results can be attributed to the valence orbital occupancy of Ru and the influence of its two Cl ligands.

A partial density of states (PDOS) analysis highlighted another reason underlying the higher selectivity of RuNC_Cl for the POD‐like reaction (Figure 4c). In the case of FeNC, the distance between the *p*‐band center of *O and *d*‐band center of Fe increases from 0.33 eV to 0.73 eV upon H_2_O_2_ adsorption. This weakens charge transfer and reduces the Fe–O bond strength, facilitating H_2_O_2_ adsorption and subsequent reactions. In contrast, in RuNC_Cl, the distance between the *p*‐band center of *O and *d*‐band center of Ru reduces from 2.82 eV to 2.44 eV upon H_2_O_2_ adsorption. This enhanced charge transfer strengthens the Ru–O bond, making additional H_2_O_2_ adsorption less favorable. These findings indicate that the high energy barrier for the CAT‐like reaction on RuNC_Cl, along with its unique electronic structure, contributes to its enhanced selectivity toward the POD‐like reaction.

### 2.5 Colorimetric Detection of Multiple Target Molecules Using RuNC_Cl@µPAD

Small molecules are inherently difficult to detect using antibody‐conjugated assays such as ELISA due to their limited epitope availability. To overcome this limitation in POCT settings, detection systems based on oxidase‐peroxidase cascade reactions have been developed. In this system, precise measurement of the confined amount of H_2_O_2_ generated in situ by the oxidase is crucial for detection sensitivity. However, if the nanozyme also exhibits CAT‐like activity, the limited H_2_O_2_ can be decomposed before it reacts with the chromogenic substrate, resulting in reduced signal intensity and compromised detection sensitivity. High selectivity toward POD‐like activity over CAT‐like activity is a key requirement for reliable small molecule quantification under near‐neutral pH conditions. Leveraging its mesoporous nature and exceptional POD‐like activity, particularly under near‐neutral pH conditions (Figure S16, Supporting Information), RuNC_Cl was selected as the platform for developing colorimetric biosensors in combination with H_2_O_2_‐generating oxidases. This integration enabled a one‐pot cascade reaction for the quantitative detection of various target molecules, such as glucose, lactate, cholesterol, and choline. Notably, the negligible CAT‐like activity of RuNC_Cl prevented H_2_O_2_ degradation, allowing the POD‐driven reaction to proceed efficiently at pH 6.0. The efficiency of the platform was validated by establishing a strong linear correlation between the absorbance and H_2_O_2_ concentration over a range of 0.019–1.25 mm, yielding a limit of detection (LOD) of 1.8 µm (Figure S34a, Supporting Information). In contrast, FeNC, which exhibits high CAT‐like activity, showed inferior performance compared to RuNC_Cl, with a much narrower linear range (0.019–0.156 mm) and higher LOD of 8.7 µm (Figure S34b, Supporting Information). This performance underscores the potential of the RuNC_Cl‐based system for detecting H_2_O_2_ and other analytes involved in its generation, especially under near‐neutral pH conditions (Table S8, Supporting Information).

To further evaluate the biosensing capabilities, glucose oxidase (GOx), used as a model enzyme, was immobilized into various scaffolds including Ru SAzymes (RuNC_Cl, RuNC_C, and RuNC_O), FeNC, and N‐MSUFC using the crosslinked enzyme aggregate (CLEA) method.^[^ ^59^, ^60^ ^]^ Among the tested scaffolds, the mesoporous architectures of Ru SAzymes and N‐MSUFC facilitated a high enzyme‐loading capacity (∼15%), whereas FeNC, lacking mesopores, showed significantly lower loading efficiency (Figure S35, Supporting Information). The detailed characterization supporting the successful GOx immobilization are presented in Figure S36 and Note S4 (Supporting Information). In glucose detection, RuNC_Cl produced the most significant color change upon exposure to 5 mm of glucose, attributable to its superior POD‐like activity. In contrast, N‐MSUFC exhibited minimal activity owing to the absence of intrinsic POD‐like properties (Figure S37a, Supporting Information). Additionally, GOx@RuNC_Cl outperformed GOx@FeNC in glucose detection, owing to enhanced enzyme entrapment (Figure S38 and Video S1, Supporting Information). Consequently, GOx@RuNC_Cl achieved a lower LOD of 2.51 µm compared with 17.0 µm for GOx@FeNC (Table S8, Supporting Information). Collectively, these results establish RuNC_Cl as the optimal scaffold for further enzyme immobilization experiments aimed at detecting multiple analytes through an effective one‐spot cascade reaction.

Building on these findings, four key enzymes, GOx, lactate oxidase (LactOx), choline oxidase (ChOx), and cholesterol oxidase (COx), were successfully immobilized within the pores of RuNC_Cl using the CLEA method. This process yielded high‐enzyme‐loading composites, such as GOx@RuNC_Cl, LactOx@RuNC_Cl, ChOx@RuNC_Cl, and COx@RuNC_Cl, with loading percentages of 16.9%, 12.7%, 16.8%, and 12.1%, respectively (Figure S39, Supporting Information). Optimal pH condition for the cascade reactions to simultaneously detecting four different analytes (glucose, lactate, choline, and cholesterol) was found to be pH 6 (Figure S40, Supporting Information). Consequently, when employed in a colorimetric off‐device assay, these composites enabled the detection of glucose, lactate, cholesterol, and choline, with increasing concentrations of each analyte producing proportional absorbance changes (Figures S38a and S41, Supporting Information). Under optimized conditions, the dynamic linear ranges yielded LODs of 2.5 µm for glucose, 8.8 µm for lactate, 3.6 µm for cholesterol, and 10.1 µm for choline, demonstrating performance comparable with other SAzyme‐based biosensors (Figures S38a and S41, and Table S8, Supporting Information).

To further demonstrate practical applicability in real‐world settings, we additionally constructed RuNC_Cl‐based well‐plate assay kits to quantitatively determine glucose, lactate, cholesterol, and choline in spiked human serum samples. Initial concentrations of these biomarkers in serum were first determined using commercialized assay kits, followed by spiking with predetermined amounts of each target analyte. The RuNC_Cl‐based well‐plate platforms enabled successful determination of all target analytes, yielding sufficient coefficient of variation (CV) and recovery values, ranging 3.1%∼5.4% and 99.4%∼102.8%, respectively, comparable to those obtained with commercialized assay kits (Tables S9 and S10, Supporting Information). Furthermore, the estimated production cost of the RuNC_Cl‐based assay platforms was much lower than those of commercialized kits (Table S11, Supporting Information). These results confirm vivid potential of RuNC_Cl‐based assay kits as reliable and cost‐effective tools for biomarker quantification in POC settings.

**Figure 5:**
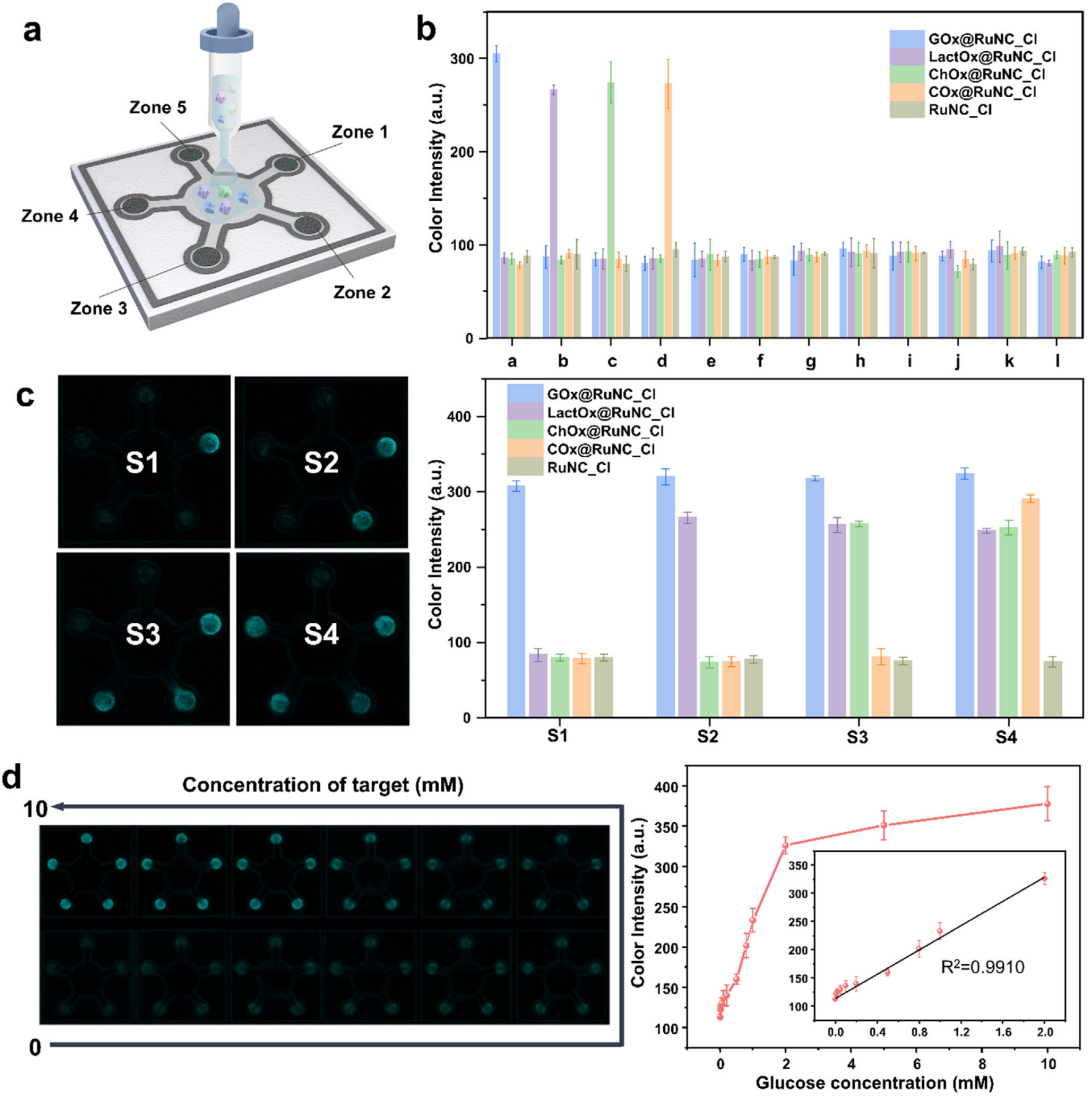
Biomarker quantification with paper‐based microfluidic sensors using RuNC_Cl. a) Illustration of the RuNC_Cl‐based paper microfluidic devices (RuNC_Cl@µPAD) for multiple target detection in which GOx@RuNC_Cl (zone 1), LactOx@RuNC_Cl (zone 2), ChOx@RuNC_Cl (zone 3), COx@RuNC_Cl (zone 4), and RuNC_Cl (zone 5). b) Specificity of the RuNC_Cl@µPAD for the detection of single analyte (a: glucose, b: lactate, c: cholesterol, d: choline, e: galactose, f: BSA, g: cysteine, h: ascorbic acid, i: uric acid, j: Na^+^, k: Ca^2+^, l: buffer). c) Specificity of the device for the detection of multiple biomarkers: solution 1 (glucose), solution 2 (glucose + lactate), solution 3 (glucose + lactate + cholesterol), and solution 4 (glucose + lactate + cholesterol + choline). d) The dose‐response curve and real images of the glucose detection using the GOx@RuNC_Cl‐based paper device. In this paper device, all five detection zones were impregnated with GOx@RuNC_Cl.

To develop practical biosensors for multi‐target detection in POC testing, the enzyme@RuNC_Cl composites were integrated into paper microfluidic devices (RuNC_Cl@µPAD). These devices are particularly well‐suited for POC testing owing to their simple fabrication, minimal sample requirements, portability, and user‐friendly operation. Each device featured a sample zone connected to five detection zones, each impregnated with a TMB substrate and the specific enzyme@RuNC_Cl composite, enabling the simultaneous detection of multiple analytes including glucose, lactate, cholesterol, and choline (**Figure** **5**a). Upon the addition of approximately 40 µL of sample, blue color signals developed rapidly within 5 min (Video S2, Supporting Information), with quantification after 15 min performed using smartphone‐captured images analyzed via ImageJ software (Figure S42, Supporting Information).

Optimizing the fabrication process was critical to prevent reagent leakage. We determined that wax melting at 150 °C for 90 s ensured complete wax penetration, leading to the formation of stable hydrophobic channels (Figure S43, Supporting Information). Under these optimized conditions, the selectivity of RuNC_Cl@µPAD was rigorously evaluated against target molecules (glucose, lactate, cholesterol, and choline) and potential blood interferences (galactose, bovine serum albumin (BSA), cysteine, ascorbic acid, uric acid, and various ions). As demonstrated in Figure 5b, each target was detected exclusively in its designated zone, even in the presence of interferences at ten times higher than that of the target. Moreover, when analyzing mixtures of targets, the proposed device accurately distinguished and quantified each analyte, demonstrating its utility for multi‐biomarker detection in physiological fluids (Figure 5c and Figure S44, Supporting Information). The color intensities increased proportionally with the biomarker concentrations, as confirmed by dose–response curves, with LODs of 6.0 µm for glucose, 9.8 µm for lactate, 10.7 µm for cholesterol, and 5.8 µm for choline. The dynamic linear ranges were 0.01–2.0 mm for both glucose and choline, 0.01–1.0 mm for lactate, and 0.02–1.0 mm for cholesterol, all exhibiting strong linearity (*R* ^2^ > 0.99) (Figure 5d, and Figure S45, Supporting Information). These performance metrics are comparable to those reported for existing paper‐based devices (Table S12, Supporting Information). Additionally, the RuNC_Cl@µPAD devices maintained full functionality for over one month under various storage conditions (Figure S46, Supporting Information), further underscoring their practical utility.

To fully demonstrate the practical applicability of the RuNC_Cl@µPAD devices, they were employed to simultaneously quantify glucose, lactate, cholesterol, and choline in spiked human serum samples. The baseline concentrations of these biomarkers in the serum were first determined using commercial assay kits, followed by spiking with known amounts of each target. The RuNC_Cl@µPAD devices exhibited high precision in quantifying the target levels, with recovery rates between 99.3% and 102.4% and CV values between 2.6% and 11.6% (Table S13, Supporting Information). The high detection accuracy was achieved through a one‐pot cascade reaction without requiring a buffer change. These results confirm the potential of RuNC_Cl@µPAD as reliable tools for multiplexed biomarker quantification in POC settings.

## 3 Conclusion

In this study, Ru SAzymes with remarkably high POD‐selective activity were developed, overcoming the limitations of conventional Fe SAzymes. The combination of N‐MSUFC and low‐temperature hydrogen annealing enabled the formation of out‐of‐plane ligand coordination in Ru active centers. These atomically dispersed 3D active sites exhibited monofunctional POD‐like activity under near‐neutral conditions, and the rational selection of ligand enabled the maximization of Ru utilization. Notably, RuNC_Cl demonstrated a high specific activity for POD‐like reactions, surpassing its CAT‐like activity by 38‐fold. DFT calculations revealed that the CAT‐like reaction on RuNC_Cl required a significantly higher energy barrier (1.62 eV) than that of the POD‐like reaction (0.23 eV), rendering the POD‐like pathway more favorable. Bader charge analysis further confirmed that the presence of Cl ligands hindered the adsorption of additional H_2_O_2_, which is essential for the CAT‐like pathway. These findings revealed that the strategic incorporation of ligands, such as Cl, can effectively regulate reaction pathways in nanozymes. The POD‐selective performance of RuNC_Cl facilitated precise multi‐biomarker detection under near‐neutral conditions. In particular, RuNC_Cl@µPAD accurately quantified glucose, lactate, cholesterol, and choline in spiked human serum samples, exhibiting high recovery rates and reproducibility, thereby establishing its reliability for POC testing. This breakthrough can enhance the practicality of nanozymes for biosensing and provide a rational design strategy for controlling the reaction selectivity.

## 4 Experimental Section

### Materials

Absolute ethyl alcohol was from Fisher. Hydrofluoric acid (HF, 48.0–51.0%) and tetrahydrofuran (THF, 99.9%) were bought from JT baker. Aluminum(III) chloride hexahydrate (AlCl_3_·6H_2_O, 98%) was purchased from Kanto Chemical Co. Dimethyl sulfoxide (DMSO,>99.8%), 3,3′,5,5′‐Tetramethylbenzidine (TMB, 98%) were from TCI. Furfuryl alcohol (98%), Triethylene glycol dimethyl ether (TEGDME, 99%), Urea (99%), Ruthenium(III) chloride hydrate (RuCl_3_·3H_2_O), Ruthenium(III) acetylacetonate (Ru(acac)_3_), Zinc nitrate hexahydrate (Zn(NO_3_)_2_·6H_2_O), 2‐methylimidazole, iron chloride (FeCl_3_), hydrogen peroxide (30 wt% in H_2_O), glutaraldehyde (25%, w/v), glucose oxidase from *Aspergillus niger* (GOx), lactate oxidase from *Aerococcus viridans* (LactOx), cholesterol oxidase from *Streptomyces* sp. (ChOx), choline oxidase from *Arthrobacter globiformis* (COx), glucose, lactate, cholesterol, choline chloride, galactose, bovine serum albumin (BSA), cysteine, ascorbic acid, uric acid, sodium chloride, calcium chloride, human serum were all purchased from Sigma Aldrich. Bis(2‐methylallyl)(1,5‐cyclooctadiene)ruthenium(II) was obtained from Strem. All the chemicals were used without further purification and all aqueous solutions were prepared using deionized water (18.2 MΩcm^−1^) from a Milli‐Q Academic system.

### Synthesis of Nitrogen Doped Mesocellular Carbon Foam (N‐MSUFC)

The synthesis procedure of Mesocellular carbon foam (MSUFC) followed the previous report.^[^ ^61^ ^]^ MSUFC was prepared via hard template method and mesocellular aluminosilicate foam (MSU‐F‐Si) was used as silica template. First, MSU‐F‐Si was mixed with AlCl_3_·6H_2_O in ethyl alcohol and keep stirred to evaporate solvent at 80 °C. After mixing furfuryl alcohol and TEGDME in a 1:1 ratio, the mixture was impregnated into the pores of aluminated MSU‐F‐Si. The polymerization was conducted at 85 °C for 8 h under vacuum and the resultant materials were carbonized at 850 °C for 2 h under Ar. The remaining MSU‐F‐Si was etched by HF solution. To enhance the affinity of metal precursors on support, nitrogen was doped onto MSUFC via soft nitriding method. 1 g of MSUFC and 1.5 g of urea were physically grinded using mortar. The mixture was heated at 150 °C for 2 h and then 300 °C for 2 h under air, followed by washing with ethanol. The resulting powder was vacuum dried at 60 °C for 12 h to obtain N‐MSUFC.

### Synthesis of Ru SAzymes

Ru SAzymes were prepared by the wet impregnation method. For RuNC_Cl, 200 mg of N‐MSUFC was well dispersed in 20 mL of ethyl alcohol (or THF) and a solution of the RuCl_3_·3H_2_O (5 mg mL^−1^) was added dropwise to the carbon support under stirring. The mixture was dried at 50 °C under continuous stirring and transferred into a vacuum oven at 60 °C for 12 h. The obtained powder was placed in crucible and heated at 200 °C for 2 h under 10% H_2_/Ar. The synthesis of RuNC_C and RuNC_O was similar to that of RuNC_Cl, except that the ruthenium precursor was replaced by Bis(2‐methylallyl)(1,5‐cyclooctadiene)ruthenium and Ru(acac)_3_, respectively.

### Synthesis of FeNC

The synthesis of FeNC followed the previously reported method involving ion adsorption and heat treatment on ZIF‐8 derived NC.^[^ ^40^ ^]^ Initially, for a fabrication of typical ZIF‐8 metal‐organic framework, Zn(NO_3_)_2_·6H_2_O (3.39 g) was dissolved in methanol (300 mL) with sonication in flask A. 2‐methylimidazole (3.94 g) was dissolved in methanol (300 mL) with sonication in flask B. Then, the solution in flask B was poured into flask A and mixed at 60 °C for 24 h. This colloidal solution was centrifuged and washed with methanol and vacuum dried at 60 °C for 12 h. Subsequently, the resulting ZIF‐8 powder was annealed at 1100 °C for 2 h under Ar to obtain NC. Afterward, the NC was dispersed in isopropanol (10 mg mL^−1^) with iron chloride. The solution was stirred vigorously for 24 h and centrifuged. The obtained powder was vacuum dried and heated at 700 °C for 1 h under Ar.

### Characterization

To investigate the mesoporous structure of MSUFC, Brunauer‐Emmett‐Teller (BET) specific surface areas of the catalysts were measured by nitrogen physisorption analysis at 77 K using a Micromeritics Tristar II 3020 system. The transmission electron microscopy (TEM; G2 F30 S‐Twin, Tencai) and the scanning electron microscopy (SEM; Hitachi S‐4200) were used to observe the structure of Ru SAzmes. XRD patterns were obtained using a RIGAKU D/MAX‐2500 V X‐ray diffractometer (Cu Kα radiation, λ = 1.541 Å). HAADF‐STEM and EDS (Titan cubed G2 60–300) was used to determine the atomically dispersed metal states. The XPS data were collected using a VG Scientific Escalab 250 (Al Kα). The coordination of Ru SAzymes was characterized using XAS beamline at Pohang Accelerator Laboratory (PAL, Korea) and further processed by Athena software. Wavelet transform (WT) of the EXAFS data was carried out using JWT‐EXAFS 1.0.0 software. The ruthenium contents were measured by inductively coupled plasma mass spectrometer (ICP‐MS; Agilent ICP‐MS 7700S) and inductively coupled plasma optical emission spectroscopy (ICP‐OES; Agilent ICP‐OES 5110). The catalytic activity of POD, OXD and CAT was determined using UV–vis spectrophotometer (UV‐1900i, SHIMADZU). The O_2_ generation was monitored using a dissolved oxygen meter (DO‐31P, DKK‐TOA).

### Catalytic Activity Test—POD‐Like Activity

The POD‐like activity was measured according to reported protocols.^[^ ^28^ ^]^ The TMB was used as substrate for the colorimetric detection due to the unique absorption peak at 652 nm of oxidized TMB. In this system, the amount of nanozyme that catalyzed 1 µmol of TMB per minute was defined as 1U. First, Ru SAzymes were dispersed with ultra‐sonication (35%, 30 min) in phosphate buffer (pH 6.0, 10 ug mL^−1^). Then, TMB solution in DMSO (10 mg mL^−1^) and H_2_O_2_ solution (11.63 M) were prepared. The measurement was conducted with mixing three solutions with phosphate buffer (pH 6.0) varying the amounts of Ru SAzymes solutions in a cuvette. The oxidation of TMB by POD reaction could be confirmed by the increase in absorbance at 652 nm. The catalytic activity unit (U) was calculated by the following formula.

(1) $$Catalytic\;activity\left(U\right)=\frac{\Delta A\cdot V_{total}}{\varepsilon \cdot l\cdot \Delta t}$$

In the formula, ∆*A* is the absorbance intensity change at 652 nm and ∆*t* is the reaction time. The extinction coefficient(ε) of TMB at 652 nm was 39000 M^−1^cm^−1^. *V*_total_ is a total solution volume (1 mL) and *l* is the optical path length (1 cm). The specific activity (SpA) were determined by the slope of the plot of catalytic activity versus the amount of Ru SAzymes.

### Catalytic Activity Test—CAT‐Like Activity

The CAT‐like activity of Ru SAzymes and FeNC was determined by UV‐assay using the distinctive absorption peak of H_2_O_2_ at 240 nm.^[^ ^62^ ^]^ The optical intensity at 240 nm decreases as H_2_O_2_ decomposes at CAT reaction. The specific activity of Ru SAzymes was calculated by the following equation.

(2) $$Specific\;Activity\;\left(SpA\right)\left(U\cdot mg^{-1}\right)=\frac{\Delta A\cdot V_{total}}{\varepsilon \cdot l\cdot V_{cat}\cdot C_{cat}\cdot \Delta t}$$

In the equation, the catalytic activity unit(U) was defined as the amount of nanozyme that decomposed µmol of H_2_O_2_ per minute. ∆*A* is the absorbance intensity change at 240 nm and ε is the extinction coefficient of H_2_O_2_ at 240 nm (43.6 M^−1^cm^−1^). The other parameters were same with that of the POD‐like specific activity equation.

### Enzymatic Kinetic Analysis—POD‐Like Performance

The enzyme kinetics for POD‐like performance were evaluated by varying the concentrations of TMB and H_2_O_2_. The reaction progress was monitored by measuring the absorbance changes at 240 nm using UV–vis spectrophotometer. The obtained data were fitted to the Michaelis‐Menten equation using GraphPad Prism version 9 software to determine the kinetic parameters *K*_M_​ and *v*_max_. The *k*_cat_ value was calculated by dividing the *v*_max_ by the molar concentration of ruthenium([E]).

### Enzymatic Kinetic Analysis—CAT‐like performance

For the CAT‐like kinetic assay, the rate of O_2_ generation was compared by varying the concentrations of H_2_O_2_. The kinetic parameters were obtained using the same method as described for the POD‐like kinetic assays.

### Computational Details

All DFT calculations were performed using the Vienna ab initio Simulation Package.^[^ ^63^, ^64^, ^65^ ^]^ The Perdew–Burke– Ernzerhof exchange‐correlation functional^[^ ^66^ ^]^ and projector augmented wave (PAW)^[^ ^67^, ^68^ ^]^ pseudo‐potential were employed with spin‐polarization. Geometric optimization was performed with a total energy convergence criterion of 10⁻^5^ eV, and atomic positions were relaxed until the forces acting on each atom were below 0.03 eV Å^−1^. The plane‐wave cutoff energy was set at 400 eV, and Brillouin‐zone sampling utilized a 3 × 3 × 1 Monkhorst‐Pack grid.^[^ ^69^ ^]^ To minimize interactions between periodic cells, a vacuum layer of approximately 15 Å was introduced between adjacent graphene models.

The Gibbs free energies were calculated by correcting the DFT energy, *E*, with the entropy, *S*, at room temperature, *T* = 298.15 K, and the zero‐point energy, ZPE.

(3) ΔG=ΔE−TΔS+ΔZPE

The computational hydrogen electrode (CHE) model^[^ ^70^ ^]^ was employed to account for the energies of protons (H^+^) and electrons (e^−^). The band centers were determined using the following equation:

(4) $$\frac{\int_{-\infty }^{\infty }E\cdot \rho \left(E\right)dE}{\int_{-\infty }^{\infty }\rho \left(E\right)dE}$$

where *E* is the Fermi level and *𝜌* is the density of states.

### Preparation of Enzyme@RuNC_Cl

The enzyme@RuNC_Cl composites were prepared through the following procedure. RuNC_Cl (5 mg) was mixed with an enzyme solution, GOx (1 mL, 5 mg mL^−1^), LacOx (1 mL, 2 mg mL^−1^), ChOx (1 mL, 3 mg mL^−1^) or COx (1 mL, 2 mg mL^−1^), in a sodium acetate buffer (100 mm, pH 6.0). The mixture was vortexed for 30 s, sonicated for 10 s, and incubated at room temperature (RT) with shaking at 250 rpm for 1 h. Following incubation, the samples were briefly washed with sodium phosphate buffer (100 mm, pH 8.0) and then incubated in an aqueous glutaraldehyde (GA, 0.1% w/w) solution at 200 rpm for 30 min. After GA treatment, the samples were washed twice with phosphate buffer (100 mm, pH 8.0) and then incubated in Tris‐HCl buffer (100 mm, pH 8.0) at 200 rpm for 30 min to quench unreacted aldehyde groups. Finally, the samples were washed once with phosphate buffer (100 mm, pH 8.0), twice with sodium acetate buffer (100 mm, pH 6.0), and stored at 4 °C in sodium acetate buffer (100 mm, pH 6.0) until use. Protein leaching was measured using the bicinchoninic acid (BCA, PIERCE, Rockford, IL) method, and the final enzyme loading in the mesoporous structure was calculated based on the difference between the initially added enzyme and the amount in the leached solution. For comparison, GOx immobilization on control samples such as RuNC_O, RuNC_C, and FeNC was performed using a similar method.

### Design and Preparation of RuNC_Cl@µPAD

The RuNC_Cl@µPAD was created using a wax patterning method. The hydrophobic pattern was designed with AutoCAD and printed onto chromatography filter paper using a wax printer (Xerox ColorQube 8570). The printed paper was heat‐treated at 150 °C for 90 s, followed by natural cooling at RT. This process caused the wax to melt and penetrate through the paper, forming hydrophobic barriers. The RuNC_Cl@µPAD design included a central sample zone (15 mm) and five detection zones (6 mm), labeled 1–5, each containing different enzyme@RuNC_Cl composites and TMB for the detection of glucose, lactate, cholesterol, choline, and control, respectively. These detection zones were connected to the central zone via independent channels (5 mm long and 3 mm wide) to facilitate capillary‐driven flow. The wax barriers were printed to a thickness of 0.7 mm to prevent sample leakage. The final dimensions of the RuNC_Cl@µPAD were 38 mm × 38 mm.

### Preparation of RuNC_Cl@µPAD for Multiple Analytes Detection

To construct RuNC_Cl@µPAD, as‐prepared enzyme@RuNC_Cl suspension (1.2 µL, 1 mg mL^−1^) and TMB (1.2 µL 1 mm) were sequentially spotted onto the detection zones of the paper devices. The devices were then allowed to air‐dry at RT. For simultaneous detection of multiple analytes, 40 µL of sample solution containing glucose, lactate, cholesterol, and choline was added to the central zone and incubated for 20 min at RT. After incubation, images of the µPADs were captured using a smartphone. The images were processed in cyan‐magenta‐yellow‐black (CMYK) mode and analyzed using ImageJ software (NIH) for quantitative assessment.

## Conflict of Interest

The authors declare no conflict of interest.

## Supporting information



Supporting Information

Supplemental Video1

Supplemental Video2

## Data Availability

Research data are not shared.
