# Supplementary material for: Breaking the Selectivity Barrier of Single‐Atom Nanozymes Through Out‐of‐Plane Ligand Coordination
Source: Adv Mater. 2025 Jul 6;37(38):2506480. doi: 10.1002/adma.202506480 (PMC12464650; doi:10.1002/adma.202506480)
Supplement: Supplementary file 1 — Supporting Information [file ADMA-37-2506480-s002.docx]

Supporting Information

Breaking the Selectivity Barrier of Single-Atom Nanozymes through Out-of-plane Ligand Coordination

Seonhye Park**,** Kyu In Shim, Phuong Thy Nguyen, Daeeun Choi, Seongbeen Kim, Seung Yeop Yi, Moon Il Kim,* Jeong Woo Han,* and Jinwoo Lee*


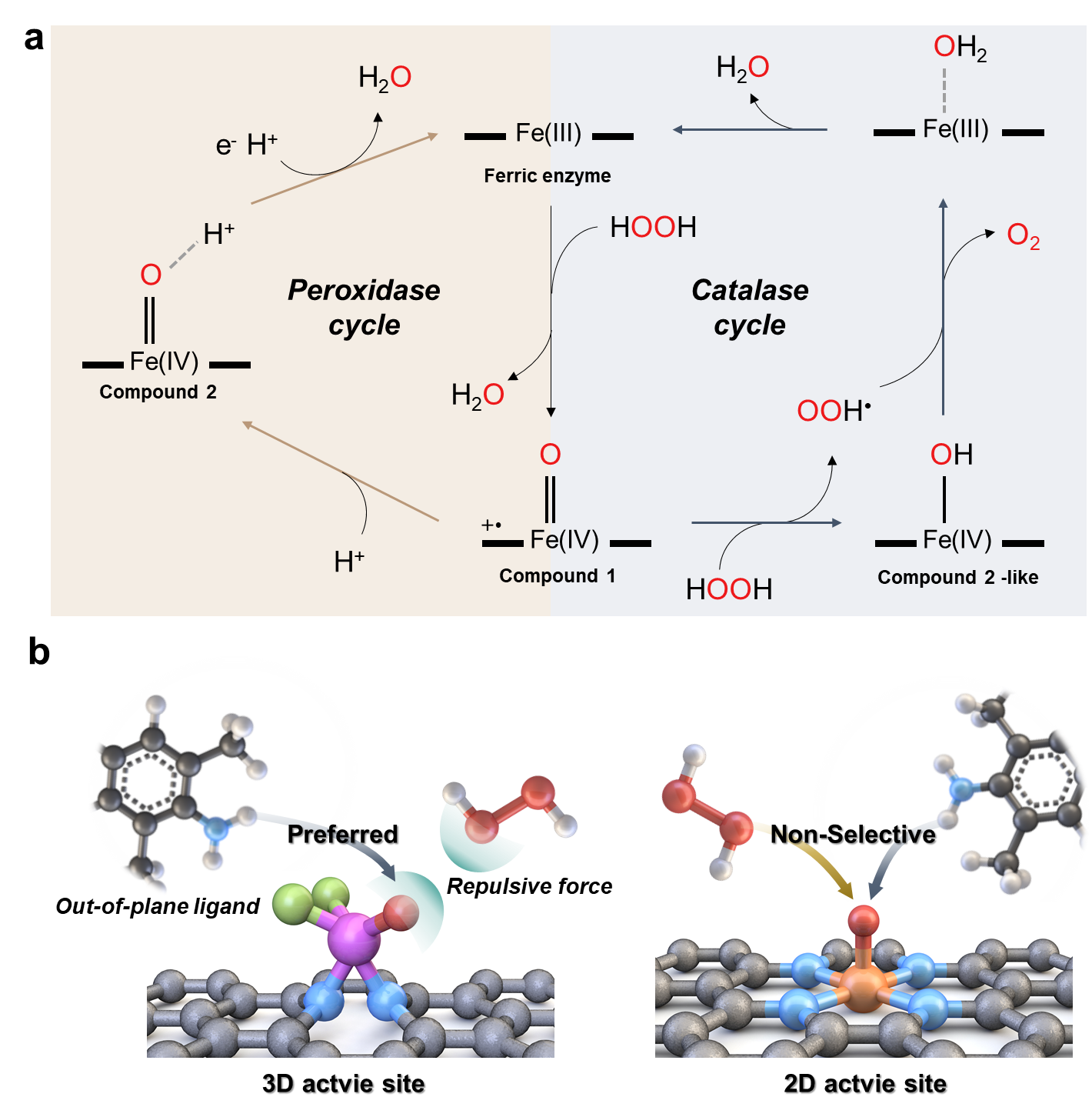


**Figure S1.** Schematic images of a) peroxidase reaction and catalase reaction in natural enzymes b) reactivity of the oxoferryl state at each active site.

**Supplementary Note 1.** Differences between peroxidase reaction and catalase reaction pathways in natural enzymes.

The enzymatic cycles of both heme catalases and peroxidases involve the formation of compound I (Cpd I), where heme is initially oxidized to the oxoferryl state (Fe^IV^=O). While peroxidases preferentially catalyze reduction by electron donating substrates, catalases employ this intermediate to oxidize a second molecule of H_2_O_2_. The reactivity of these intermediates is a critical determinant in directing enzymatic reaction pathways. This suggests that modulating the reactivity of the reaction intermediate—the oxoferryl state—is pivotal for achieving selectivity toward POD-like reactions. The challenge with the previous SAzymes was its exposed two-dimensional active site with no component to control both reactions. To address this, a three-dimensional active site incorporating out-of-plane ligands was introduced, which preferentially promotes the peroxidase reaction. This configuration results in a repulsive force between the reaction intermediate (metal=O) and the oxygen atom of H_2_O_2_, preventing the oxidation of the second molecule of H_2_O_2_.


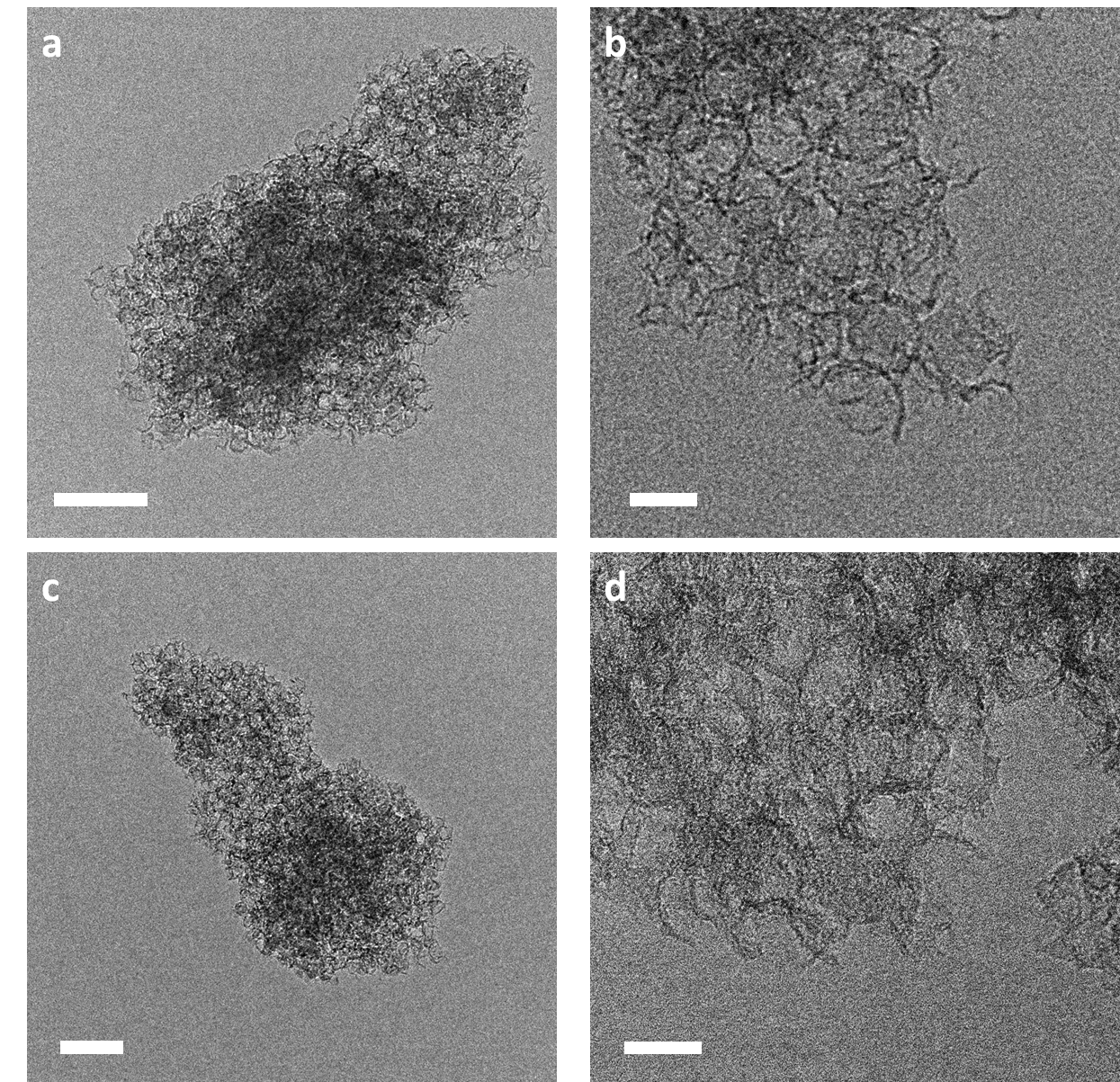


**Figure S2.** Structure characterization of the MSUFC and N-MSUFC.

a) TEM images of MSUFC (scale bar=200 nm) b) (scale bar=20 nm) c) TEM images of N-MSUFC (scale bar=200 nm) d) (scale bar=20 nm).


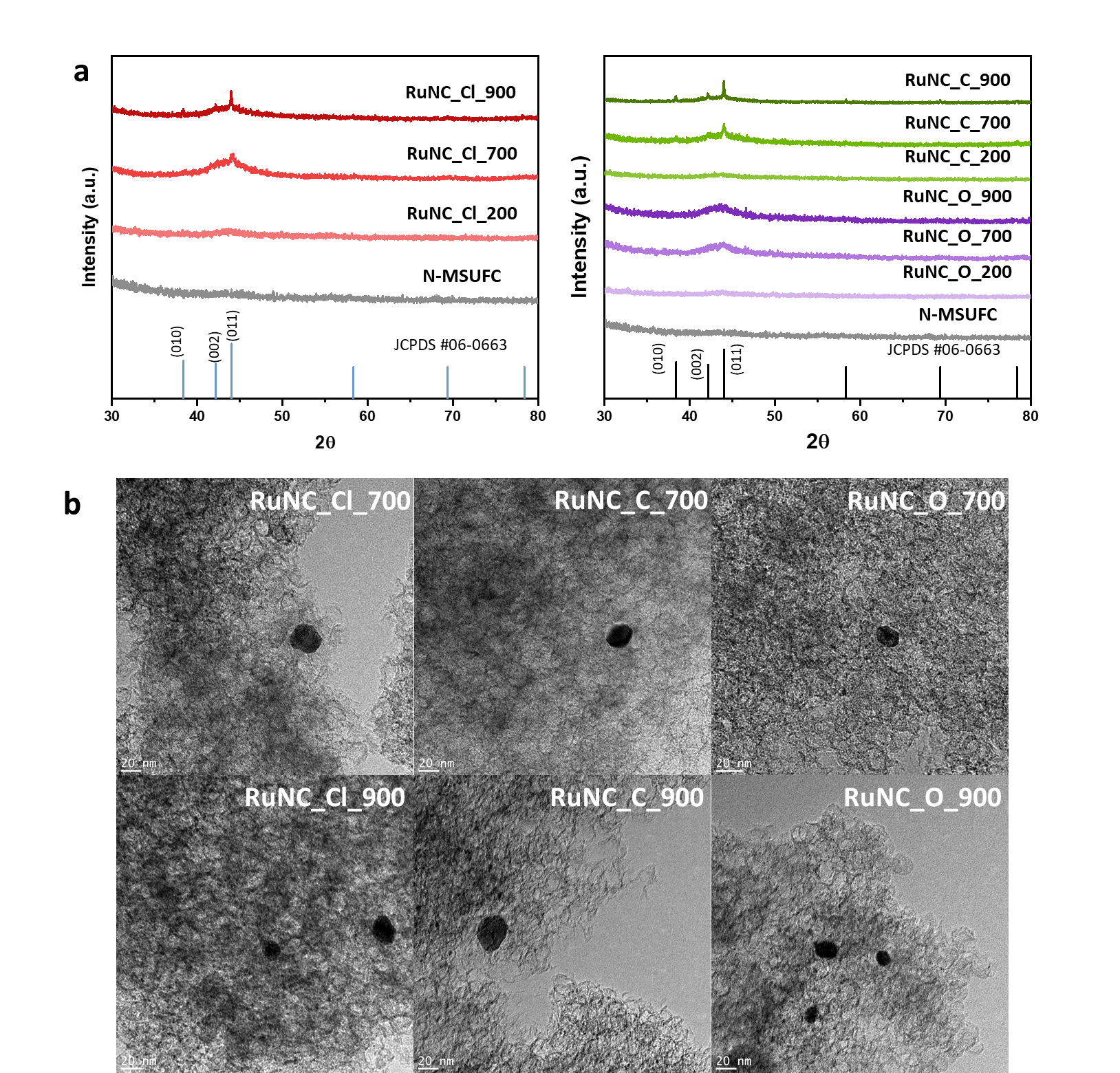


**Figure S3.** Structure characterization of Ru Nanozymes synthesized at high temperature.

a) X-ray diffraction patterns of Ru Nanozymes synthesized at high temperature (JCPDS #06-0663 means hexagonal Ru metal) b) TEM images of Ru nanozymes synthesized at high temperature (scale bar =20 nm).

**Supplementary Note 2**. Ru nanozymes synthesized at high temperature.
As the annealing temperature increased, Ostwald ripening occurred, leading to the formation of a large number of Ru nanoparticles on the surface. To investigate the temperature dependent state of ruthenium, Ru nanozymes annealed at high temperature (700 ℃ and 900 ℃) were also explored. In contrast to Ru SAzymes heat-treated at 200 ℃, Ru nanozymes heat-treated at 700℃ and 900℃ exhibited distinct Ru metal peaks, regardless of the precursor type (**Figure S3 a**). TEM images further confirmed the aggregation of Ru into nanoparticles in these high-temperature samples (Figure S3 b).


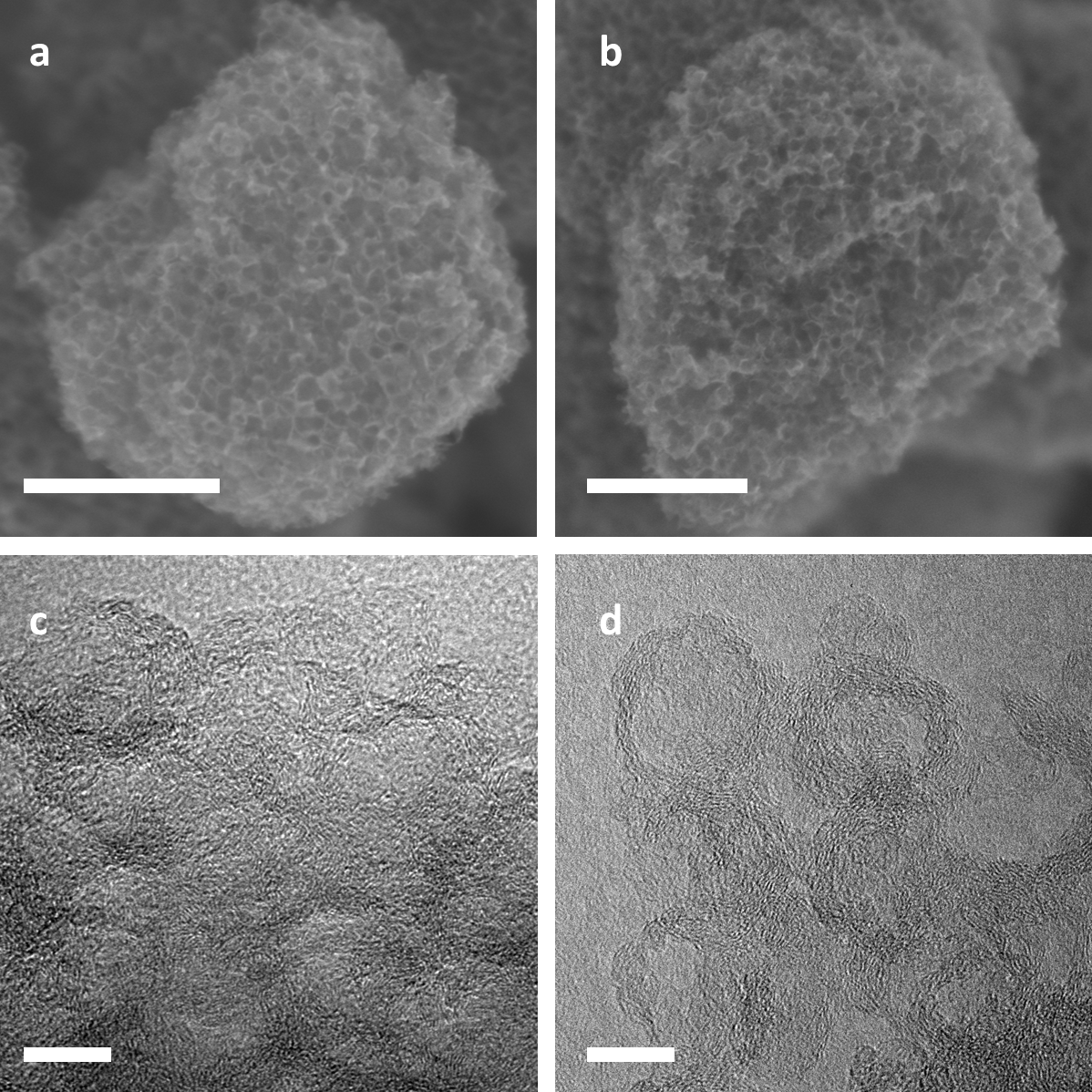


**Figure S4.** Structural characterization of Ru SAzymes.

a) SEM images of RuNC_C (scale bar =200 nm) b) SEM images of RuNC_O (scale bar =200 nm) c) TEM images of RuNC_C (scale bar =10 nm) d) TEM images of RuNC_O (scale bar =10 nm).


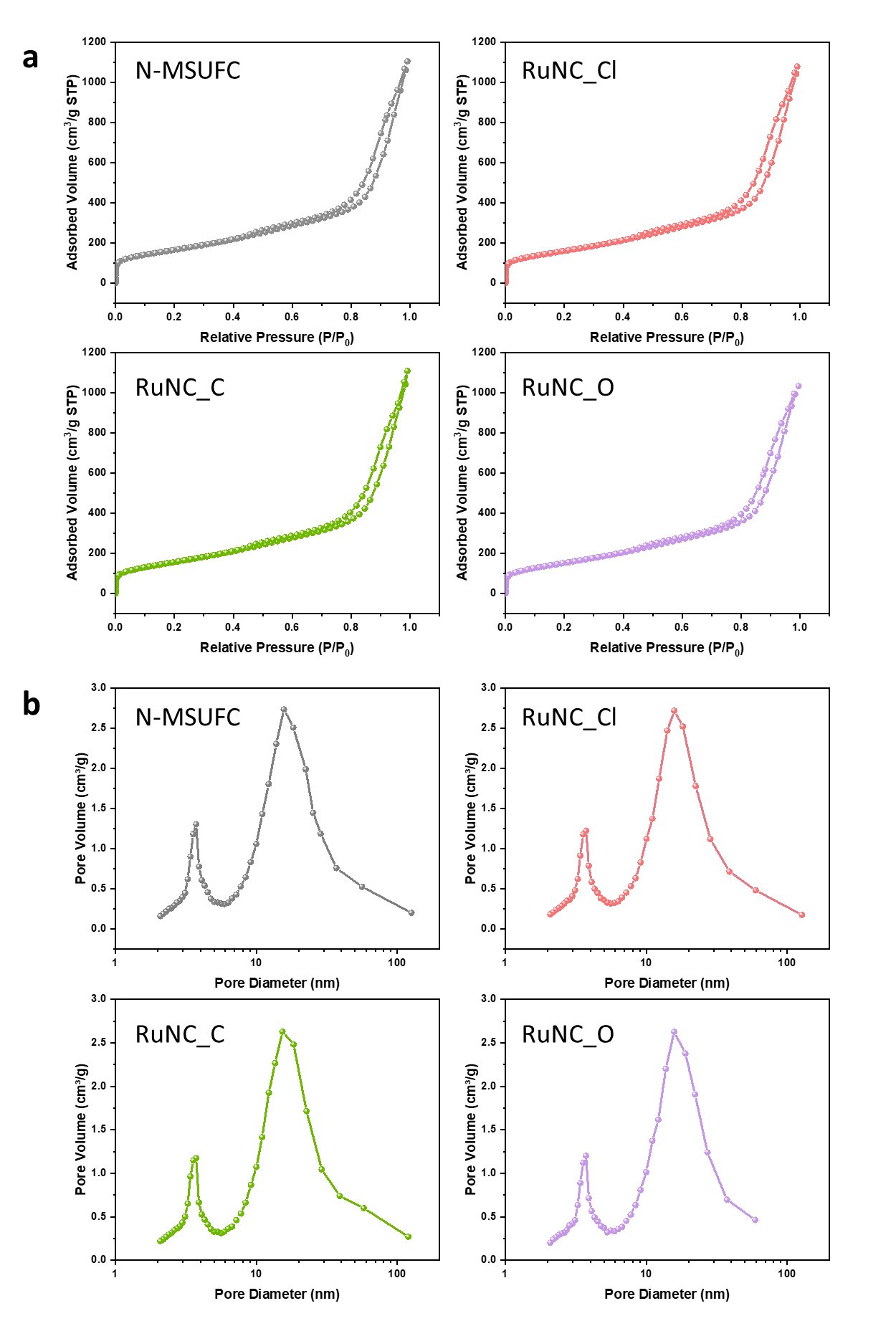


**Figure S5.** BET analysis of N-MSUFC and Ru SAzymes.

a) Nitrogen adsorption-desorption isotherms of N-MSUFC and Ru SAzymes b) Pore size distributions of N-MSUFC and Ru SAzymes.

**Figure S6.** Size distribution profile using Dynamic Light Scattering of Ru SAzymes.


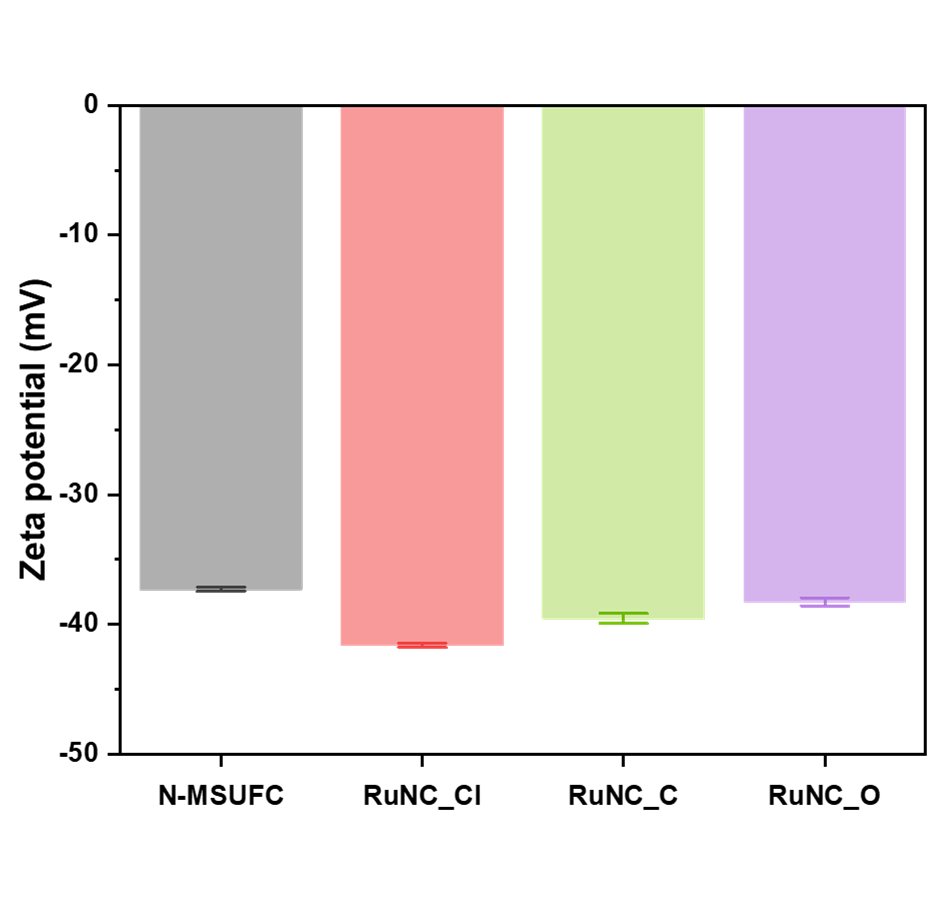


**Figure S7.** Zeta potential measurements of N-MSUFC and Ru SAzymes


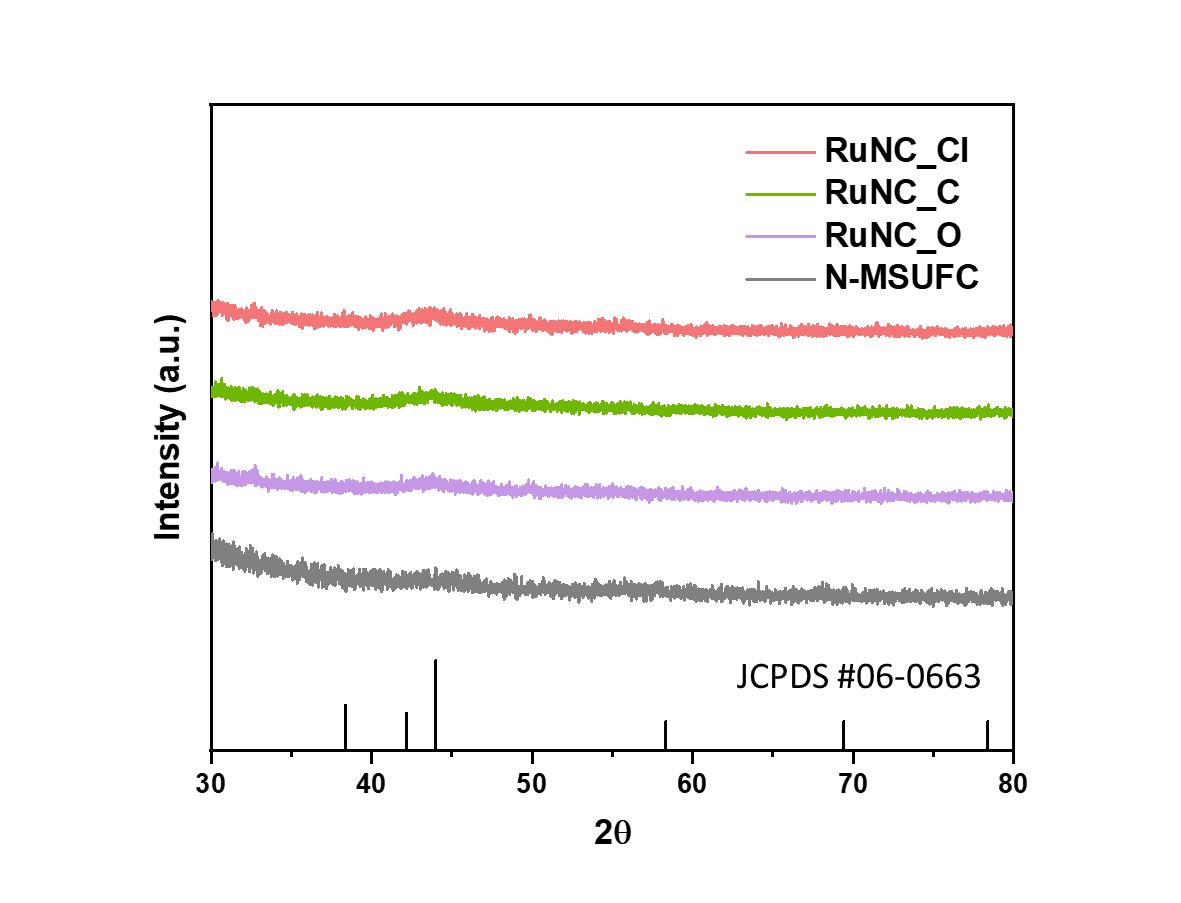


**Figure S8.** X-ray diffraction patterns of Ru SAzymes (JCPDS #06-0663 means hexagonal Ru metal).


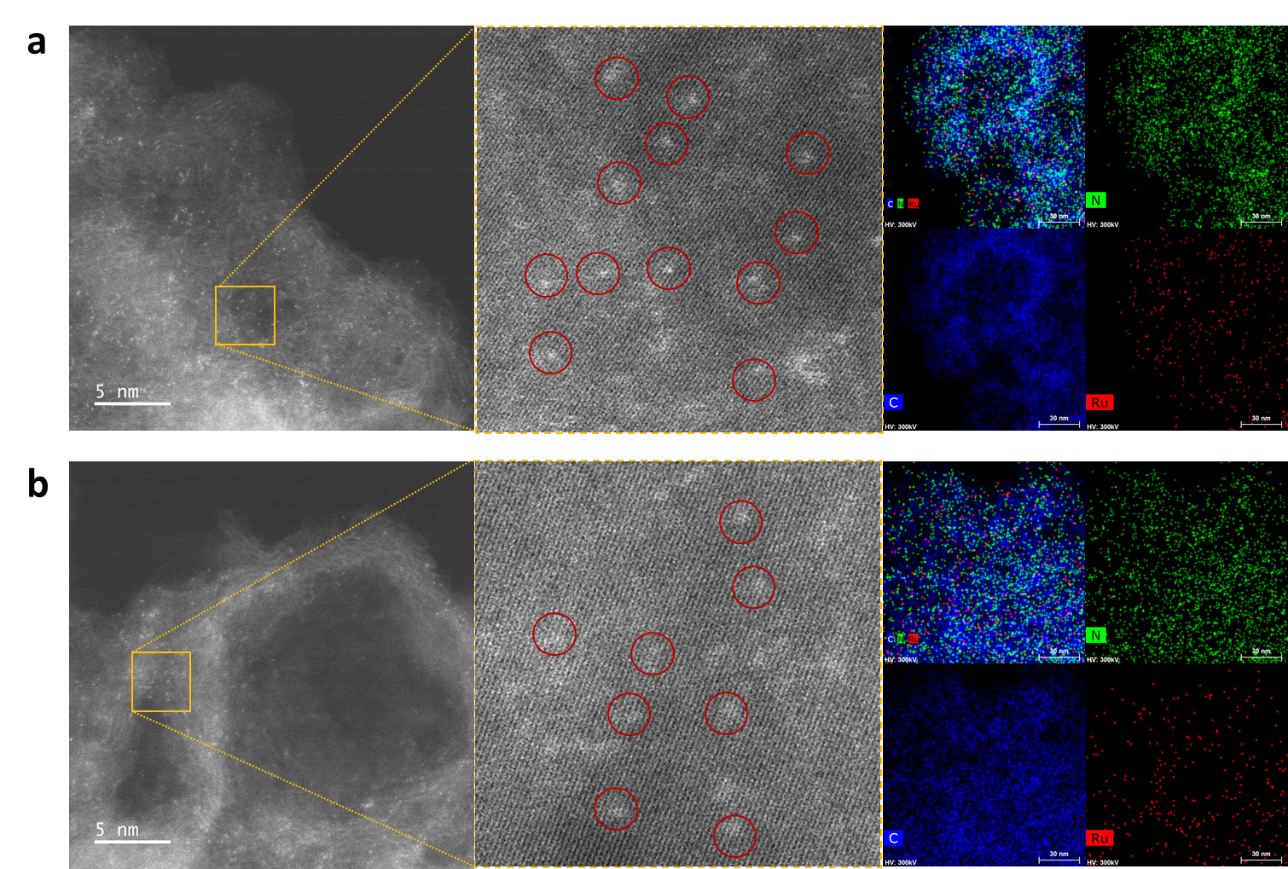


**Figure S9.** HAADF-STEM images and Energy-dispersive X-ray elemental (EDX) mappings of Ru SAzymes.

a) RuNC_C b) RuNC_O (scale bar =5 nm).


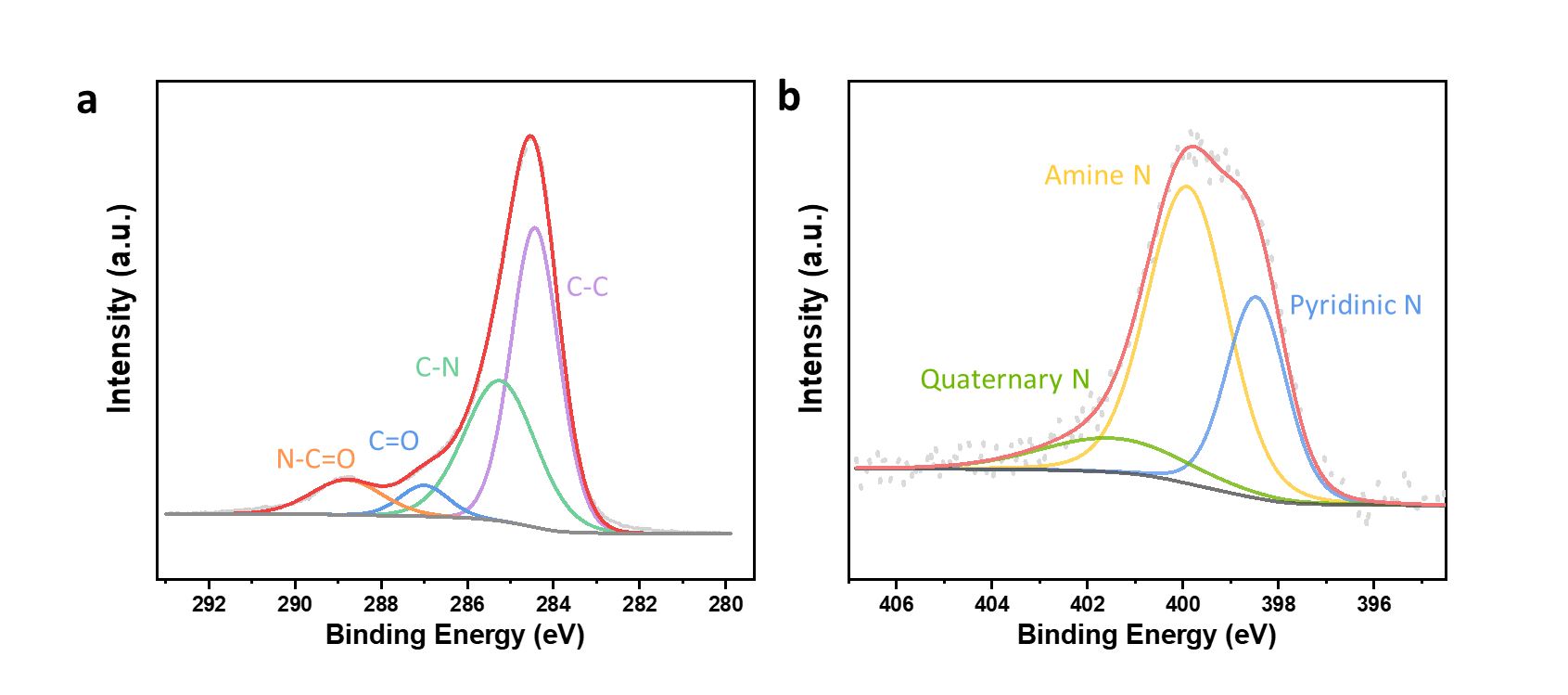


**Figure S10.** XPS spectrum of N-MSUFC.

a) C 1s spectra b) N 1s spectra.


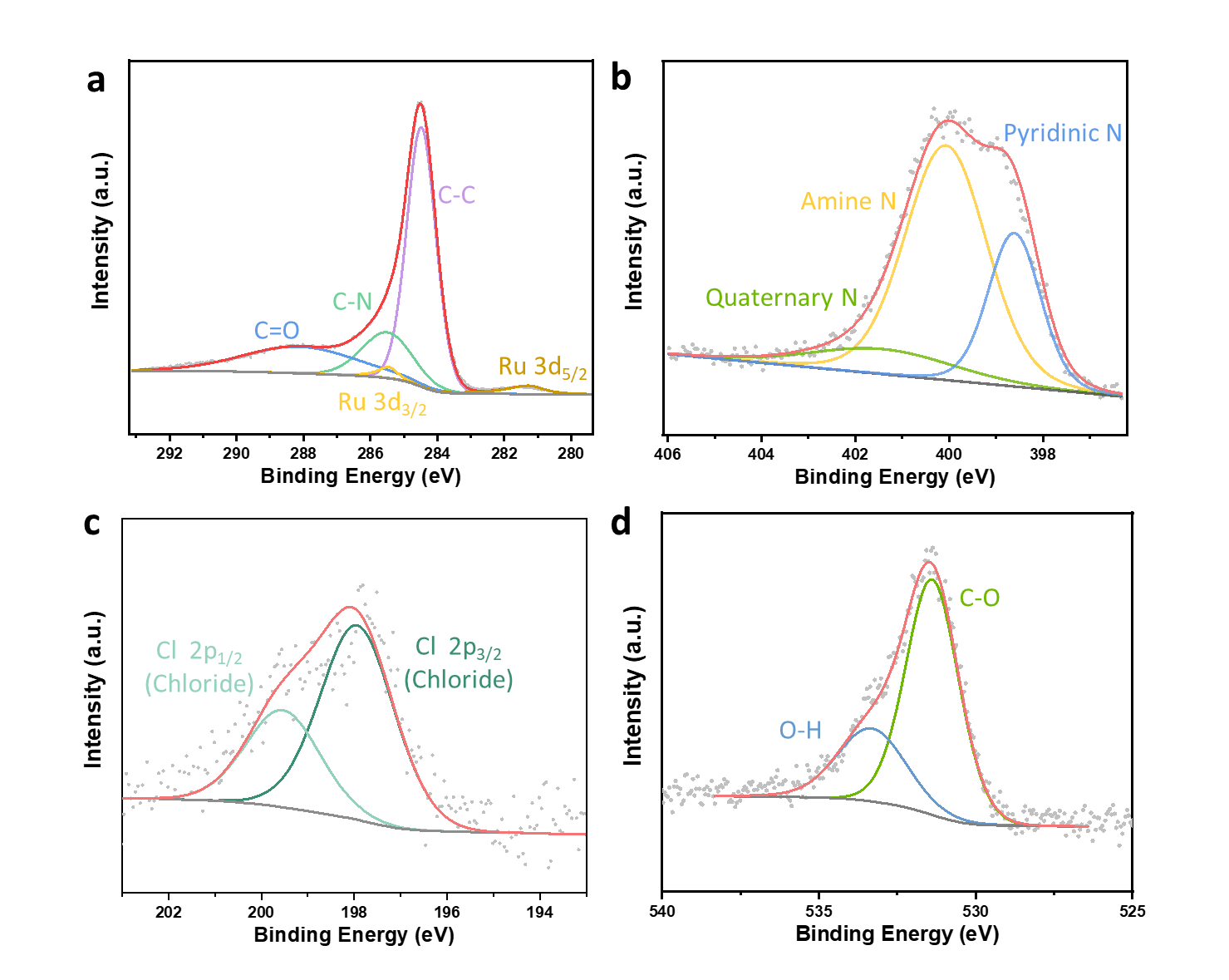


**Figure S11.** XPS spectrum of RuNC_Cl.

a) C 1s spectra b) N 1s spectra c) Cl 2p spectra d) O 1s spectra.


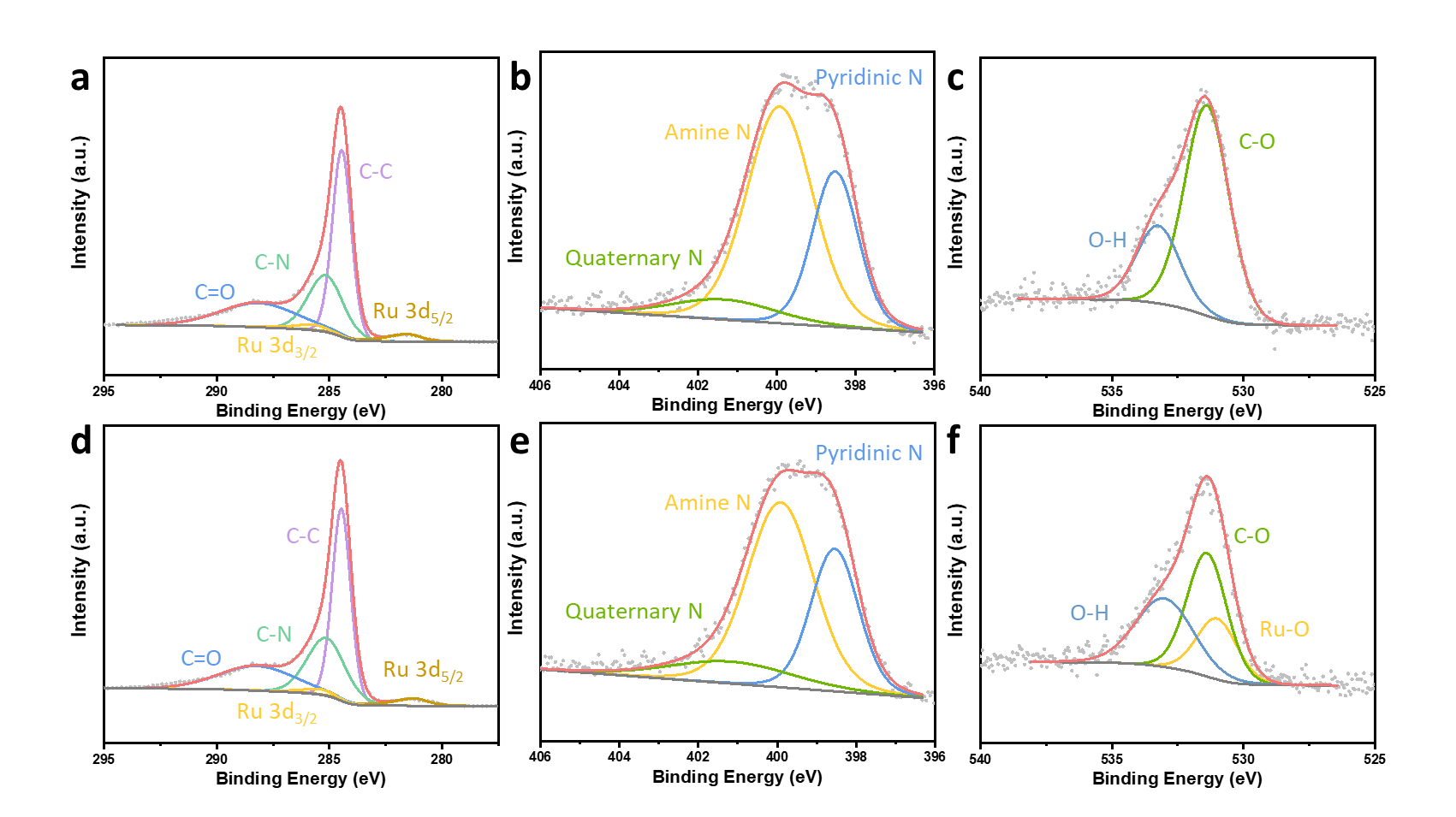


**Figure S12.** XPS spectrum of RuNC_C and RuNC_O.

a) C 1s spectra of RuNC_C b) N 1s spectra of RuNC_C c) O 1s spectra of RuNC_C d) C 1s spectra of RuNC_O e) N 1s spectra of RuNC_O f) O 1s spectra of RuNC_O.


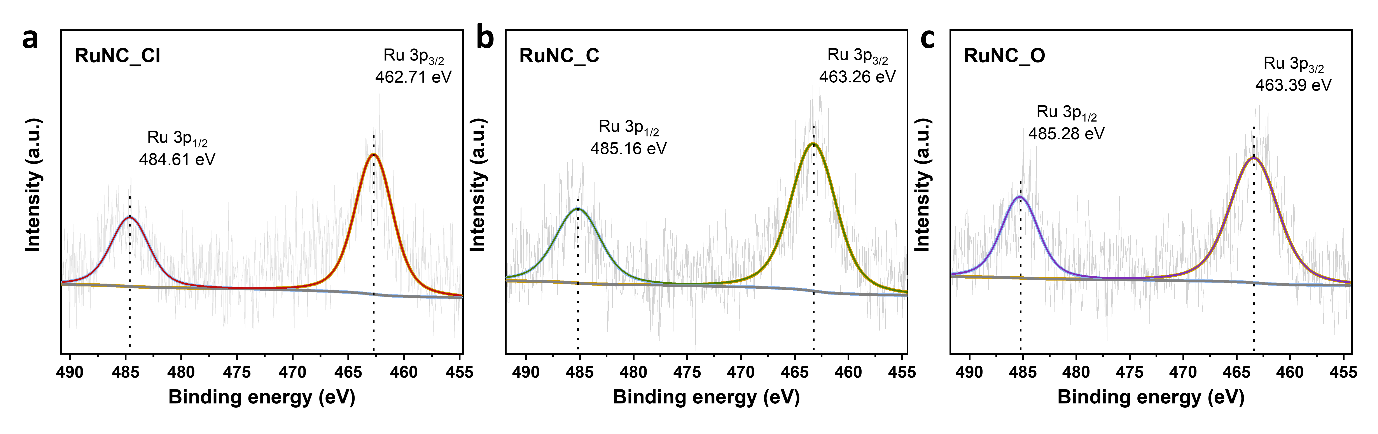


**Figure S13.** Ru 3p XPS spectrum of a) RuNC_Cl b) RuNC_C c) RuNC_O.


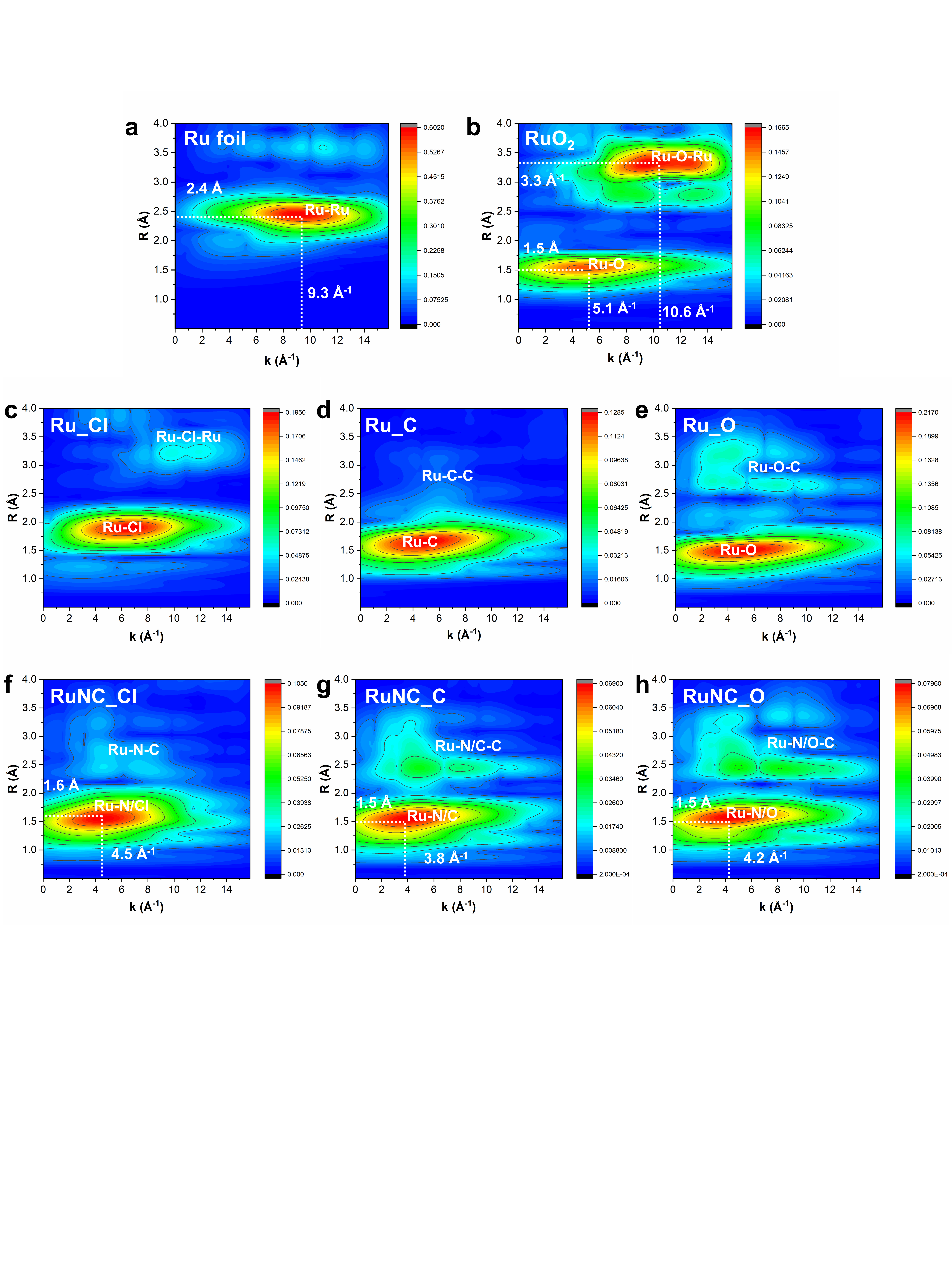


**Figure S14.** Wavelet transform analysis of the Ru EXAFS spectra.

a) Ru foil b) RuO_2_ c) ruthenium(III) chloride (Ru_Cl) d) bis(2-methylallyl)(1,5-cyclooctadiene)ruthenium(II) (Ru_C) e) ruthenium(III) acetylacetonate (Ru_O) f) RuNC_Cl g) RuNC_C h) RuNC_O.


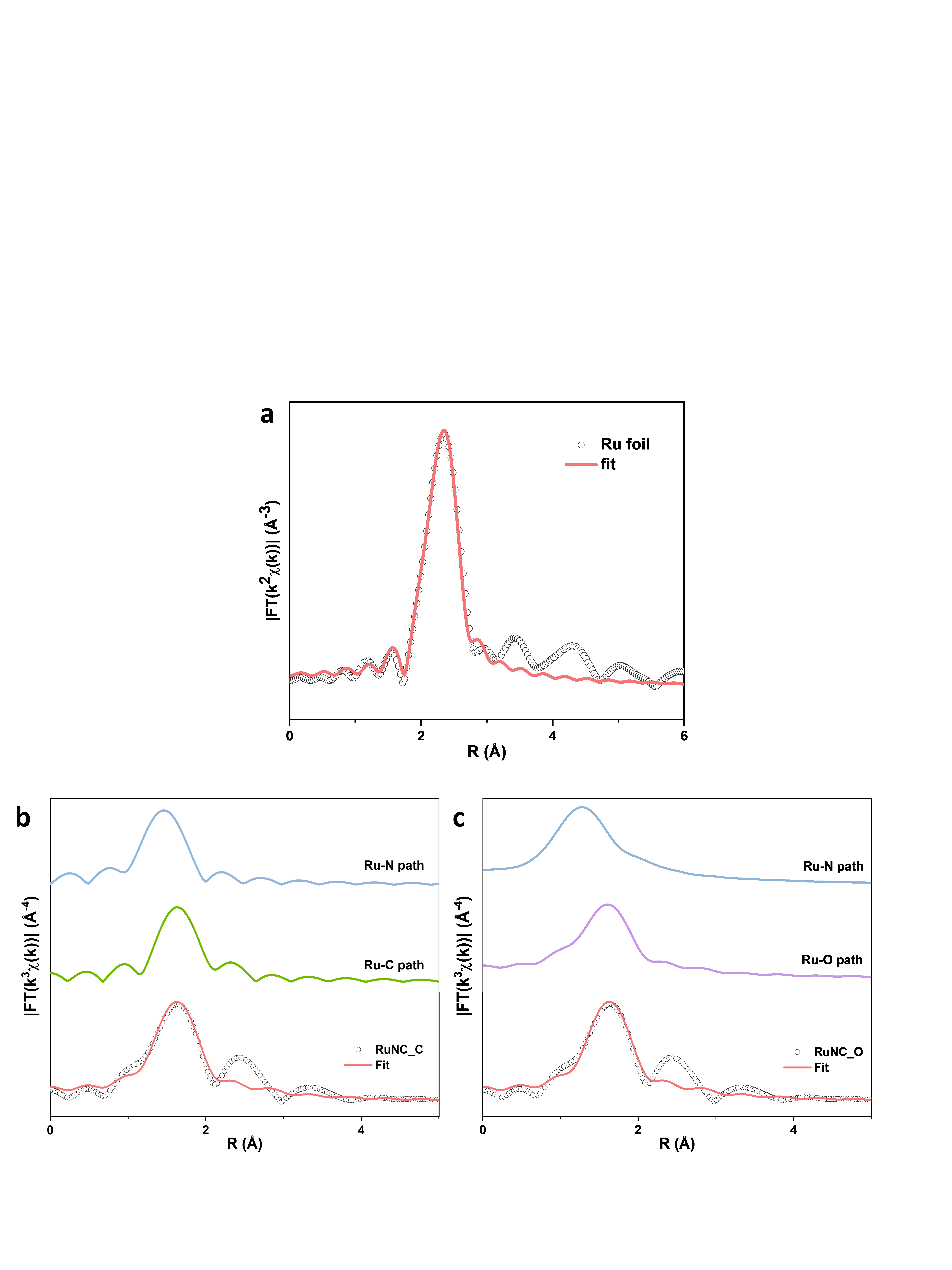


**Figure S15.** Fitting of the EXAFS spectra of Ru foil and RuSAzymes in R space.

a) Ru foil b) RuNC_C c) RuNC_O.


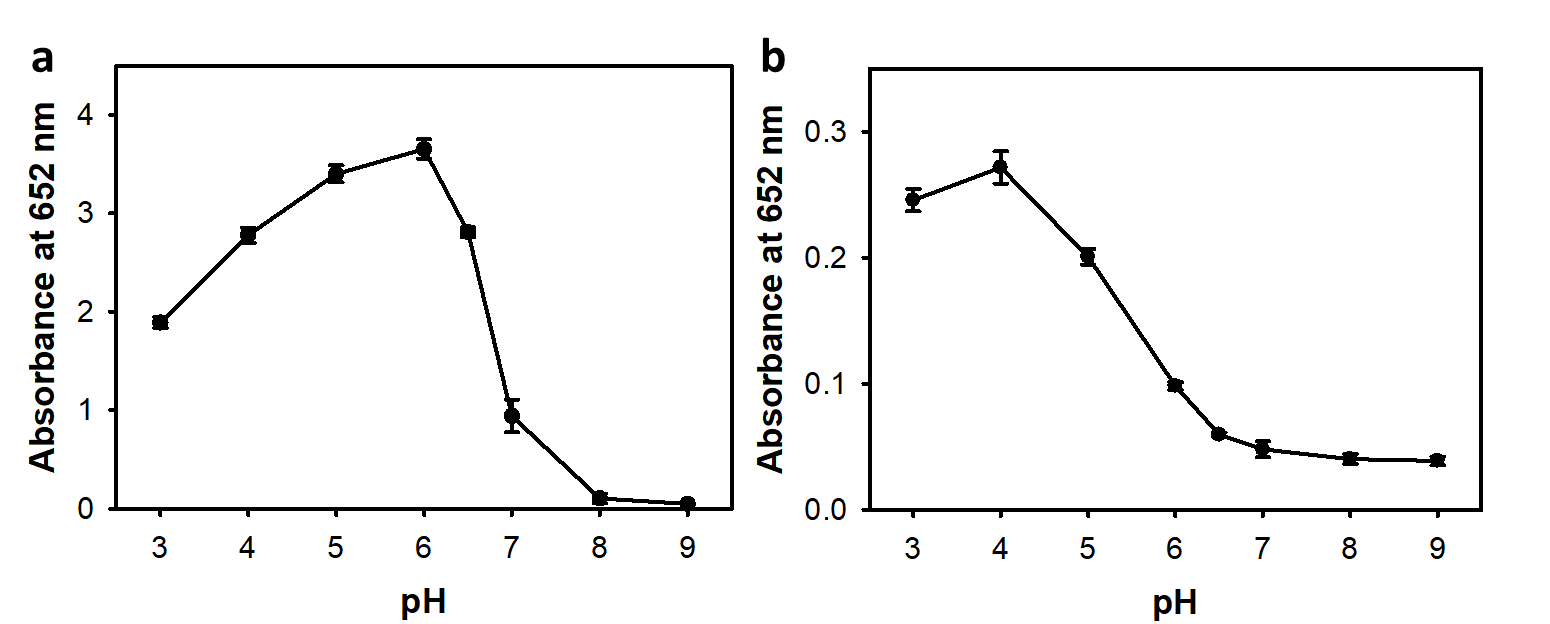


**Figure S16.** Absorbance at 652 nm under various pH.

a) POD-like reaction. b) OXD-like reaction.


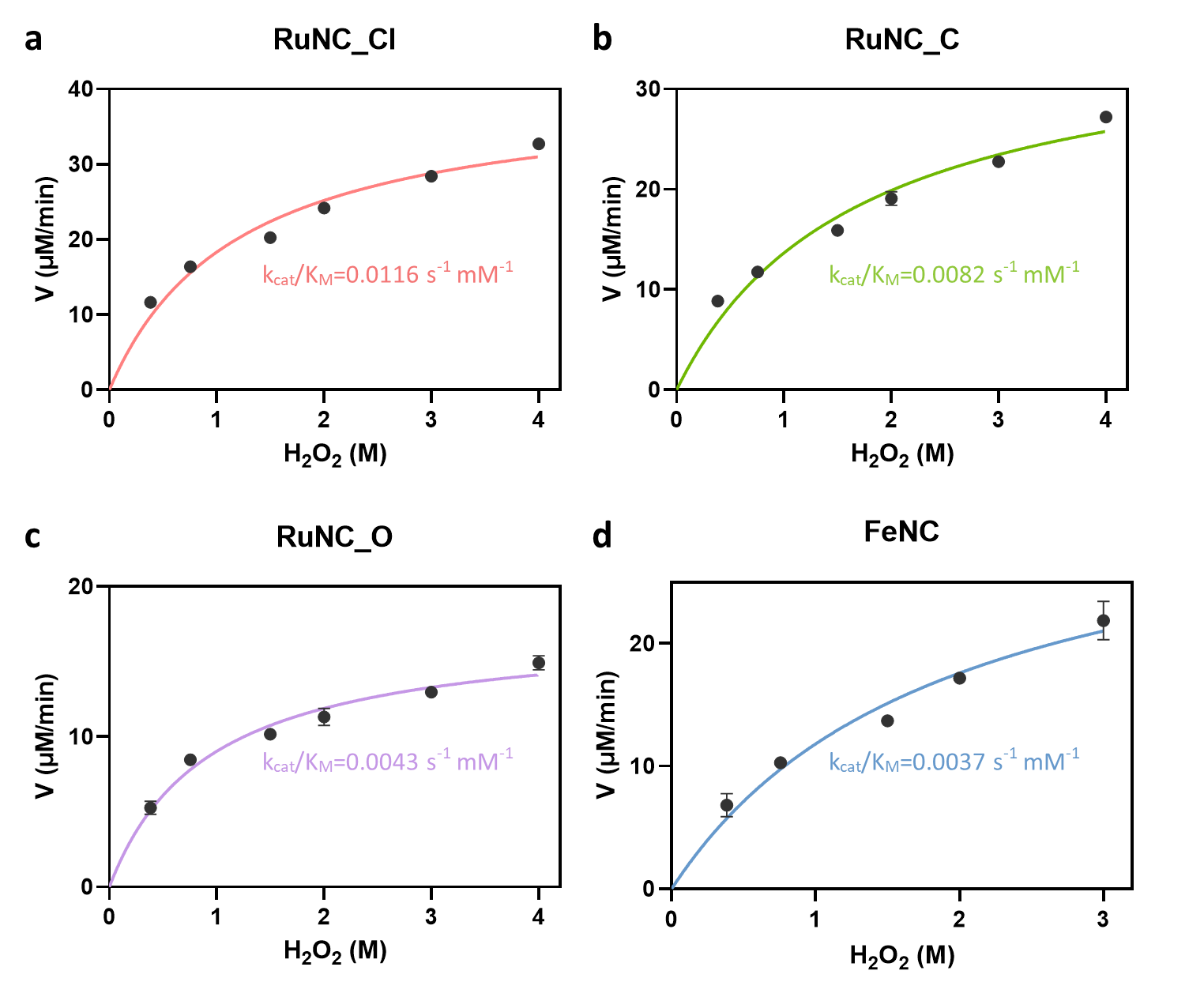


**Figure S17.** Steady-state kinetic assay plots using H_2_O_2_ as substrate.

a) RuNC_Cl b) RuNC_C c) RuNC_O d) FeNC.


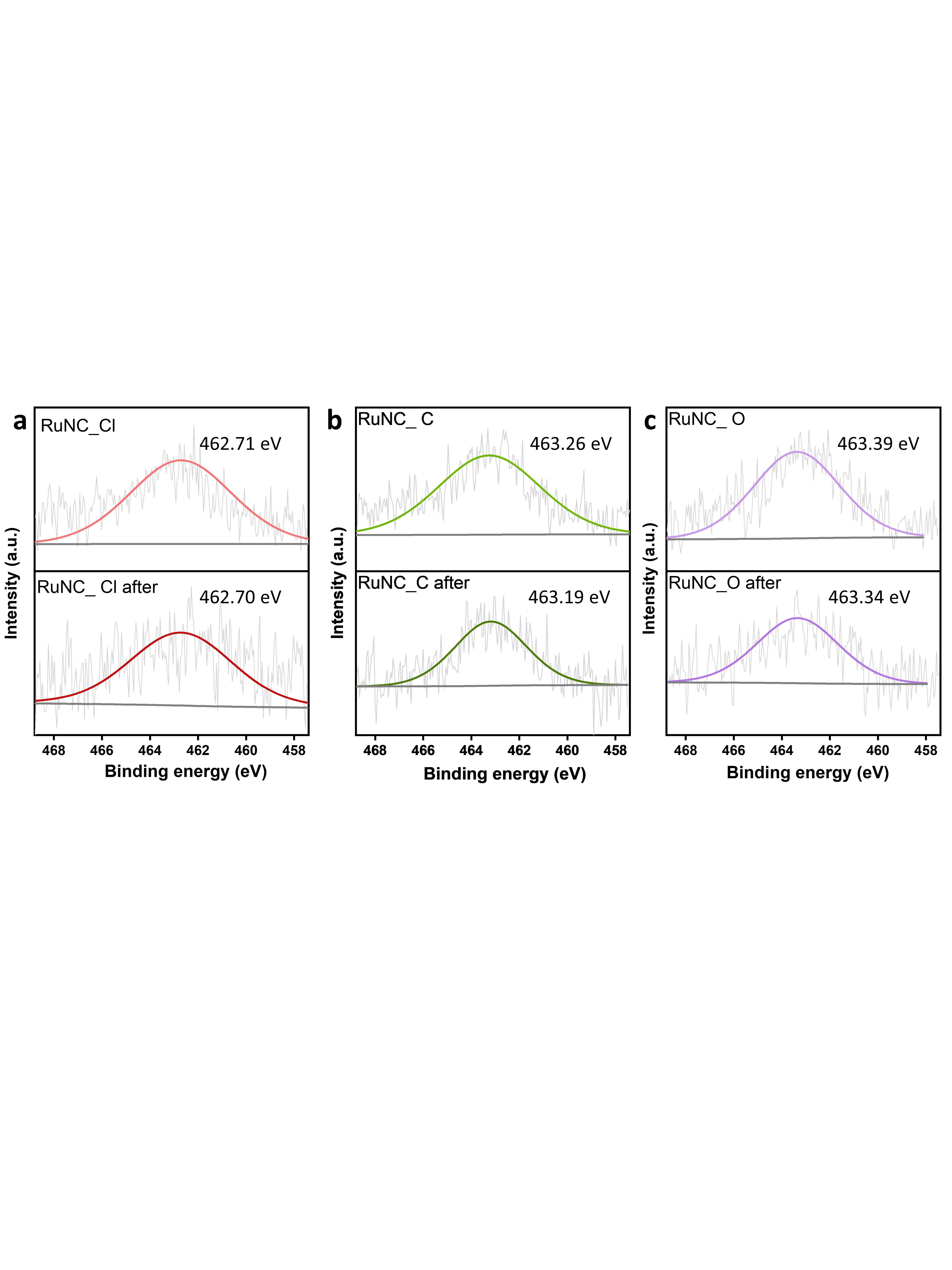


**Figure S18.** Comparison of Ru 3p₃/₂ XPS spectra before and after reaction for a) RuNC_Cl b) RuNC_C c) RuNC_O.


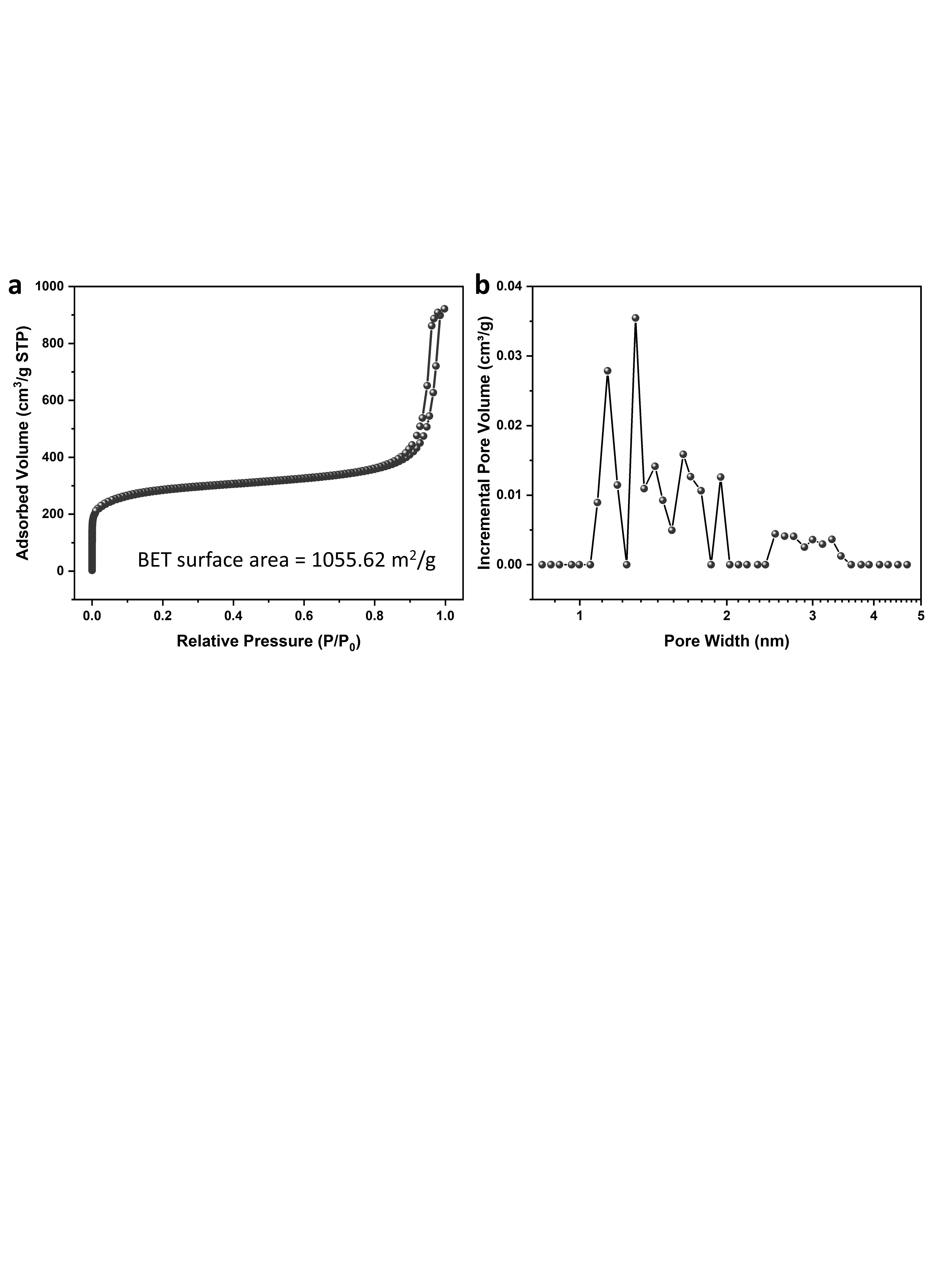


**Figure S19.** BET analysis of NC.

a) Nitrogen adsorption-desorption isotherms of NC b) Pore size distributions of NC.


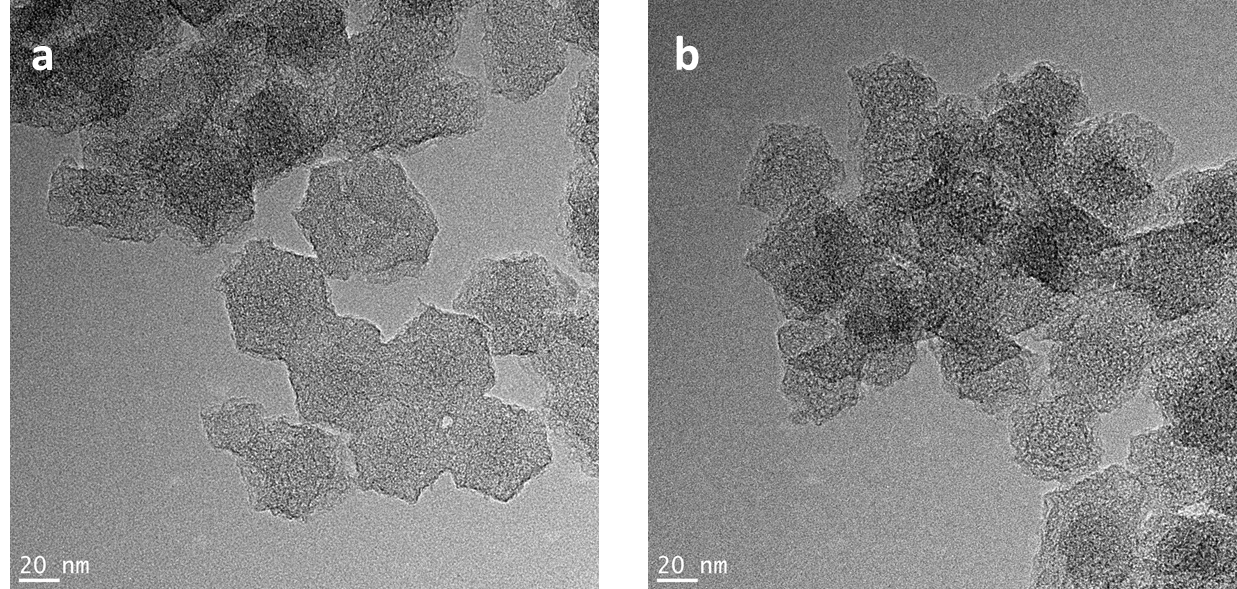


**Figure S20.** TEM images of NC and FeNC.

a) NC (scale bar =20 nm) b) FeNC (scale bar =20 nm).


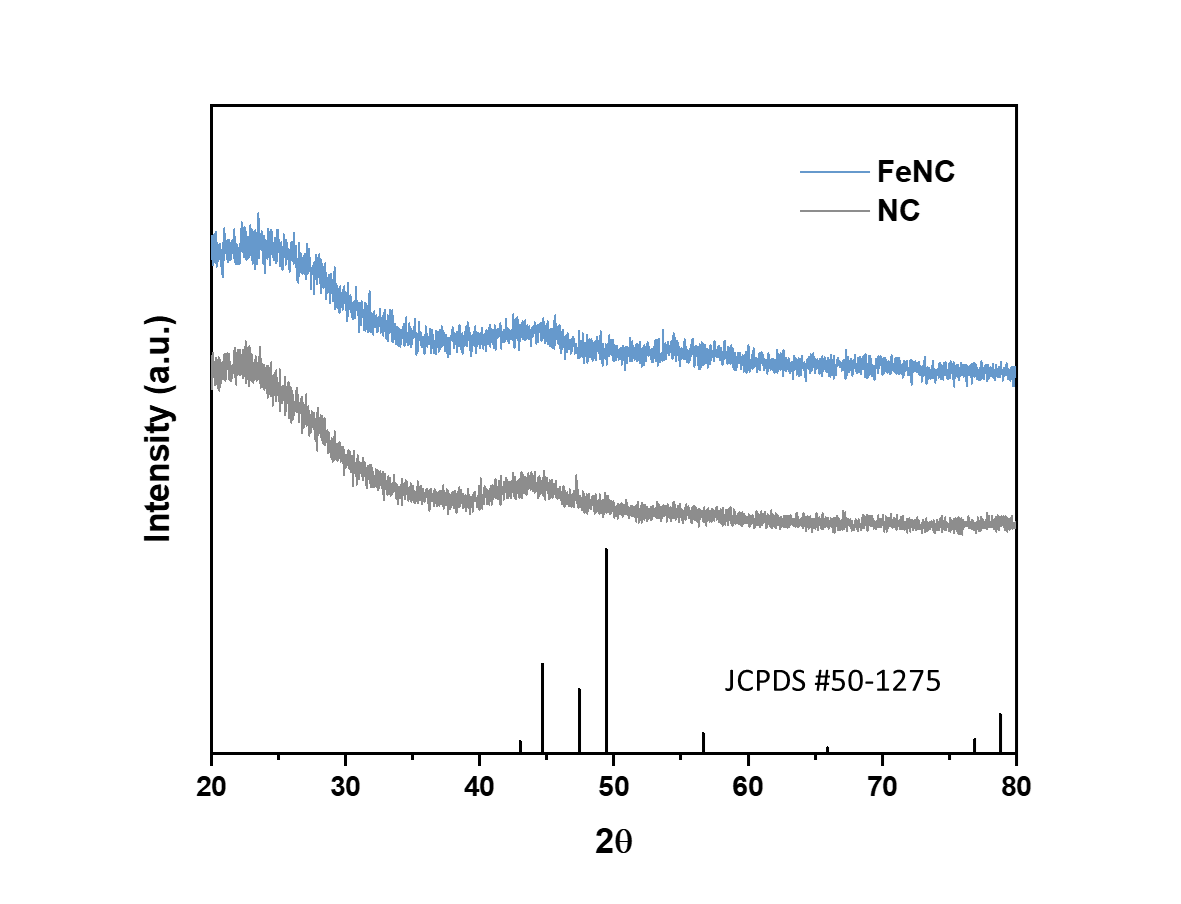


**Figure S21.** X-ray diffraction patterns of FeNC (JCPDS #50-1275 means hexagonal Fe metal).


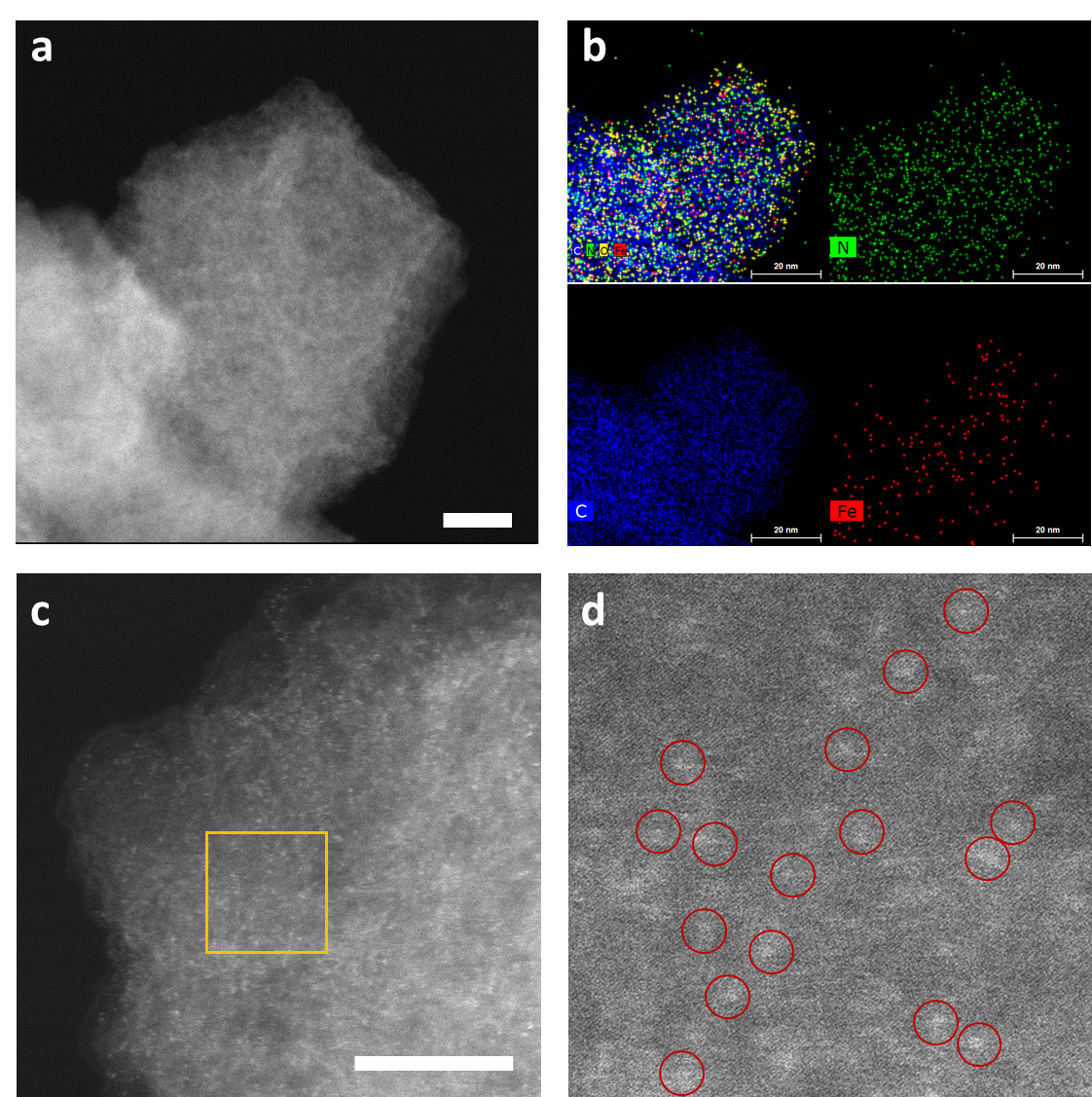


**Figure S22.** HAADF STEM images and Energy-dispersive X-ray elemental(EDX) mapping of FeNC.

a) HAADF STEM image (scale bar =10 nm) b) The corresponding EDX mapping of a) (scale bar =20 nm) c) HAADF STEM image (scale bar =5 nm) d) Enlarged image of highlighted area in c).


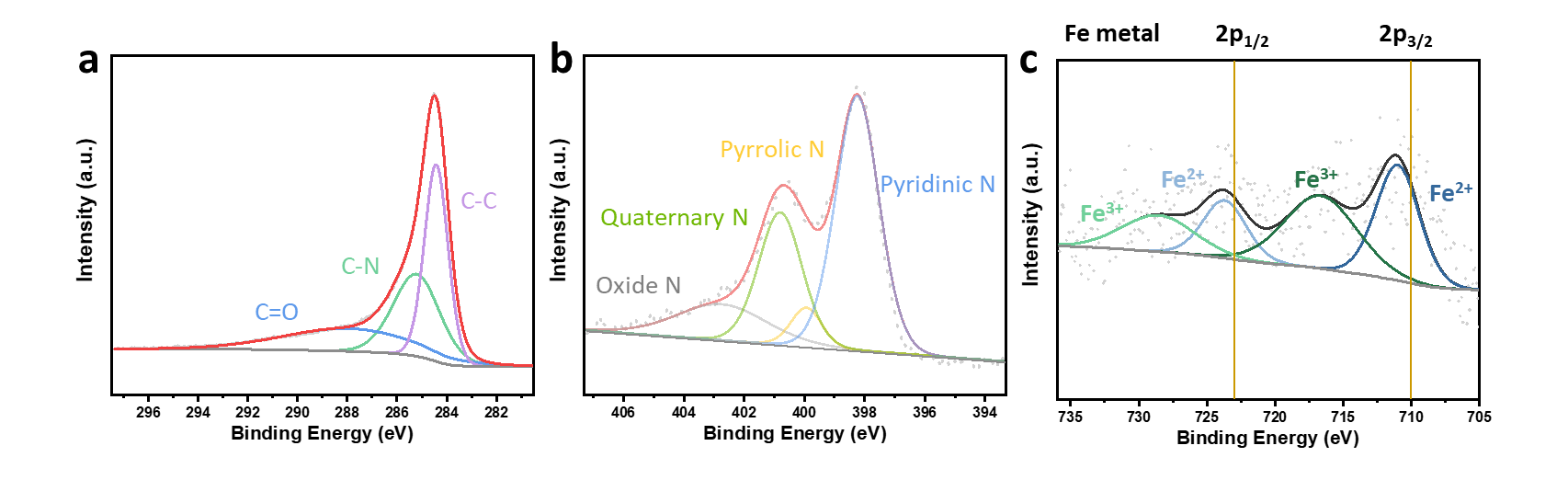


**Figure S23.** XPS spectrum of FeNC.

a) C 1s spectra b) N 1s spectra c) Fe 2p spectra.


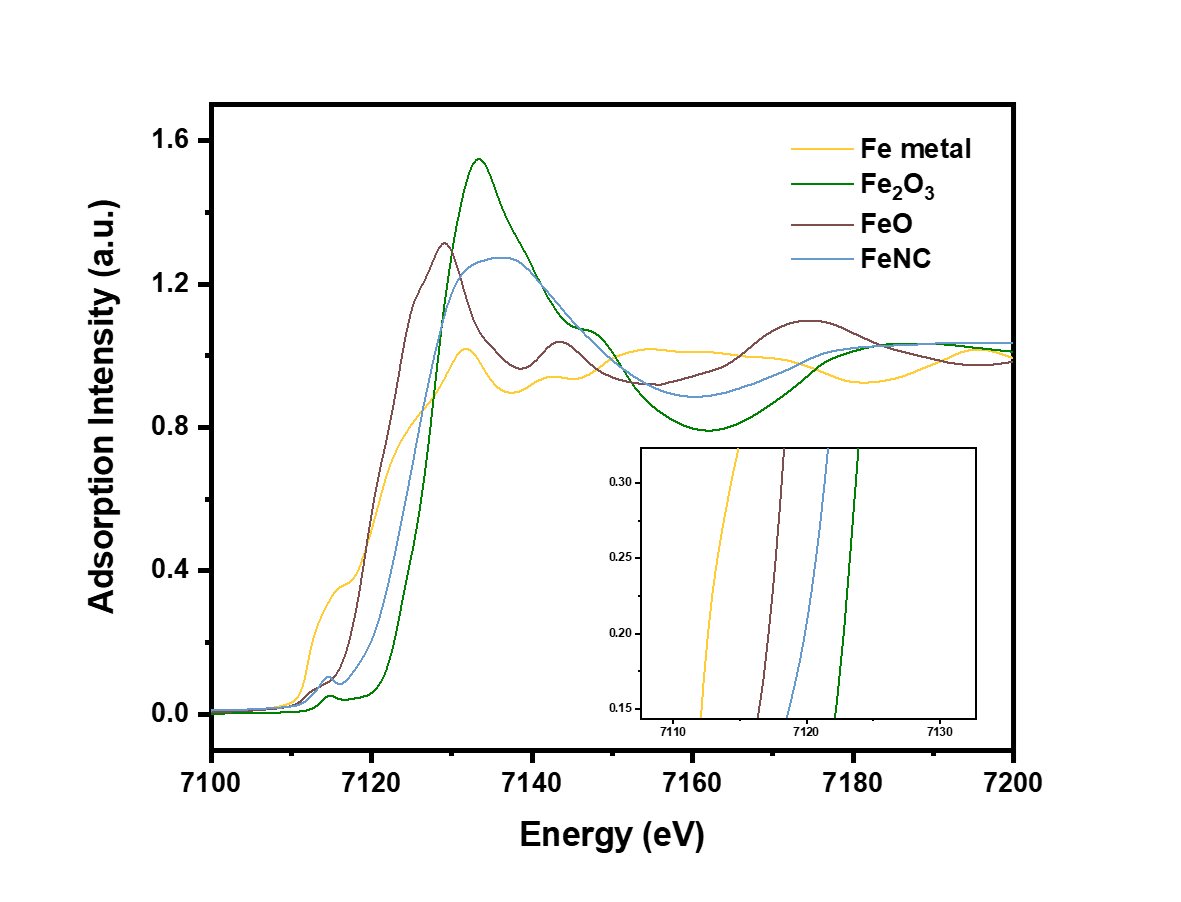


**Figure S24.** Normalized X-ray absorption near-edge structure (XANES) spectra of Fe K-edge**.**

**
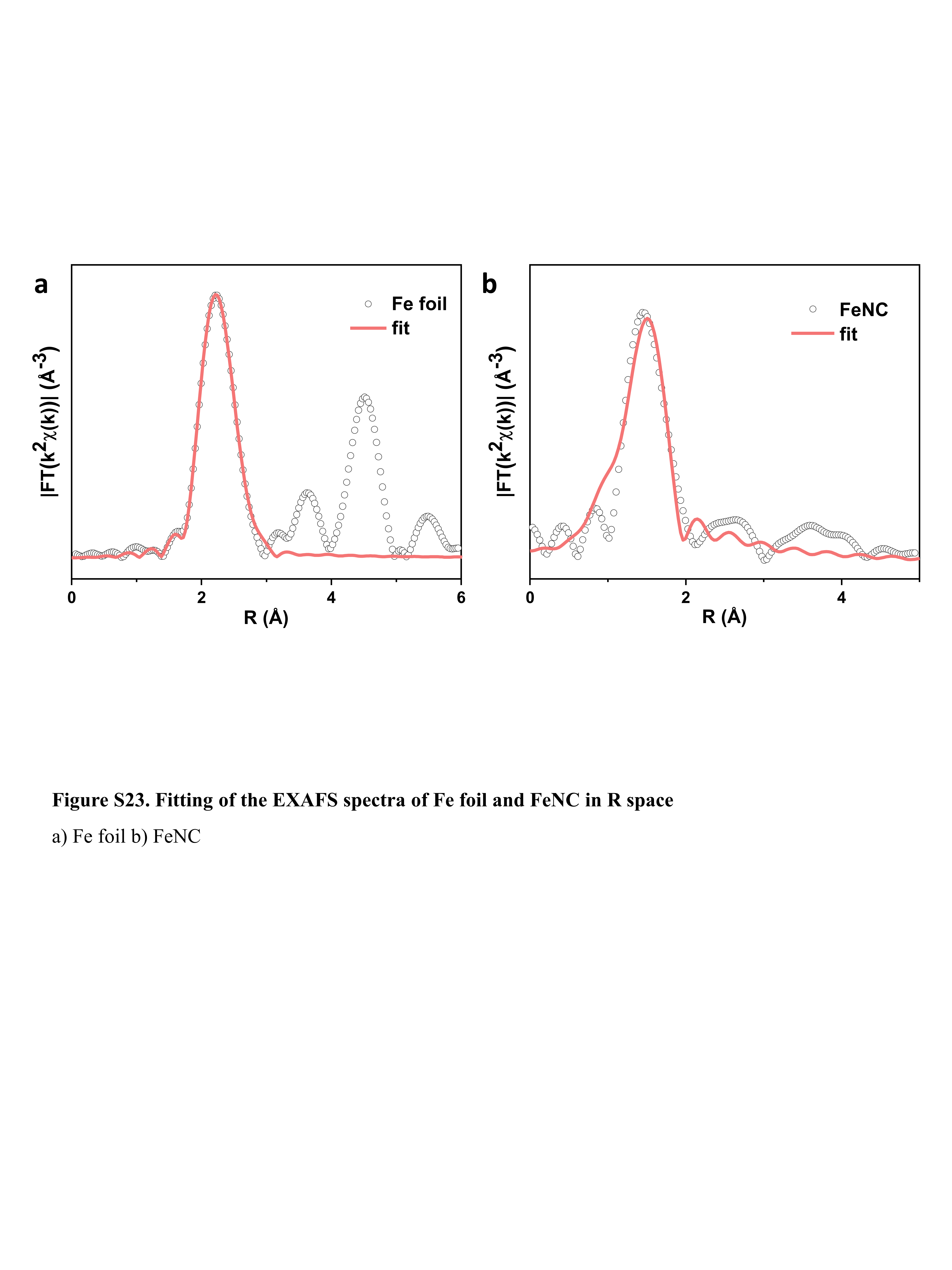
**

**Figure S25.** Fitting of the EXAFS spectra of Fe foil and FeNC in R space.

a) Fe foil b) FeNC.

**Supplementary Note 3. Structural and Electronic Characterization of FeNC.**

Nitrogen-doped carbon (NC) was synthesized by pyrolyzing zeolitic imidazolate framework-8 (ZIF-8) nanocrystals at 1100 °C under an argon atmosphere. FeNC was then prepared by adsorbing Fe^3+^ ions onto NC, followed by annealing at 700 °C under argon flow. During the heat treatment, Zn species from the ZIF-8 framework were volatilized, creating abundant nitrogen coordination sites (N_4_) that serve as anchoring sites for Fe ions. BET analysis revealed a large specific surface area and a well-defined microporous structure of NC (**Figure S19**). TEM images further confirmed that both NC and FeNC maintained a polyhedral morphology with particle sizes ranging from 50 to 80 nm (**Figure S20**).

The atomic dispersion of Fe species was confirmed by several complementary techniques. XRD analysis showed no diffraction peaks corresponding to Fe nanoparticles (**Figure S21**), and HAADF-STEM imaging indicated that Fe was atomically dispersed without cluster formation (**Figure S22**). In the XPS spectra, the Fe 2p peak was positively shifted relative to metallic Fe, indicating the presence of Fe^2+^ and Fe^3+^ species (**Figure S23**). XANES analysis further supported this result, as the half-edge position of FeNC was located between those of FeO and Fe_2_O_3_ (**Figure S24**). To determine the local coordination environment of Fe, FT-EXAFS fitting was performed. The results revealed that Fe was coordinated to four nitrogen atoms in first shell, forming Fe–N_4_ sites (**Figure S25** and **Table S3**). These results confirm the successful formation of atomically dispersed Fe centers, representing a prototypical 2D active site, distinct from the 3D active site featuring out-of-plane ligand coordination in Ru SAzymes.


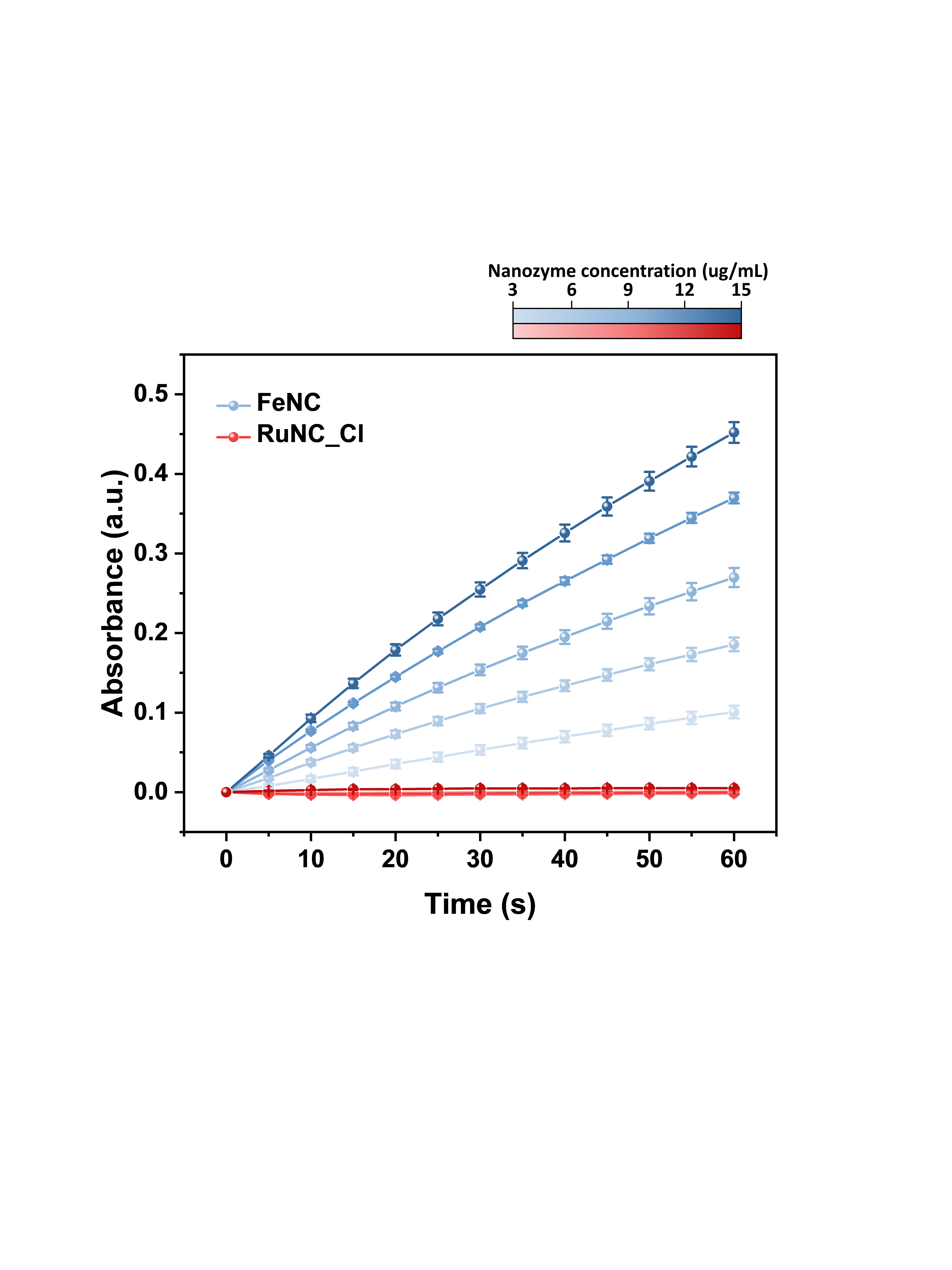


**Figure S26.** Comparison of absorbance at 652 nm under various nanozyme concentration without H_2_O_2._


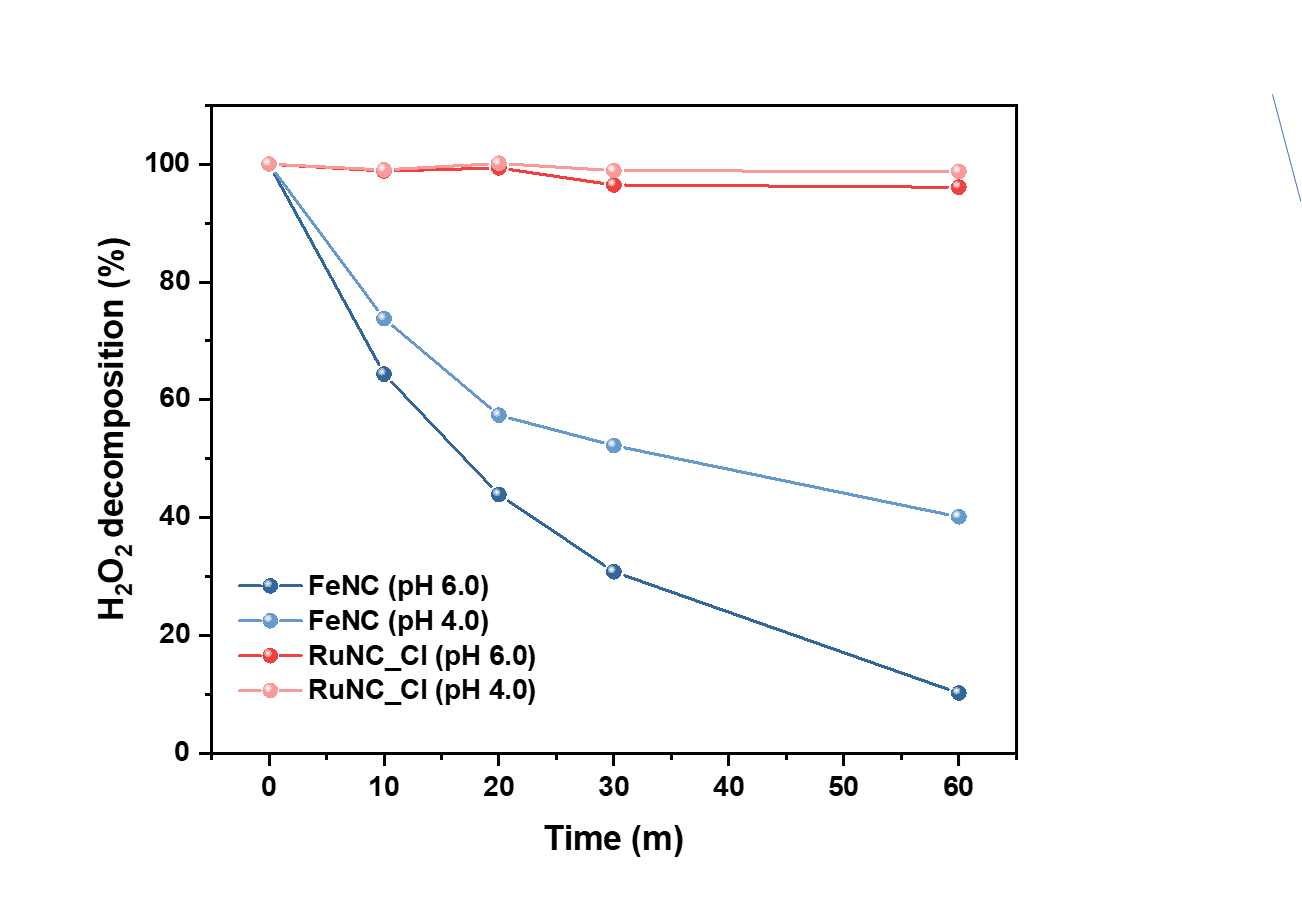


**Figure S27.** H_2_O_2_ decomposition under different pH.


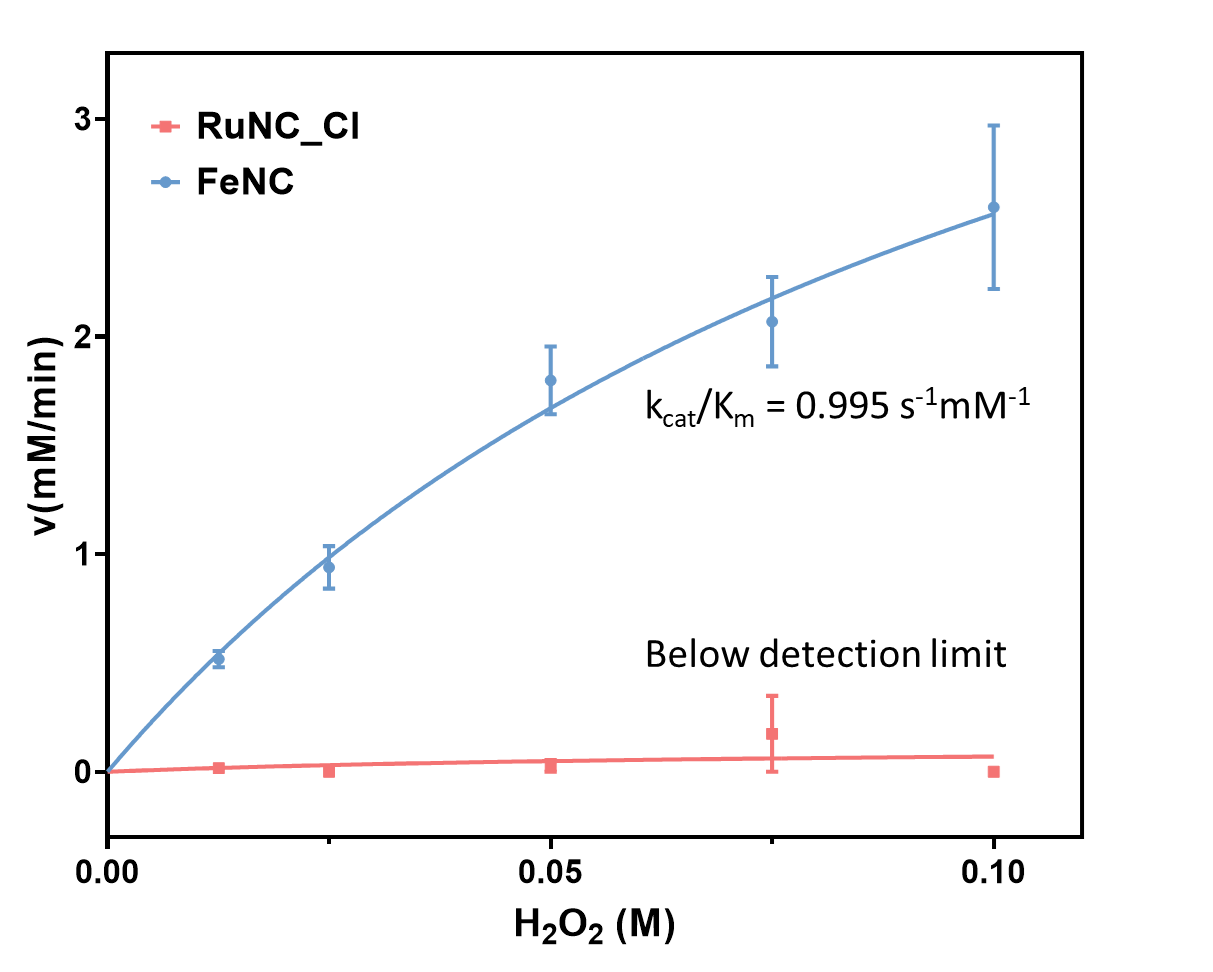


**Figure S28.** Steady-state kinetic assay of CAT-like performance plot using H_2_O_2._


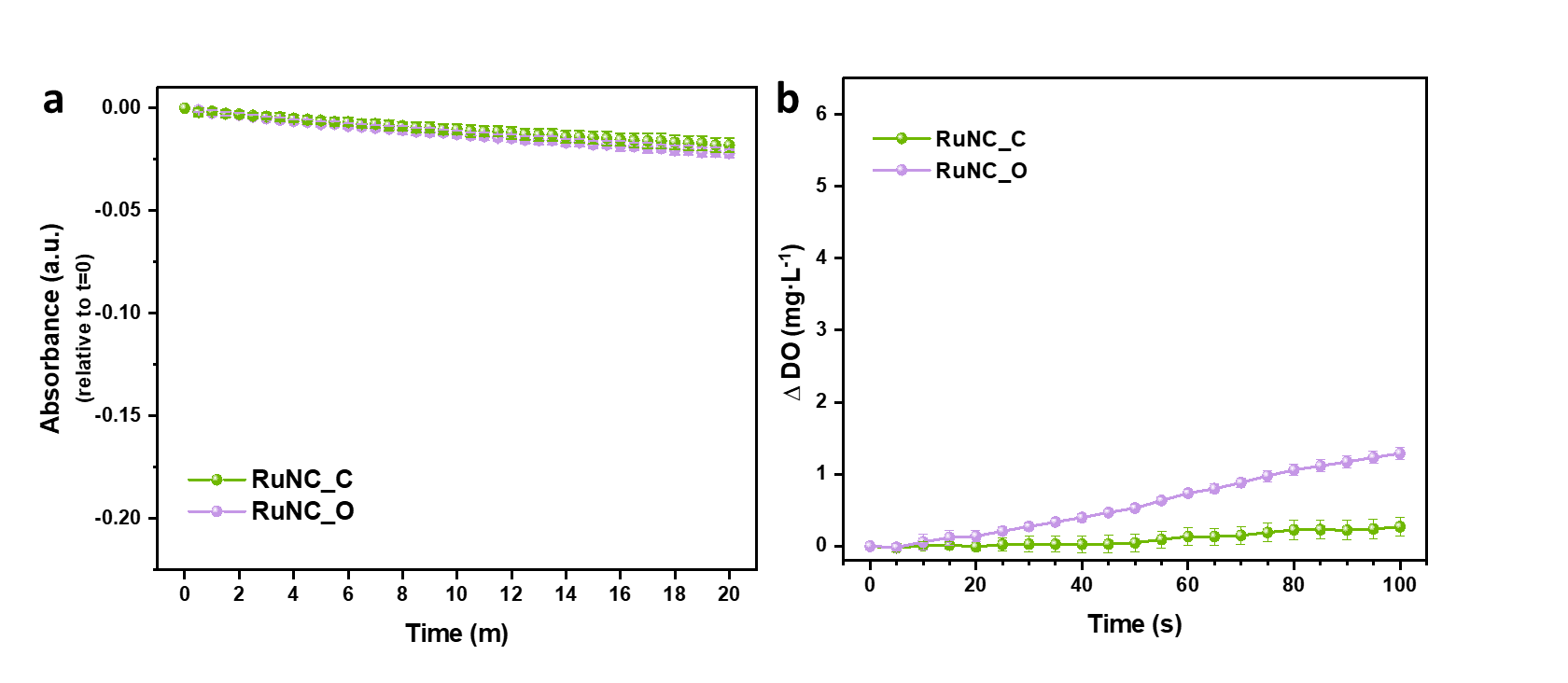


**Figure S29.** Catalase-like performance of RuNC_O and RuNC_C.

a) H_2_O_2_ decomposition rate versus time curves b) O_2_ generation rate from decomposition of H_2_O_2_ versus time curves.


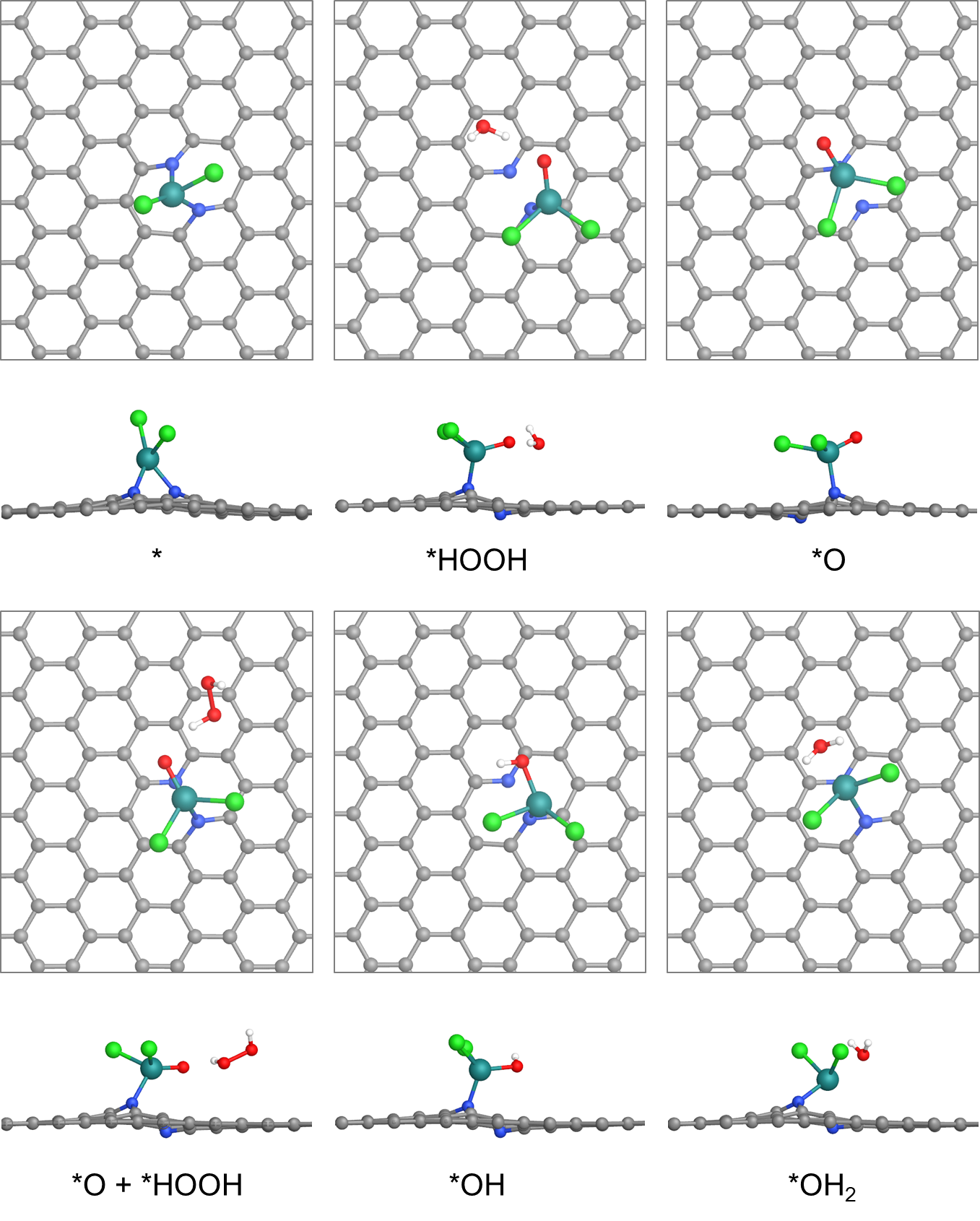


**Figure S30.** Top and side views of POD and CAT intermediates ( *, *HOOH, *O, *O + *HOOH, *OH, *OH_2_ ) of RuNC_Cl.


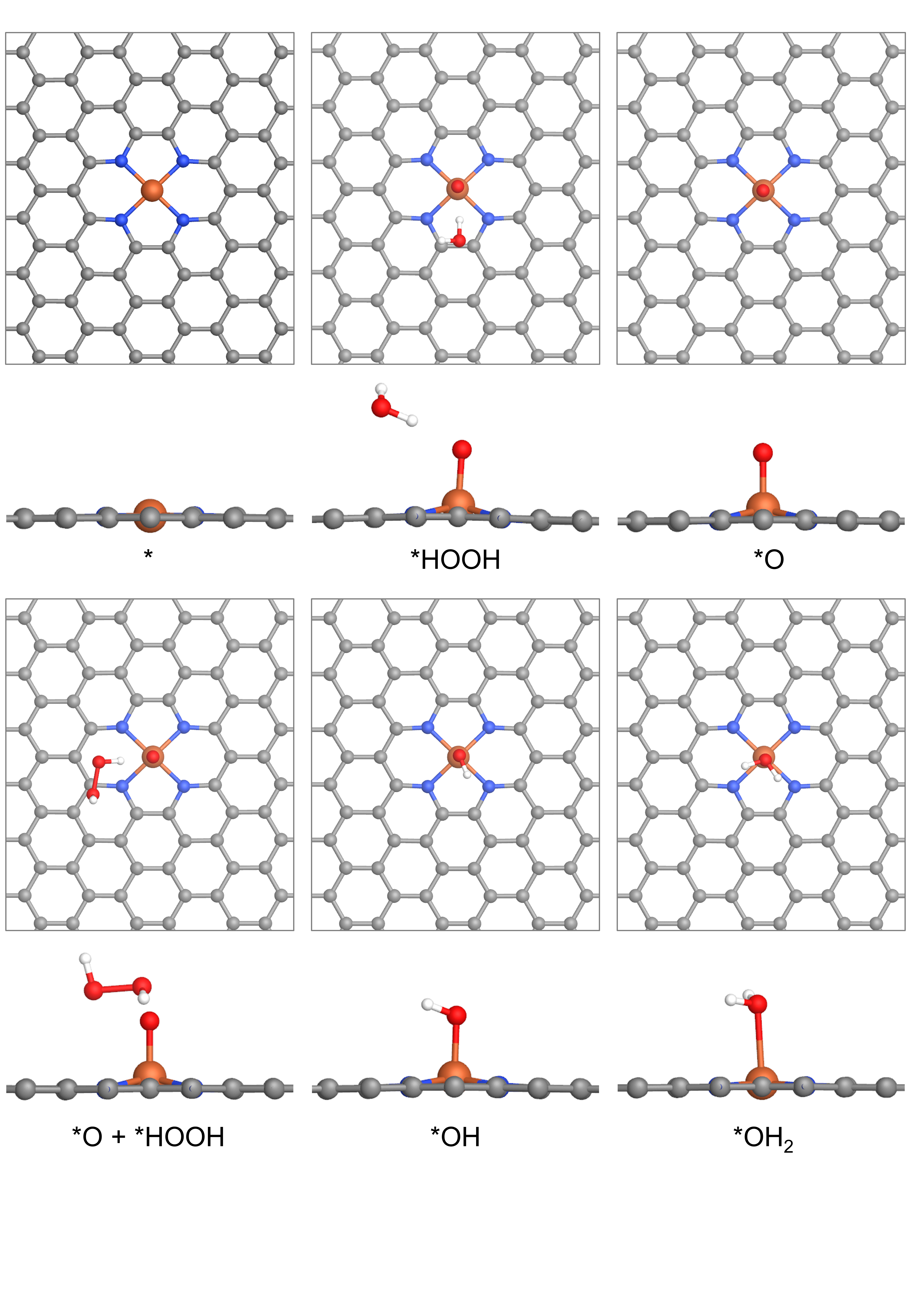


**Figure S31.** Top and side views of POD and CAT intermediates ( *, *HOOH, *O, *O + *HOOH, *OH, *OH_2_ ) of FeNC.


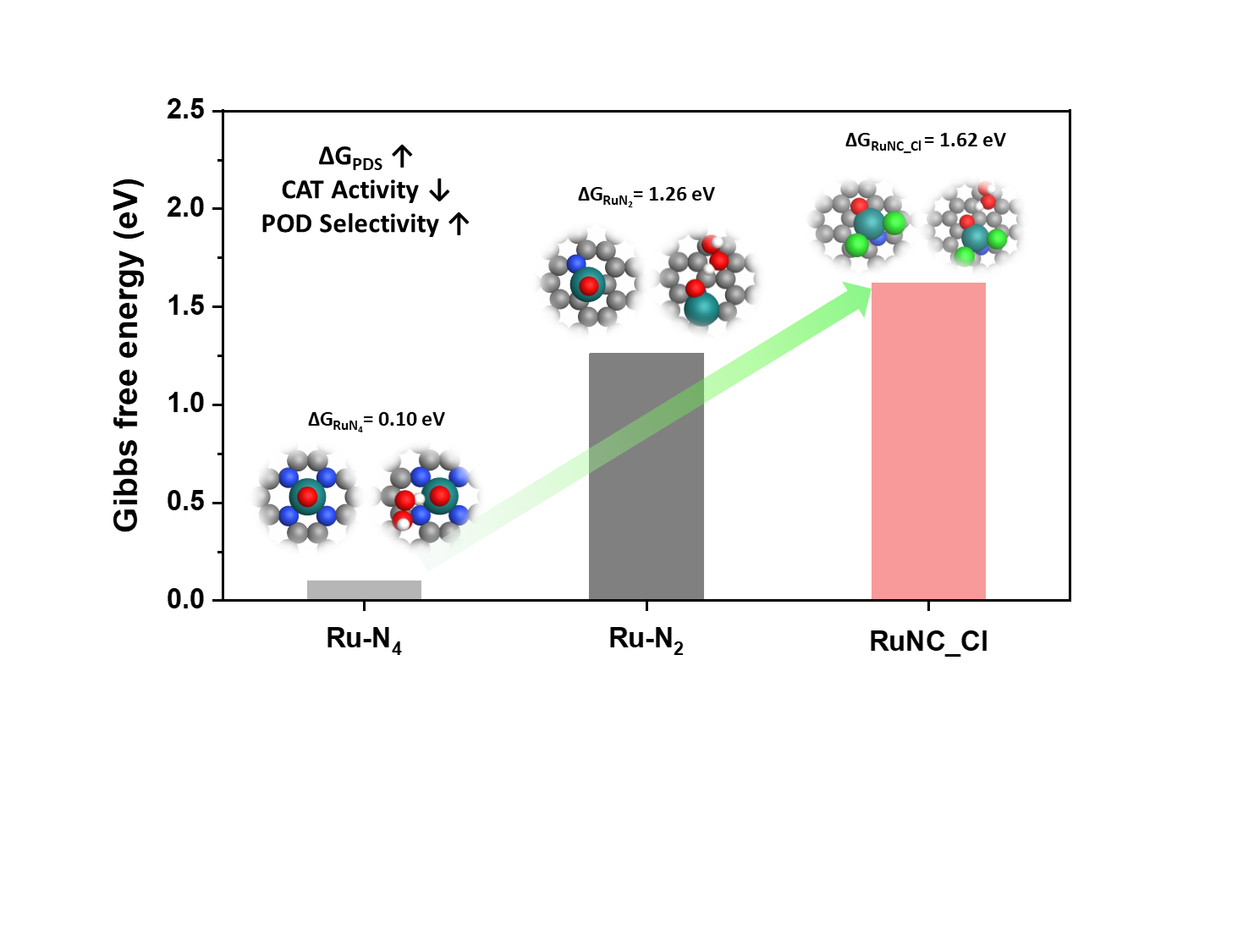


**Figure S32.** Comparison of Gibbs Free Energy Barriers for CAT Reaction PDS Step (∆G_PDS_) (*O 🡪 *O+ *HOOH) in RuN_4_, RuN_2_, and RuNC_Cl.


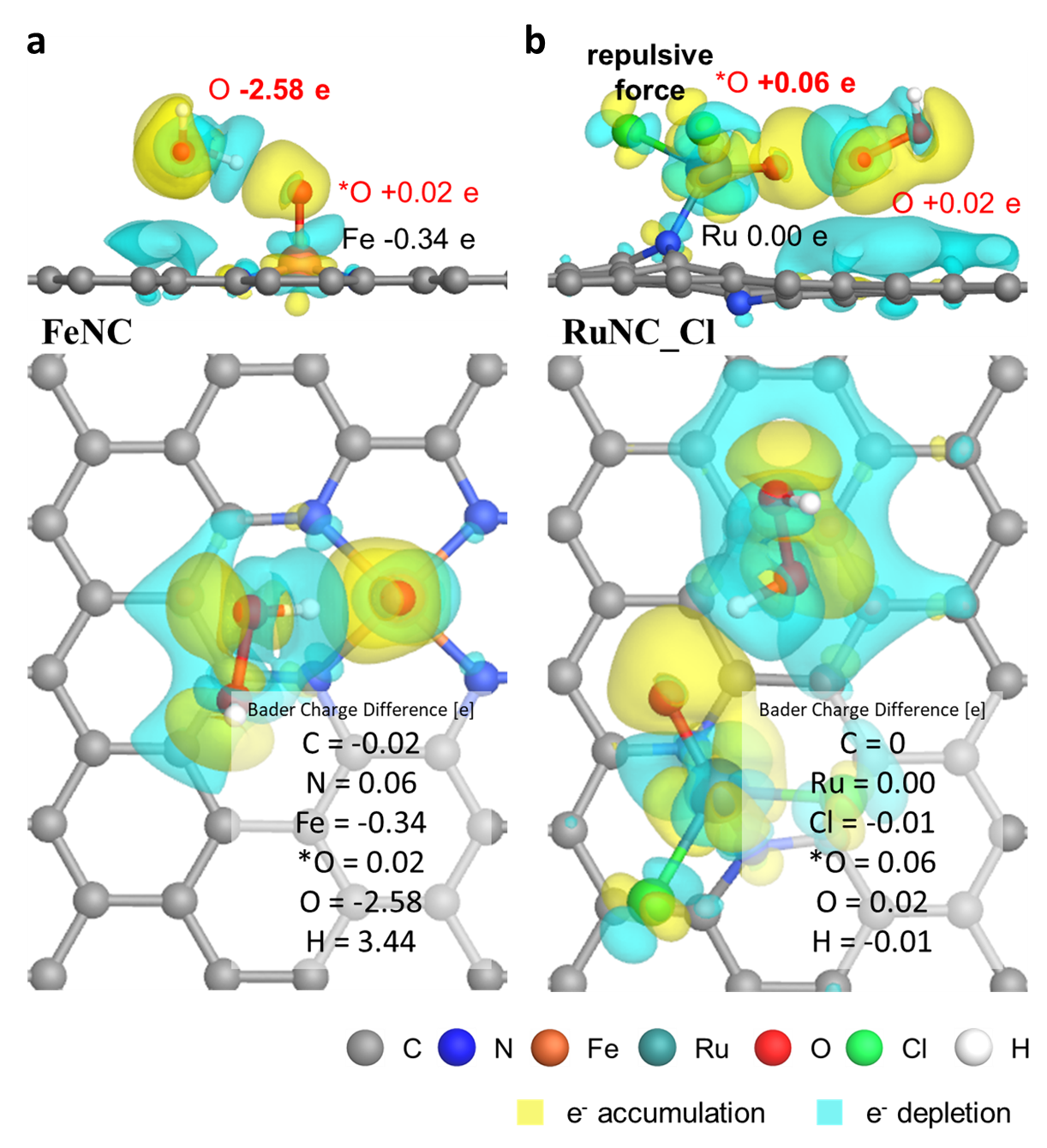


**Figure S33.** Side and top view of Bader charge redistribution of the *O + *HOOH. intermediate of FeNC and RuNC_Cl. Detailed charge density difference values are seen as above.


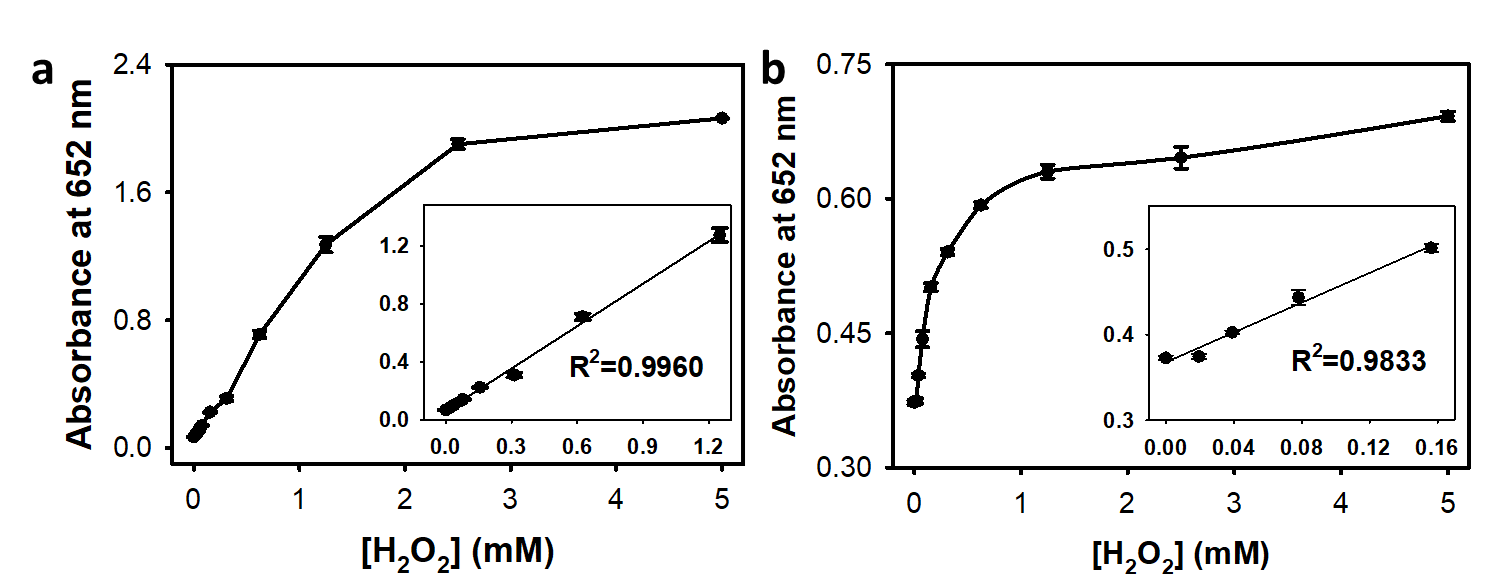


**Figure S34.** Solution-based H_2_O_2_ detection using **a)** RuNC_Cl and **b)** FeNC.


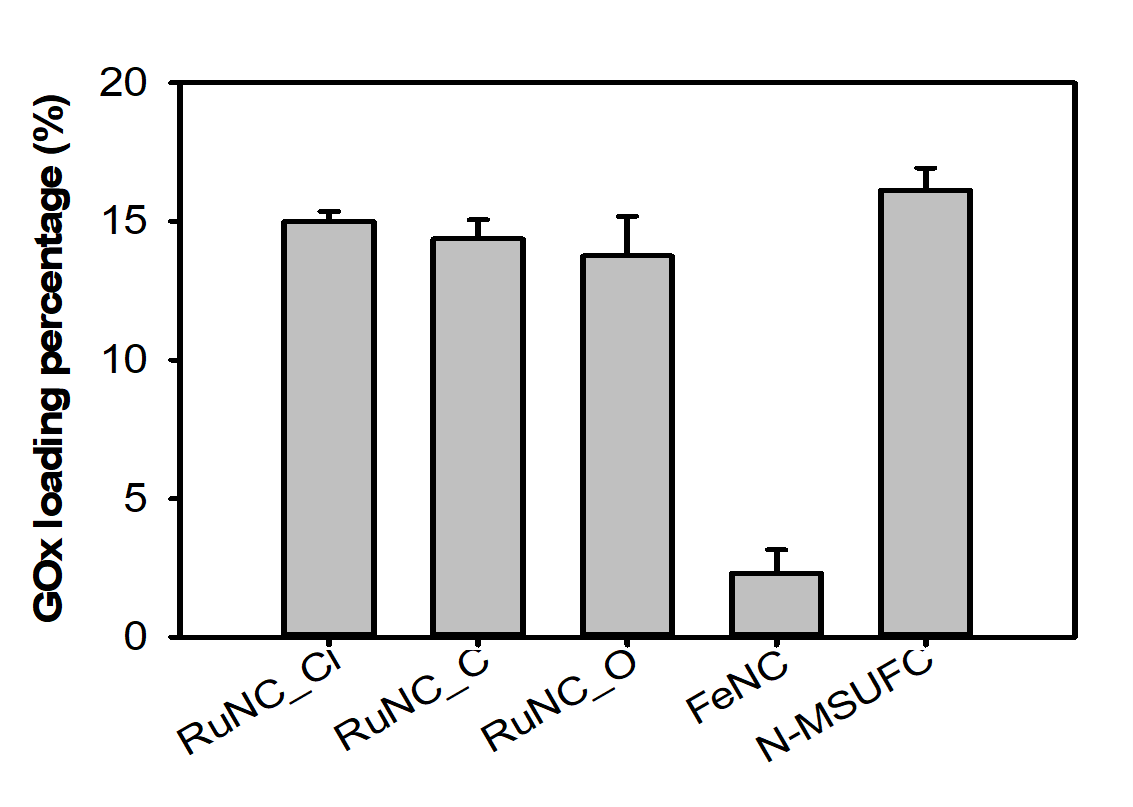


**Figure S35.** Glucose Oxidase (GOx) loading percentage of Ru SAzymes and FeNC.


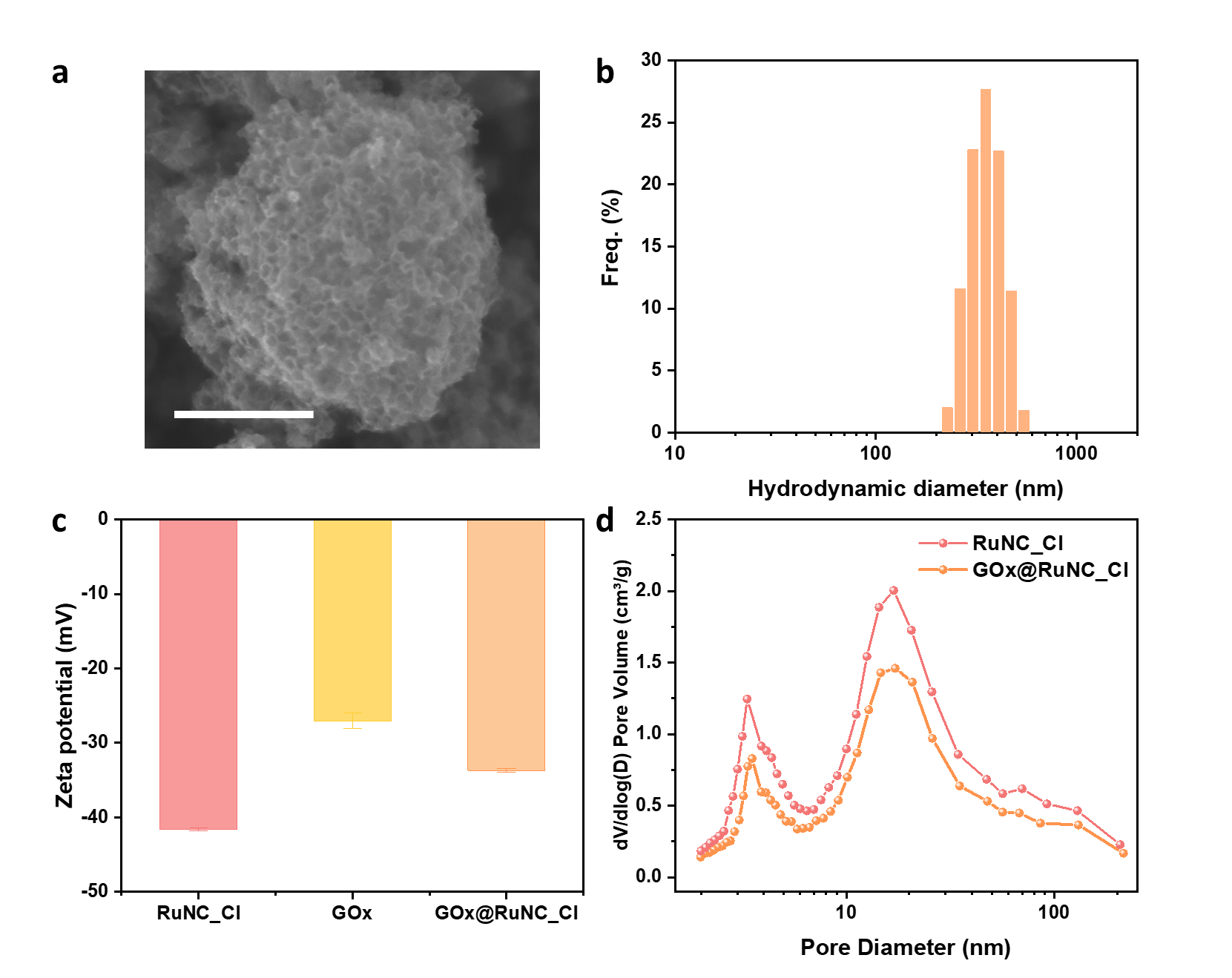


**Figure S36.** Characteriazion of Glucose oxidase loaded RuNC_Cl (GOx@RuNC_Cl).

a) SEM image (scale bar =200 nm) b) DLS analysis c) Zeta potential analysis d) Pore distribution measured by BET analysis.

**Supplementary Note 4.** Characterization of Glucose oxidase loaded RuNC_Cl (GOx@RuNC_Cl) using CLEA method.

CLEA is a widely adopted enzyme immobilization strategy that involves the formation of physical enzyme precipitates followed by their cross-linking with a difunctional agent, typically glutaraldehyde. This immobilization technique is particularly useful when enzyme molecules are crosslinked within the pores of nanoparticles, yielding high loading capacity with enhanced stability and shelf life.To verify the successful immobilization of GOx onto RuNC_Cl, a series of structural and physicochemical characterizations were performed. SEM analysis revealed no visible aggregation or deposition of enzyme molecules on the external surface of RuNC_Cl (**Figure S36a**). DLS measurements showed that the hydrodynamic diameter of GOx@RuNC_Cl (~350 nm) remained comparable to that of RuNC_Cl before loading (**Figure S36b**), further supporting the absence of surface aggregation. The zeta potential of RuNC_Cl was measured at –41.62 mV, whereas free GOx exhibited –27.06 mV. Following GOx loading, the zeta potential of GOx@RuNC_Cl shifted to –33.70 mV (**Figure S36c**), indicating that the presence of GOx modulated the surface charge environment of RuNC_Cl. Given the molecular dimensions of glucose oxidase (6.0 × 5.2 × 7.7 nm^3^), ^[1]^ which are significantly smaller than the mesopore diameter of RuNC_Cl (15.79 nm), the enzyme was expected to enter the pores. This expectation was supported by BET analysis, which showed that although the mesopore diameter remained unchanged after enzyme loading, the overall pore volume decreased (**Figure S36d**). The absence of morphological and size changes, combined with the reduction in pore volume, strongly indicates that GOx molecules were successfully encapsulated within the mesopores of RuNC_Cl.


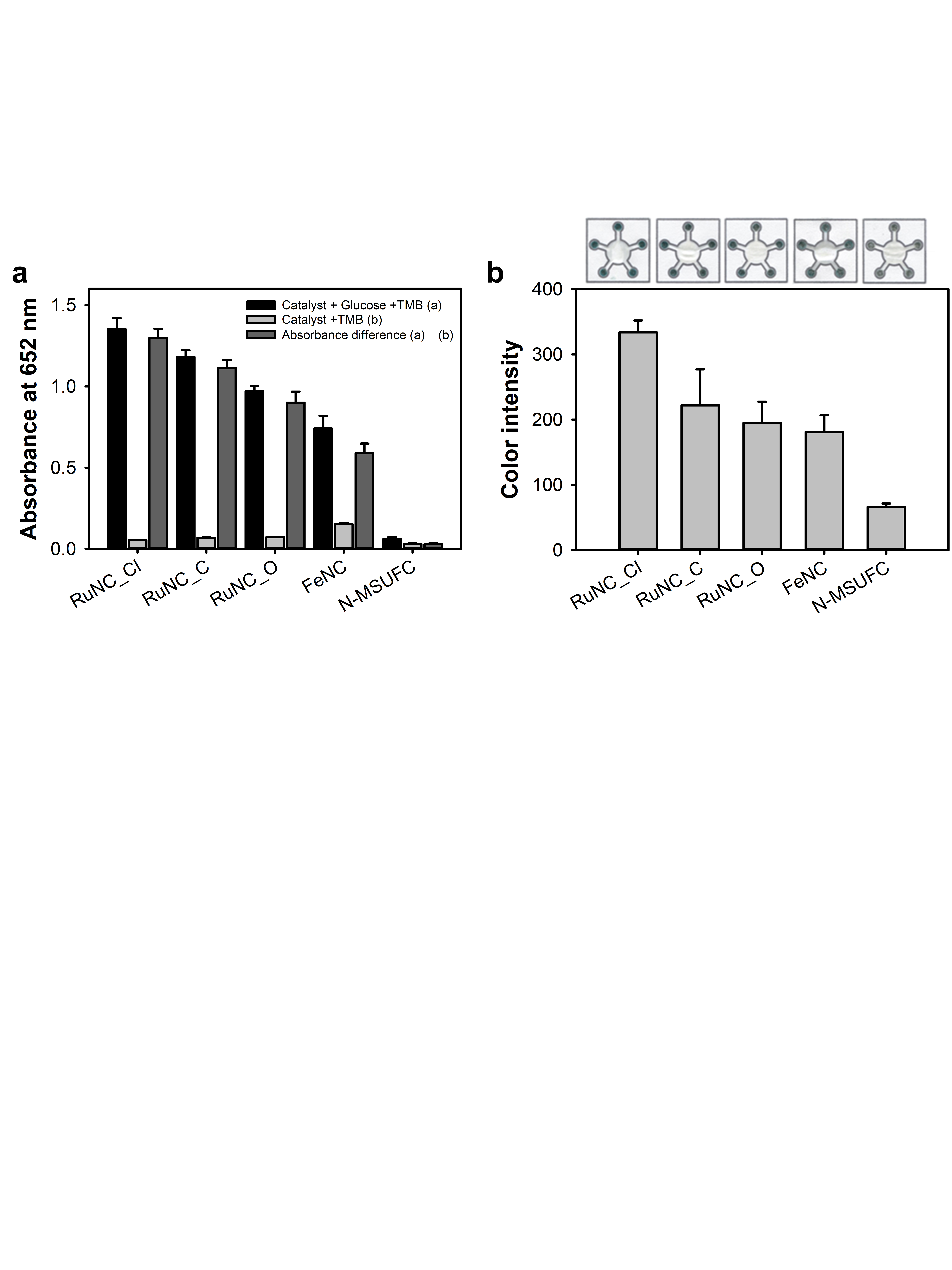


**Figure S37.** Absorbance at 652 nm of Ru SAzymes and FeNC composition with GOx toward 5 mM glucose. a) Solution-based detection b) Paper device-based detection.


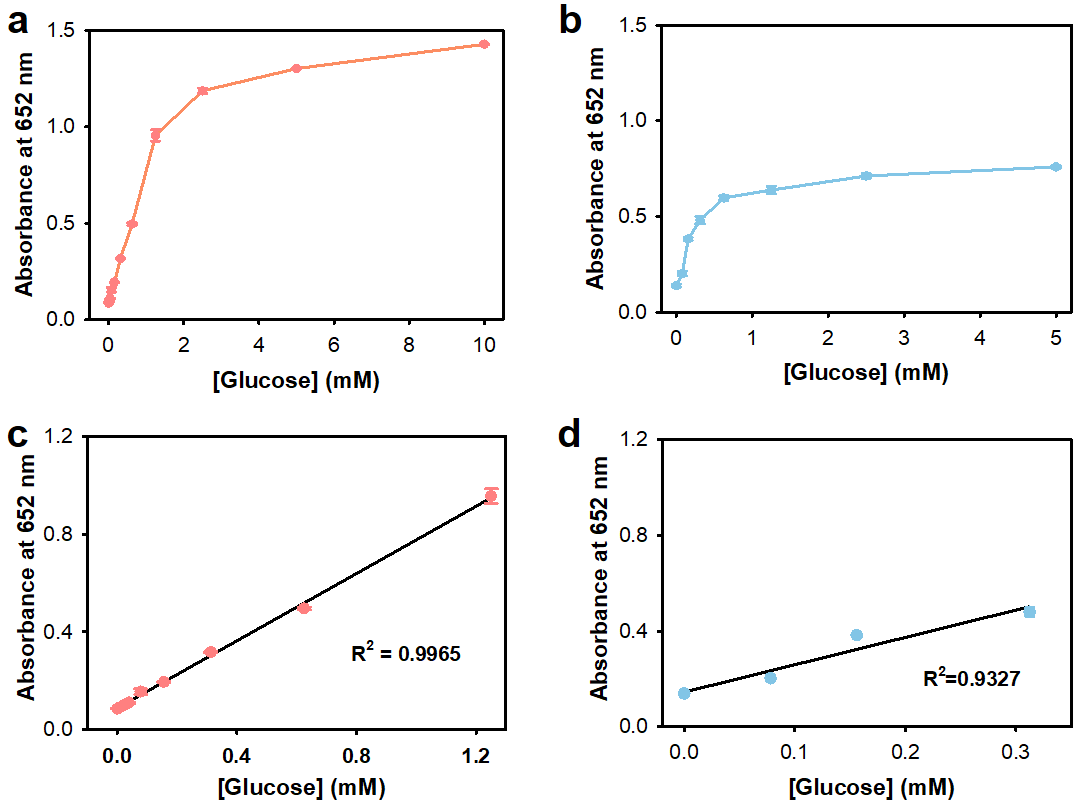


**Figure S38.** Solution-based glucose detection using a, c) GOx@RuNC_Cl and b, d) GOx@FeNC.


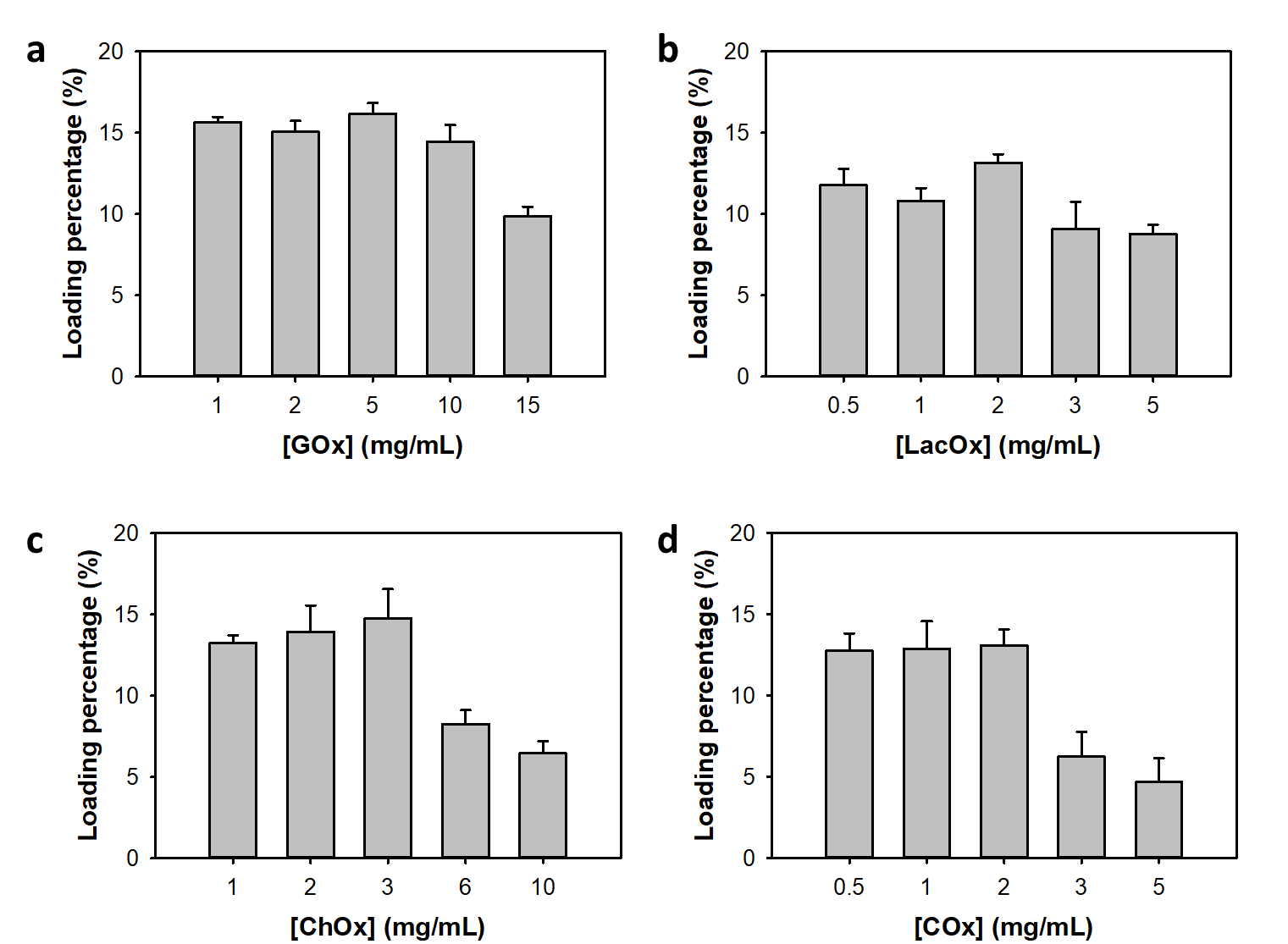


**Figure S39.** Various oxidase enzyme loading percentage of RuNC_Cl.

a) Glucose oxidase b) Lactate oxidase c) Cholesterol oxidase d) Choline oxidase.


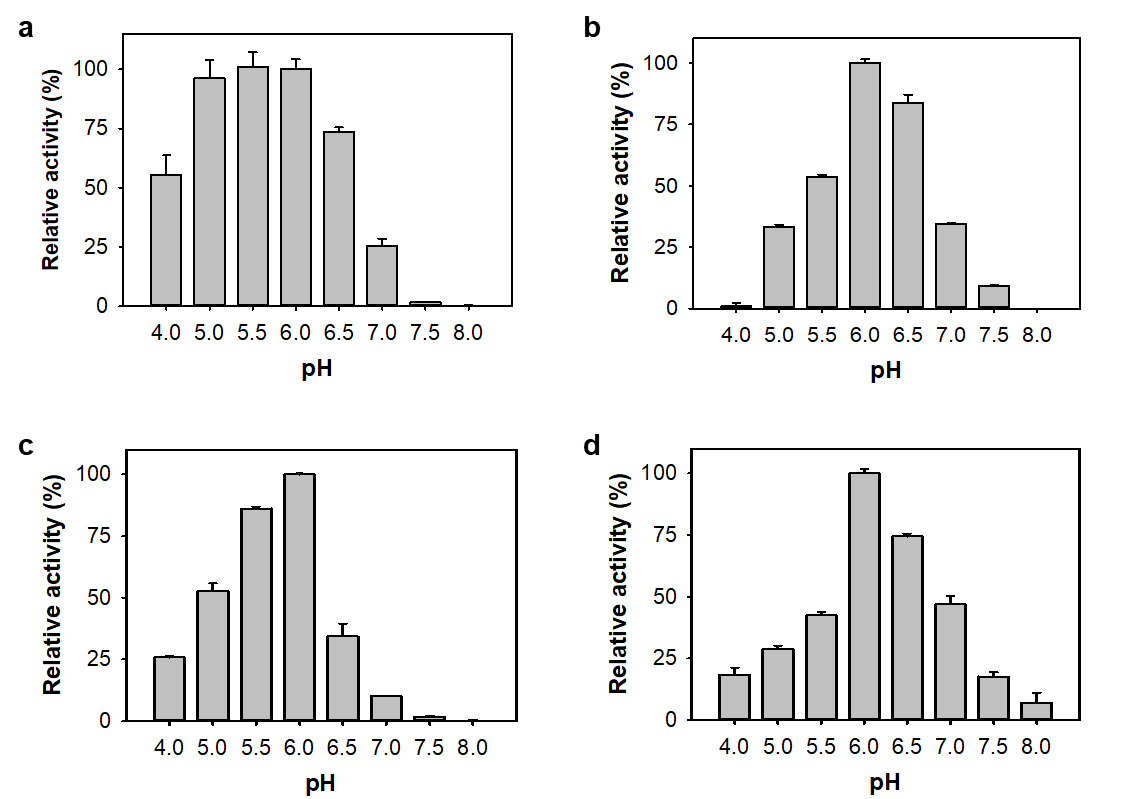


**Figure S40.** Effects of pH on one-pot cascade detection for a) glucose, b) lactate, c) choline, and d) cholesterol, using enzyme@RuNC_Cl composites.


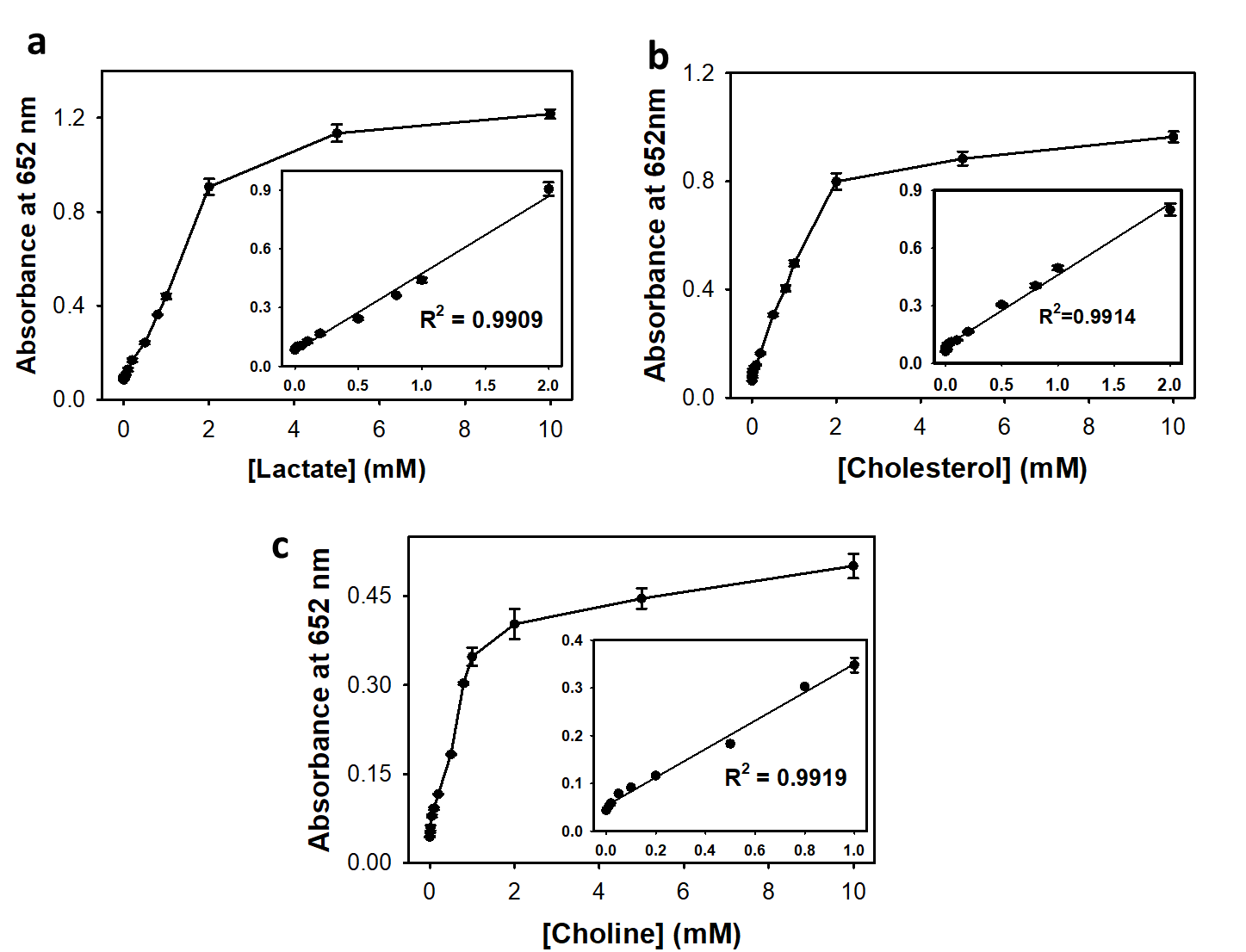


**Figure S41.** The dose-response of the detection using the RuNC_Cl in solution-based detection.

a) Lactate b) cholesterol c) choline.


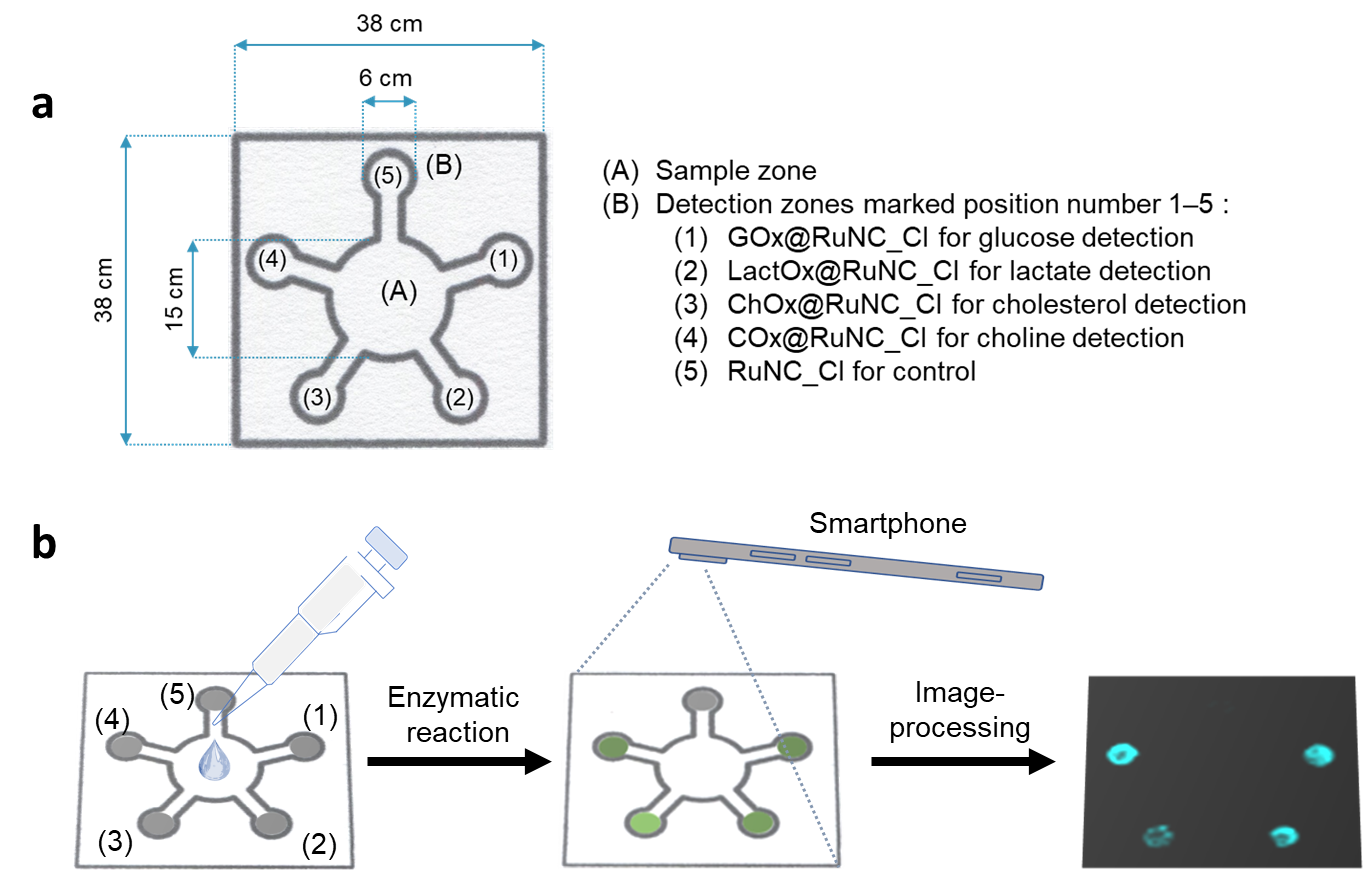


**Figure S42.** a) Design of RuNC_Cl-based paper microfluidic device (RuNC_Cl@µPADs) b) Schematic illustration of the detection process using RuNC_Cl@µPADs.


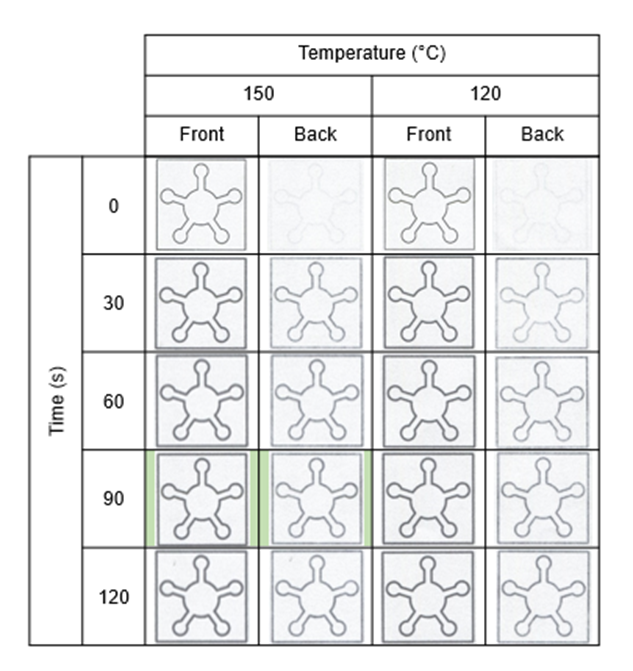


**Figure S43.** Optimization of wax melting temperature (120 °C and 150 °C) and time (0-120 s) of paper-based microfluidic devices with front and backside.


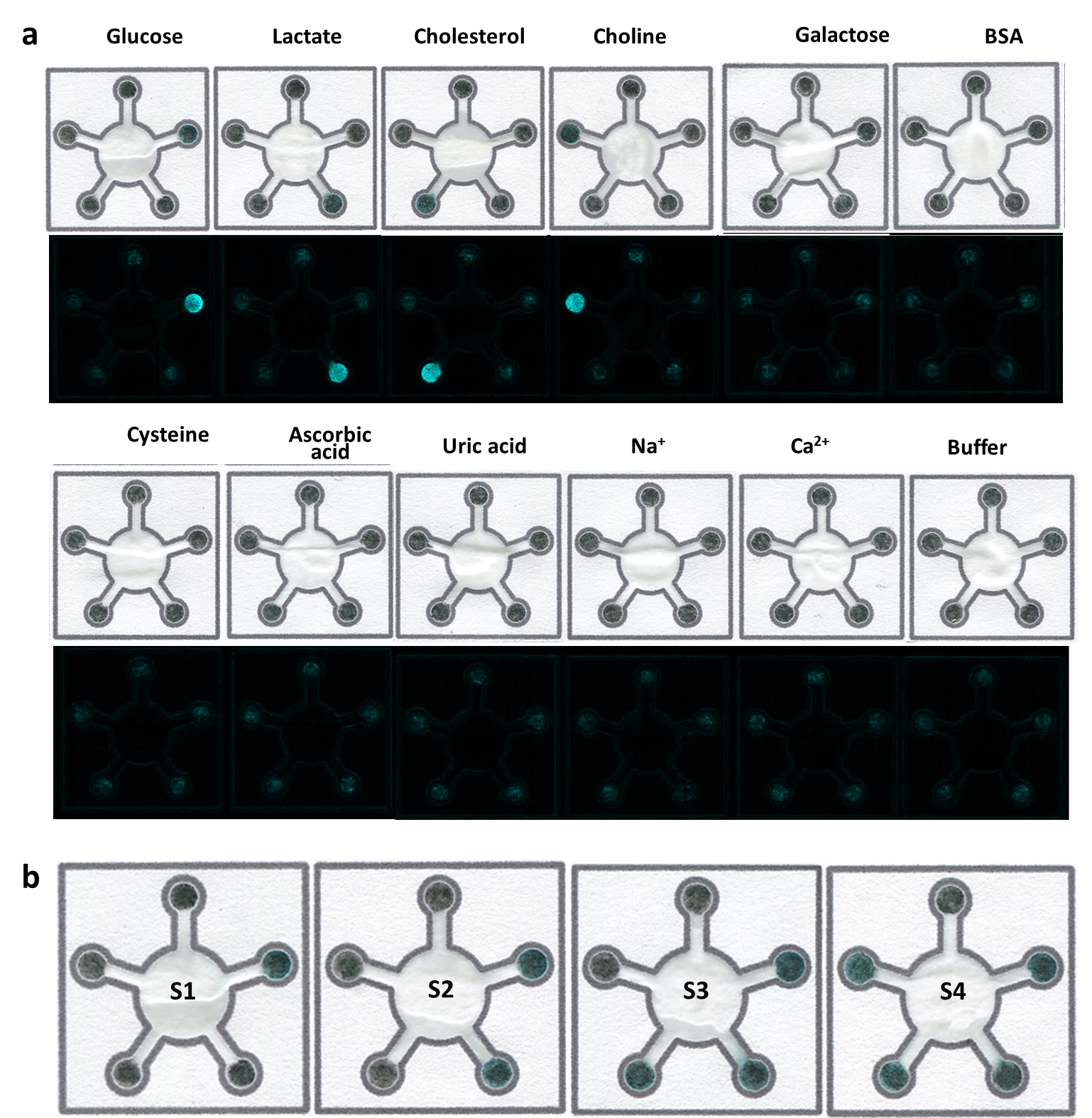


**Figure S44.** Detection images of a) single analyte and b) multiple biomarkers (S1: Glucose S2: Glucose + lactate S3: Glucose + lactate + cholesterol S4: Glucose + lactate + cholesterol + choline).


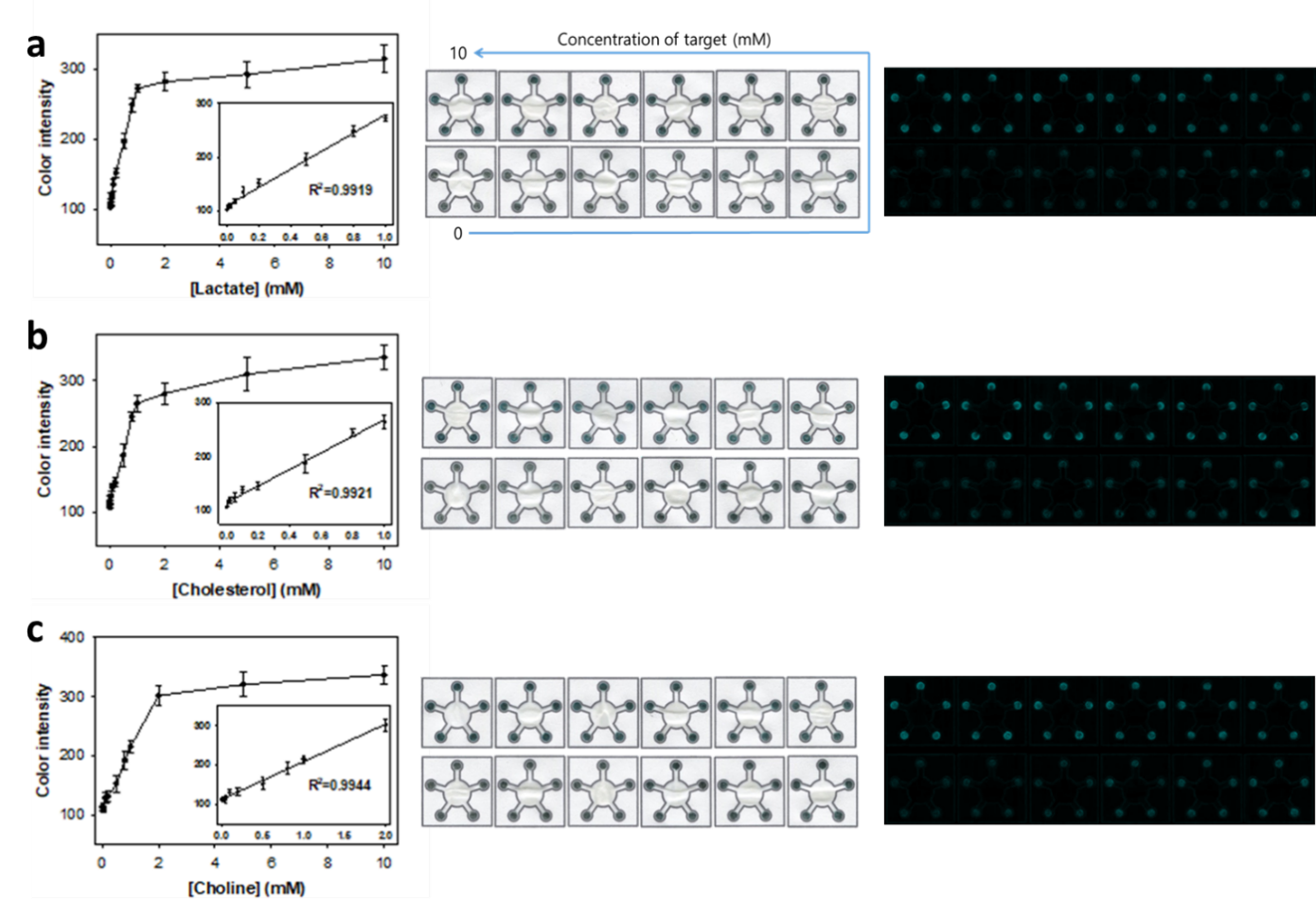


**Figure S45.** The dose-response curve and real images of the detection using the RuNC_Cl-based paper device.

a) Lactate b) cholesterol c) choline.


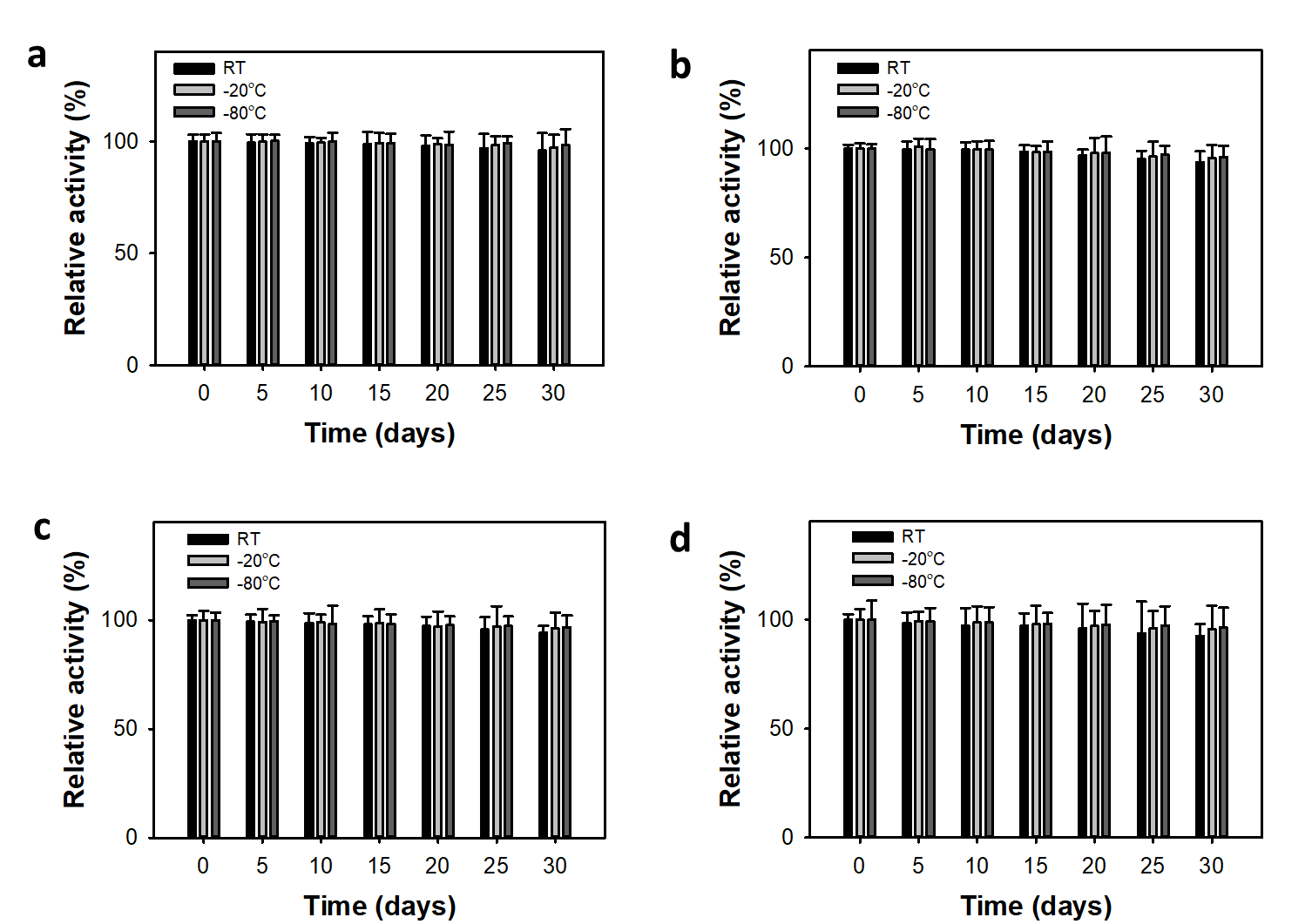


**Figure S46.** Storage stability of enzyme@RuNC_Cl.

The detection of H_2_O_2_ and the corresponding target molecules a) Glucose b) Lactate c) Cholesterol d) Choline.

| **Nanozymes** | **BET surface area [m^2^ g^-1^]** | **Pore diameter [nm]** | **Pore volume [cm^3^g^-1^]** |
| --- | --- | --- | --- |
| **N-MSUFC** | 596.52 | 3.73, 15.68 | 1.71 |
| **RuNC_Cl** | 581.71 | 3.73, 15.79 | 1.67 |
| **RuNC_C** | 574.75 | 3.74, 15.30 | 1.72 |
| **RuNC_O** | 558.65 | 3.73, 15.72 | 1.60 |

**Table S1.** Summary of Specific surface area, pore volume, and pore diameter (determined from BET analysis).

| **Metal (wt%)** | **RuNC_Cl** | **RuNC_C** | **RuNC_O** | **FeNC** |
| --- | --- | --- | --- | --- |
| **Ru** | 1.95 | 1.79 | 1.49 | - |
| **Fe** | - | - | - | 0.898 |

**Table S2.** Measured metal content using ICP-MS.

| **Sample** | **Path** | **CN** | **R(Å)** | **σ^2^ (Å^2^)** | **ΔE_0_ (eV)** | **R, %** |
| --- | --- | --- | --- | --- | --- | --- |
| **Ru foil** | Ru-Ru | 12 | 2.65 | 0.0033 | -5.85 | 0.016 |
| **RuNC_Cl** | Ru–N | 1.8 | 2.00 | 0.0065 | -4.98 | 0.001 |
|  | Ru-Cl | 2.0 | 2.28 | 0.0047 |  |  |
| **RuNC_C** | Ru-N | 0.9 | 1.99 | 0.0036 | 0.99 | 0.027 |
|  | Ru-C | 3.8 | 2.17 | 0.0035 |  |  |
| **RuNC_O** | Ru-N | 1.0 | 1.78 | 0.0196 | 5.12 | 0.016 |
|  | Ru-O | 4.0 | 2.13 | 0.0092 |  |  |
| **Fe foil** | Fe–Fe1 | 8 | 2.47 | 0.0045 | 7.68 | 0.005 |
|  | Fe-Fe2 | 6 | 2.86 | 0.0045 |  |  |
| **FeNC** | Fe-N | 4.0 | 1.99 | 0.0103 | -1.25 | 0.008 |

**Table S3.** EXAFS Fitting parameters of Ru K-edge and Fe K-edge**.**

| **Nanozymes** | **[E/Ru]**  **[M]** | **K_M_**  **[mM]** | **Vmax**  **[μM s^-1^]** | **k_cat_**  **[s^-1^]** | **k_cat_/K_m_**  **[s^-1^ mM^-1^]** |
| --- | --- | --- | --- | --- | --- |
| **RuNC_Cl** | 4.82×10^-8^ | 1200 | 0.672 | 13.9252 | 0.0116 |
| **RuNC_C** | 4.43×10^-8^ | 1681 | 0.6102 | 13.7043 | 0.0082 |
| **RuNC_O** | 7.37×10^-8^ | 924.9 | 0.2898 | 3.9320 | 0.0043 |
| **FeNC** | 8.04×10^-8^ | 1916 | 0.574 | 7.1392 | 0.0037 |

**Table S4.** POD-like kinetic parameters of Ru SAzymes and FeNC using H_2_O_2_ as substrate.

| **Nanozymes** | **1 cycle**  **Ru [ppm]** | **2 cycle**  **Ru [ppm]** | **3 cycle**  **Ru [ppm]** |
| --- | --- | --- | --- |
| **RuNC_Cl** | 0.00 | 0.00 | 0.00 |
| **RuNC_C** | 0.00 | 0.00 | 0.00 |
| **RuNC_O** | 0.00 | 0.00 | 0.00 |

**Table S5.** Ru contents of the supernatants after POD-like reaction measured by ICP-OES.

| **Nanozymes** | **[E/Ru]**  **[M]** | **K_M_**  **[mM]** | **V_max_**  **[μM s^-1^]** | **k_cat_**  **[s^-1^]** | **k_cat_/K_M_**  **[s^-1^ mM^-1^]** |
| --- | --- | --- | --- | --- | --- |
| **FeNC** | 9.65×10^-8^ | 0.2641 | 0.214 | 2.220 | 8.405 |

**Table S6.** POD-like kinetic parameters of FeNC using TMB as substrate.

| **Nanozymes** | **(E/Ru) [M]** | **K_M_**  **[mM]** | **V_max_**  **[mM s^-1^]** | **K_cat_**  **[s^-1^]** | **K_cat_/K_m_**  **[s^-1^ mM^-1^]** |
| --- | --- | --- | --- | --- | --- |
| **FeNC** | 8.04×10^-7^ | 114.5 | 0.0916 | 113.929 | 0.995 |

**Table S7.** CAT-like kinetic parameters of FeNC.

| **Analyte** | **Material** | **Working pH** | **Linearity (mM)** | **LOD (μM)** | **References** |
| --- | --- | --- | --- | --- | --- |
| H_2_O_2_ | FeN_3_/PtN_4_-SAzyme | 4.0 | 0.01–1.0 | 7.97 | ^[2]^ |
|  | FeNC | 4.0 | 0.01–0.6 | 4.36 | ^[3]^ |
|  | Zn/Mo DSAC-SMA | 3.0 | 0–3 | 24.4 | ^[4]^ |
|  | CeN_4_-SAzyme | 4.1 | 0.1–10 | 77 | ^[5]^ |
|  | FeNC | 6.0 | 0.019–0.156 | 8.67 | **This work** |
|  | **RuNC_Cl** | **6.0** | **0.019–1.25** | **1.78** |  |
| Glucose | Zn/Mo DSAC-SMA | 2-pH (5.0 and 3.0) | 0–1.8 | 15.6 | ^[4]^ |
|  |  | 1-pH (3.0) | 0–1 | 21.4 |  |
|  | Mo-Pt/CeO_2_ | 1-pH (4.0) | 0.01–0.2 | 2.46 | ^[6]^ |
|  | CeN_4_-SAzyme | 2-pH  (7.0 and 3.5) | 0.03–2 | 24 | ^[5]^ |
|  | GOx@FeNC | 1-pH (6.0) | 0.078–0.3125 | 17.00 | **This work** |
|  | **GOx@RuNC_Cl** | 1-pH (6.0) | **0.019–1.25** | **2.51** |  |
| Lactate | **LOx@RuNC_Cl** | 1-pH (6.0) | **0.01–2.0** | **8.76** | **This work** |
| Cholesterol | Zn/Mo DSAC-SMA | 2-pH  (7.4 and 3.0) | 0–0.25*10^-3^ | 7.44 | ^[4]^ |
|  | **ChOx@RuNC_Cl** | 1-pH (6.0) | **0.005–2.0** | **3.60** | **This work** |
| Choline | **COx@RuNC_Cl** | 1-pH (6.0) | **0.01–1.0** | **10.10** | **This work** |

**Table S8.** Comparison of biomarkers detection capability of RuNC_Cl with other reported SAzymes.

| **Analyte** | **Original  amount (mM)** | **Added  (mM)** | **Expected  (mM)** | **Measured ^a)^**  **(mM)** | **SD ^b)^** | **CV ^c)^ (%)** | **Recovery ^d)^**  **(%)** |
| --- | --- | --- | --- | --- | --- | --- | --- |
| Glucose | 2.55 | 0.3 | 2.85 | 2.86 | 0.10 | 3.39 | 100.19 |
| Lactate | 1.33 | 0.5 | 1.83 | 1.82 | 0.06 | 3.30 | 99.40 |
| Cholesterol | 1.65 | 0.2 | 1.85 | 1.86 | 0.06 | 3.13 | 100.76 |
| Choline | 0.05 | 0.5 | 0.55 | 0.57 | 0.03 | 5.35 | 102.79 |

^a)^ Average value of 5 successive measurements; ^b)^ Standard deviation (SD) of 5 measurements; ^c)^ Coefficient of variation = (SD / average) × 100; ^d)^ Recovery = (Measured value / Expected value) × 100

**Table S9.** Detection precision of RuNC_Cl well-plate assay kit.

| **Analyte** | **Original  amount (mM)** | **Added  (mM)** | **Expected  (mM)** | **Measured ^a)^**  **(mM)** | **SD ^b)^** | **CV ^c)^ (%)** | **Recovery ^d)^**  **(%)** |
| --- | --- | --- | --- | --- | --- | --- | --- |
| Glucose | 2.55 | 0.3 | 2.85 | 2.83 | 0.09 | 3.34 | 99.42 |
| Lactate | 1.33 | 0.5 | 1.83 | 1.82 | 0.13 | 7.25 | 99.57 |
| Cholesterol | 1.65 | 0.2 | 1.85 | 1.87 | 0.12 | 6.36 | 100.96 |
| Choline | 0.05 | 0.5 | 0.55 | 0.56 | 0.04 | 7.15 | 101.98 |

^a)^ Average value of 5 successive measurements; ^b)^ Standard deviation (SD) of 5 measurements; ^c)^ Coefficient of variation = (SD / average) × 100; ^d)^ Recovery = (Measured value / Expected value) × 100

**Table S10.** Detection precision of commercialized kits. Each kit was purchased from Sigma-Aldrich, and the assay was carried out following the manufacturer’s instructions.

| Target | RuNC_Cl-based platforms ($) | | | Commercialized kits ($) | |
| --- | --- | --- | --- | --- | --- |
|  | 1 mL 1 mg/mL enzyme@RuNC_Cl | Paper-based device | Well-plate kit | 100 tests | 1 test |
| Glucose | 0.30 | 0.0004 | 0.0015 | 442 | 4.42 |
| Lactate | 30.83 | 0.0370 | 0.1542 | 500 | 5 |
| Cholesterol | 24.45 | 0.0293 | 0.1223 | 545 | 5.45 |
| Choline | 1.91 | 0.0023 | 0.0096 | 513 | 5.13 |
| Total |  | 0.0690 | 0.2875 |  | 20 |

**Table S11.** Comparison of production costs calculated from the listed price in Sigma-Aldrich, between RuNC_Cl-based paper device and commercialized kits.

The detailed procedures for the calculation of the production cost for 1 test of glucose detection are summarized below.

1. A commercial kit for glucose detection (1 commercial kit can be used for 100 tests)

The price for 1 kit (100 tests) is 442 $.

The price for 1 test is 442 $ / 100 = 4.42 $.

1. RuNC_Cl-based glucose detection platforms

The price to synthesis 1 mL of 1 mg/mL RuNC_Cl is 0.0046 $

The price of GOx to synthesis 1 mL of 1 mg/mL GOx@RuNC_Cl is: 201 $ / 330 mg * 5 mg/mL * 0.1 mL= 0.3018 $ (1 bottle of GOx (containing ~330 mg GOx): 201 $. Need 0.1 mL of 5 mg/mL GOx to synthesis of 1 mL 1 mg/mL GOx@RuNC_Cl)

The price for 1 mL 1 mg/mL GOx@RuNC_Cl: 0.0046 + 0.3018 = 0.3064 $

1. 1 test using RuNC_Cl-based paper device needs:

1.2µL of 1 mg/mL GOx@RuNC_Cl = 0.3064 $ * (1.2 µL / 1000) = 0.0004 $

1.2 µL of TMB 10 mM = 54 $ / 1000 mg * 2.4 mg * 1 mL / (1.2 µL / 1000) = 0.0002$ (1 bottle containing 1 g TMB = 54 $, 1 mL of 10 mM TMB need 2.4 mg TMB)

1 paper = 85 $ / 100 sheets / 25 paper chip = 0.034 $ (1 pack = 100 sheets 20 cm * 20 cm = 85 $, 1 sheet of paper can make 25 paper chips)

Total price for 1 glucose test using 1 RuNC_Cl-based paper device: 0.0004 $ + 0.0002 $ + 0.034 $ = 0.0345 $

1. 1 test using RuNC_Cl-based well plate kit needs:
2. µL of 0.5 mg/mL GOx@RuNC_Cl: 0.3064 $ / (1 / 0.5) mg/mL * (20 µL / 1000) = 0.0031$
3. µL of TMB 10 mM: 54 $ / 1000 mg * 2.4 mg * 1 mL * (20 µL / 1000) = 0.0026 $

140 µL PB: 49.14 $ / 100 mL * (140 µL / 1000) = 0.0688 $ (1 bottle PB buffer 100 mL = 49.14 $)

Total price for 1 glucose test using 1 RuNC_Cl-based well-plate kit is 0.0031 $ + 0.0026 $ + 0.0688 $ = 0.0745 $

| **Analyte** | **Material** | **Working pH** | **Linearity (mM)** | **LOD (μM)** | **References** |
| --- | --- | --- | --- | --- | --- |
| Glucose | GOx + HRP | 2-pH  (7.0 and 5.6) | 0.02–4.0 | 14 | ^[7]^ |
|  | GOx + HRP | 1-pH (7.0) | 0.01–15 | 3 | ^[8]^ |
|  | GOx + HRP | - | 0.1–1.1 | 2800 | ^[9]^ |
|  | GOx@Co-m-ceria | 1-pH (6.0) | 0.1–1.5 | 7.0 | ^[10]^ |
|  | GOx + acetylene black-hemin | 2-pH  (7.0 and 4.0) | 0.2–30 | 60 | ^[11]^ |
|  | CuFe_2_O_4_@GOx | 2-pH  (7.4 and 4.4) | 0.1–50 | 40 | ^[12]^ |
|  | **GOx@RuNC_Cl** | 1-pH (6.0) | **0.01–2.0** | **6.0** | **This work** |
| Lactate | LOx + HRP | 1-pH (7.0) | 0.04–24 | 30 | ^[8]^ |
|  | **LOx@RuNC_Cl** | 1-pH (6.0) | **0.01–1.0** | **9.8** | **This work** |
| Cholesterol | ChOx@Co-m-ceria | 1-pH (6.0) | 0.1–1.5 | 8.0 | ^[10]^ |
|  | ChE + ChOx + N-CD | 1-pH (3.0) | 2.5–7.5 | 676 | ^[13]^ |
|  | ChE + ChOx + NSC/Co_1-x_S | 2 pH  (7.0 and 3.6) | 0.2–3.0 | 60 | ^[14]^ |
|  | ChOx + V-porous Co_3_O_4_ | 1-pH (3.0) | 0.1–10 | 28.1 | ^[15]^ |
|  | ChOx + HRP | 1-pH (4.0) | 1.0–10.0 | 570 | ^[16]^ |
|  | **ChOx@RuNC_Cl** | 1-pH (6.0) | **0.02–1.0** | **10.7** | **This work** |
| Choline | COx + HRP | 1-pH (7.0) | 0.04–24 | 10 | ^[8]^ |
|  | COx@Co-m-ceria | 1-pH (6.0) | 0.1–2.0 | 7.0 | ^[10]^ |
|  | **COx@RuNC_Cl** | 1-pH (6.0) | **0.01–2.0** | **5.8** | **This work** |

**Table S12.** Comparison of biomarkers detection capability of RuNC_Cl-based paper devices with other reported paper devices.

| **Analyte** | **Original  amount (mM)** | **Added  (mM)** | **Expected  (mM)** | **Measured ^a)^**  **(mM)** | **SD ^b)^** | **CV ^c)^ (%)** | **Recovery ^d)^**  **(%)** |
| --- | --- | --- | --- | --- | --- | --- | --- |
| Glucose | 2.25 | 0.5 | 2.75 | 2.78 | 0.24 | 8.52 | 101.25 |
| Lactate | 1.52 | 0.5 | 2.02 | 2.07 | 0.05 | 2.57 | 102.36 |
| Cholesterol | 1.32 | 0.5 | 1.82 | 1.81 | 0.09 | 5.20 | 99.28 |
| Choline | 0.04 | 0.5 | 0.54 | 0.55 | 0.06 | 11.57 | 100.97 |

^a)^ Average value of 5 successive measurements; ^b)^ Standard deviation (SD) of 5 measurements; ^c)^ Coefficient of variation = (SD / average) × 100; ^d)^ Recovery = (Measured value / Expected value) × 100

**Table S13.** Detection precision of RuNC_Cl paper microfluidic device.

Video S1 is separately uploaded.

**Video S1.** Color development of GOx@RuNC_Cl and GOx@FeNC for off-device (solution-based) glucose detection.

Video S2 is separately uploaded.

**Video S2.** Color development of RuNC_Cl and FeNC-based paper devices for glucose detection.

**References**

[1] J. Lee, D. Lee, E. Oh, J. Kim, Y. P. Kim, S. Jin, H. S. Kim, Y. Hwang, J. H. Kwak, J. G. Park, *Angew. Chem. Int. Ed.* **2005**, 117, 7593.

[2] S. Wang, Z. Hu, Q. Wei, H. Zhang, W. Tang, Y. Sun, H. Duan, Z. Dai, Q. Liu, X. Zheng, *Nano Res.* **2022**, 15, 4266.

[3] W. Lu, S. Chen, H. Zhang, J. Qiu, X. Liu, *J. Materiomics.* **2022**, 8, 1251.

[4] C. B. Ma, Y. Xu, L. Wu, Q. Wang, J. J. Zheng, G. Ren, X. Wang, X. Gao, M. Zhou, M. Wang, *Angew. Chem. Int. Ed.* **2022**, 61, e202116170.

[5] Q. Chang, J. Wu, R. Zhang, S. Wang, X. Zhu, H. Xiang, Y. Wan, Z. Cheng, M. Jin, X. Li, *Nano Today* **2024**, 56, 102236.

[6] L. Ge, Y. Chen, B. Geng, X. Chu, R. Jiang, X. Wang, X. Qin, W. Li, S. Song, *Small* **2024**, 20, 2404608.

[7] X. Wang, F. Li, Z. Cai, K. Liu, J. Li, B. Zhang, J. He, *Anal. Bioanal. Chem.* **2018**, 410, 2647.

[8] F. Li, X. Wang, J. Liu, Y. Hu, J. He, *Sens. Actuators B: Chem.* **2019**, 288, 266.

[9] H. Zhang, Z. Chen, J. Dai, W. Zhang, Y. Jiang, A. Zhou, *Microchem. J.* **2021**, 162, 105814.

[10] P. T. Nguyen, J. Lee, A. Cho, M. S. Kim, D. Choi, J. W. Han, M. I. Kim, J. Lee, *Adv. Funct. Mater.* **2022**, 32, 2112428.

[11] X. Yi, Y. Yuan, M. Qing, L. Wang, H. Li, L. Bai, *Spectrochim. Acta A Mol. Biomol. Spectrosc.* **2023**, 296, 122667.

[12] Y. Ren, X. Zuo, Z. Liu, M. Yu, B. Zhou, Y. Xue, L. Zhu, R. Yang, *Microchem. J.* **2024**, 206, 111504.

[13] N. Kitchawengkul, A. Prakobkij, W. Anutrasakda, N. Yodsin, S. Jungsuttiwong, S. Chunta, M. Amatatongchai, P. Jarujamrus, *Anal. Chem.* **2021**, 93, 6989.

[14] J. Li, T. Liu, R. A. Dahlgren, H. Ye, Q. Wang, Y. Ding, M. Gao, X. Wang, H. Wang. *Anal. Chim. Acta.* **2022**, 1204, 339703.

[15] B. Wongsing, A. Prakobkij, W. Anutrasakda, P. Jarujamrus, *Anal. Chem.* **2022**, 94, 13785.

[16] A. Prakobkij, S. Sukapanon, S. Chunta, P. Jarujamrus, *Anal. Chim. Acta.* **2023**, 1263, 341303.
